# An acetyltransferase effector conserved across *Legionella* species targets the eukaryotic eIF3 complex to modulate protein translation

**DOI:** 10.1128/mbio.03221-23

**Published:** 2024-02-09

**Authors:** Lukas Syriste, Deepak T. Patel, Peter J. Stogios, Tatiana Skarina, Dhruvin Patel, Alexei Savchenko

**Affiliations:** 1 Department of Microbiology, Immunology and Infectious Diseases, University of Calgary, Calgary, Alberta, Canada; 2 Department of Chemical Engineering and Applied Chemistry, Toronto University, Toronto, Ontario, Canada; Institut Pasteur, Paris, France

**Keywords:** effectors, acetyltransferase, pathogenesis, *Legionella pneumophila*

## Abstract

The survival of *Legionella* spp. as intracellular pathogens relies on the combined action of protein effectors delivered inside their eukaryotic hosts by the Dot/Icm (defective in organelle trafficking/intracellular multiplication) type IVb secretion system. The specific repertoire of effector arsenals varies dramatically across over 60 known species of this genera with *Legionella pneumophila* responsible for most cases of Legionnaires’ disease in humans encoding over 360 Dot/Icm effectors. However, a small subset of “core” effectors appears to be conserved across all *Legionella* species raising an intriguing question of their role in these bacteria’s pathogenic strategy, which for most of these effectors remains unknown. *L. pneumophila* Lpg0103 effector, also known as VipF, represents one of the core effector families that features a tandem of Gcn5-related N-acetyltransferase (GNAT) domains. Here, we present the crystal structure of the Lha0223, the VipF representative from *Legionella hackeliae* in complex with acetyl-coenzyme A determined to 1.75 Å resolution. Our structural analysis suggested that this effector family shares a common fold with the two GNAT domains forming a deep groove occupied by residues conserved across VipF homologs. Further analysis suggested that only the C-terminal GNAT domain of VipF effectors retains the active site composition compatible with catalysis, whereas the N-terminal GNAT domain binds the ligand in a non-catalytical mode. We confirmed this by *in vitro* enzymatic assays which revealed VipF activity not only against generic small molecule substrates, such as chloramphenicol, but also against poly-L-lysine and histone-derived peptides. We identified the human eukaryotic translation initiation factor 3 (eIF3) complex co-precipitating with Lpg0103 and demonstrated the direct interaction between the several representatives of the VipF family, including Lpg0103 and Lha0223 with the K subunit of eIF3. According to our data, these interactions involve primarily the C-terminal tail of eIF3-K containing two lysine residues that are acetylated by VipF. VipF catalytic activity results in the suppression of eukaryotic protein translation *in vitro*, revealing the potential function of VipF “core” effectors in *Legionella*’s pathogenic strategy.

**IMPORTANCE:**

By translocating effectors inside the eukaryotic host cell, bacteria can modulate host cellular processes in their favor. *Legionella* species, which includes the pneumonia-causing *Legionella pneumophila,* encode a widely diverse set of effectors with only a small subset that is conserved across this genus. Here, we demonstrate that one of these conserved effector families, represented by *L. pneumophila* VipF (Lpg0103), is a tandem Gcn5-related N-acetyltransferase interacting with the K subunit of human eukaryotic initiation factor 3 complex. VipF catalyzes the acetylation of lysine residues on the C-terminal tail of the K subunit, resulting in the suppression of eukaryotic translation initiation factor 3-mediated protein translation *in vitro*. These new data provide the first insight into the molecular function of this pathogenic factor family common across *Legionellae*.

## INTRODUCTION

*Legionella pneumophila* is a Gram-negative facultative intracellular pathogen that causes severe pneumonia, known as Legionnaires’ disease, in humans by infecting alveolar macrophages (1, 2). Along with other representatives of the *Legionella* genus, which includes over 60 known species, *L. pneumophila* parasitizes primarily on a broad host range of freshwater and soil protozoa, while human infection happens through the inhalation of contaminated water droplets (3). *L. pneumophila* employs the Dot/Icm (defective in organelle trafficking/intracellular multiplication) type IVb secretion system to translocate the largest known arsenal of protein effectors inside its eukaryotic host cells (4–6). The combined action of these pathogenic factors allows *L. pneumophila* to manipulate host cellular processes, such as cytoskeleton dynamics, autophagy, and vesicular trafficking, to prevent lysosomal degradation after being phagocytized and to form a permissive replicative compartment inside the host cell called the *Legionella-*containing vacuole (LCV) (7). The presence of a large number of effectors is indicative of functional redundancy across *L. pneumophila* pathogenic arsenal (8, 9). This poses a significant challenge for the characterization of the activity of an individual effector by a forward genetic approach since the deletion of a single effector gene rarely leads to a trackable phenotype. Accordingly, despite significant progress in the identification of *L. pneumophila* effector functions, many of these pathogenic factors remain uncharacterized.

Large-scale genomic studies into the *Legionella* genus have identified significant variation in Dot/Icm translocated effector repertoire across different species of this genera, estimating the combined number of *Legionella* effectors to be around 18,000 (10, 11). These studies also suggested that a small subset of effectors is encoded by all characterized *Legionella* genomes (10, 11). According to the most recent reports, there are now nine such “core” effector families, represented by *L. pneumophila* Lpg0086 (LceA), Lpg0103 (VipF), Lpg0107 (RavC), Lpg0140 (CetLP1), Lpg1356 (LceB), Lpg2300 (AnkH), Lpg2815 (MavN), Lpg2832, and Lpg3000 proteins (12). The absolute conservation of these effectors in *Legionella* species suggests an important role for these pathogenic factors in the remarkable ability of *L. pneumophila* to infect evolutionally distant eukaryotic hosts, such as unicellular protozoa and human alveolar macrophages.

Members of the “core” *Legionella* effector families have been the focus of several recent studies revealing certain aspects of their activity inside the host cell. Specifically, *L. pneumophila* MavN (Lpg2815) has been reported to reside in the LCV membrane and facilitate the acquisition of iron through an uncharacterized molecular mechanism (13, 14). The crystal structure of AnkH (Lpg2300) has revealed a modular architecture with N-terminal ankyrin repeats, followed by a cysteine protease-like fold and C-terminal α-helical domain (15). AnkH has been shown to interact with human LARP7, a regulatory component of RNA polymerase II complex, which leads to a global change in the transcriptome of infected eukaryotic cells (15). The essentiality of these two “core” effectors is further highlighted by the severe growth defect phenotype associated with single gene deletion strains of *L. pneumophila* lacking either AnkH or MavN (13–15). Notably, such drastic phenotype has not been observed in the case of single gene deletion of other “core” effector genes (12, 16).

Our knowledge about the remaining *Legionella* “core” effector families remains sparse. One of these enigmatic “core” effectors is represented by *L. pneumophila* VipF (Lpg0103)—a long-recognized member of the Gcn5-related N-acetyltransferases (GNATs) superfamily, which encompasses enzymes present in all kingdoms of life (17). GNATs catalyze the transfer of an acetyl group from an acetyl-coenzyme A (acetyl-CoA) donor to the primary amine or hydroxyl group of a substrate, which can be small molecules, peptides, or proteins (18). The presence of GNAT-specific sequence motifs allows for accurate prediction of this enzymatic domain from primary sequence analysis; however, the specific activity of these enzymes must be determined empirically. The sequence analysis of Lpg0103 revealed the presence of two distinct GNAT domains, thus suggesting that the representatives of the VipF family belong to a small subset of so-called tandem GNATs. Only a few tandem GNAT enzymes have been biochemically and structurally characterized thus far, including mycothiol synthase MshD (PDB: 1OZP) (19), clavulanic acid biosynthetic gene cluster clavulanic acid biosynthesis gene cluster encodes for an acetyl transferase (CBG) (PDB: 2WPX) (20), and glucosamine N-acetyltransferase GlmA (PDB: 5KF1) (21). The evolutionary rationale resulting in the duplication of the GNAT fold is unclear, as only one of the GNAT domains appears to be catalytically active in tandem enzymes characterized so far (17). A previous report suggested that both GNAT domains of Lpg0103 are active against chloramphenicol substrate. However, a more recent report established that VipF follows the general trend of tandem GNATs with only the C-terminal domain retaining the catalytic activity (22, 23). Despite these insights into the biochemical activity of VipF, the significance of its acetyltransferase activity function for *Legionella*’s invasion of the host cell has never been elucidated.

Here, we present a comprehensive structural and functional analysis of VipF family representatives where we determine the crystal structure of the VipF ortholog from *Legionella hackeliae* (Lha0223) in complex with acetyl-CoA. We confirm that the acetyltransferase activity of Lpg0103 and Lha0223 is localized to the C-terminal GNAT domain and demonstrate that these VipF effectors are active against small molecules and peptide substrates. We then demonstrate that VipF effectors interact with the eukaryotic translation initiation factor 3 (eIF3) complex through direct interactions with the K subunit (eIF3-K). Finally, we demonstrate that VipF can acetylate eIF3-K and that this acetylation event leads to a dampening of protein translation.

## RESULTS

### The crystal structure of VipF reveals duplication of the GNAT fold with distinct structural differences between the two domains

The phylogenetic reconstruction revealed that VipF homologs in *Legionella* species shared between 35% and 83% of primary sequence identity, suggesting a significant diversification across this effector family. Along with previous data (23), our analysis of the primary sequences of VipF orthologs confirmed the presence of conserved motifs corresponding to two GNAT domains typically found in a small subfamily, called tandem GNAT enzymes (Fig. S1A). Notably, our phylogenetic reconstruction also suggested an evolutionary relationship between VipF effectors and the predicted GNAT protein ACA1_384820 encoded by *Acanthamoeba castellanii* (Fig. S1B), which share only 16% of primary sequence identity with Lpg0103. This observation is in line with the previously postulated hypothesis of a link between the effector arsenal of *Legionella* and their environmental hosts (24).

To gain further insight into the molecular function of *Legionella* VipF effectors, we pursued the structural characterization of selected representatives of this effector family. Specifically, in addition to Lpg0103, we purified and submitted for crystallization the *L. hackeliae* Lha0223 (47% sequence identity to Lpg0103), *Legionella micdadei* Lmi3006 (50% ID), *Legionella longbeachae* Llo3312 (61% ID), and *Legionella falloni* Lfa0162 (62% ID) (Fig. S1A). However, structure determination diffraction quality crystals were only obtained in the case of Lha0223 in the presence of either acetyl-CoA or CoA.

**Fig 1:**
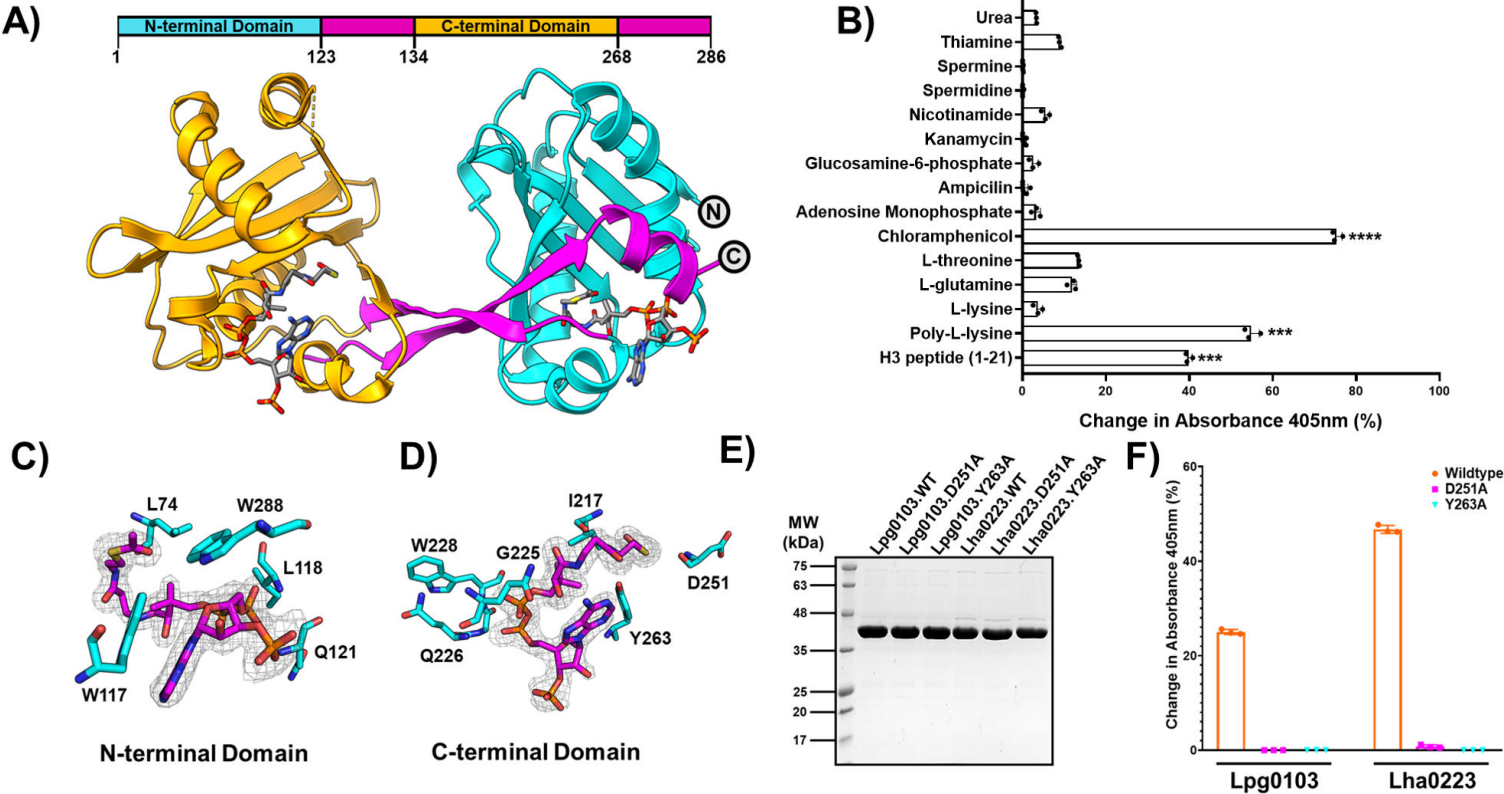
VipF is a tandem GNAT acetyltransferase. (**A**) Domain organization is shown on the top and crystal structure of Lha0223 (PDB 6WQB) where the N-terminal domain is colored cyan while the C-terminal domain is colored orange and both splayed β-strands are colored magenta. (**B**) General acetyltransferase substrate screen using the 5´5´-dithiobis(2-nitrobenzoic acid) (DTNB)-based assay. The experiments were performed in three independent experiments with similar results. Statistical analysis for the DTNB assays was performed with a two-tailed *t*-test (***, *P* < 0.004; ****, *P* < 0.0001). (**C** and **D**) A comparison of the residues (cyan) involved in coordinating acetyl coenzyme A (AcCoA) (magenta) between the C-terminal and N-terminal domains. (**E**) Coomassie stain of purified wild-type (WT) enzymes and their mutants used for 1B and 1F. (**F**) Alanine substitutions of Asp251 and Tyr263 in both Lpg0103 and Lha0223 were tested for activity against the histone H3 peptide using the DTNB-based detection assay.

Accordingly, the crystal structure of the Lha0223/CoA complex was determined at 2.3 Å resolution by single-wavelength anomalous dispersion using a selenomethionyl derivatized protein sample. Next, the structure of the Lha0223/acetyl-CoA complex was determined and refined to 1.75 Å resolution by molecular replacement using Lha0223/CoA as a model (Fig. 1A). In the case of both complex structures, the asymmetric unit contained the electron density corresponding to one VipF polypeptide chain spanning residues 1 to 286 and a portion of the N-terminal fusion tag corresponding to the Tobacco Etch Virus Protease (TEV) cleavage sequence (GQENLYFQG) (see Materials and Methods for details). However, we were unable to obtain the proper density to assign E173 on the Lha0223/acetyl-CoA complex. The superimposition of both structure complexes revealed an Root-mean-square deviation (RMSD) value of 0.645, suggesting that both structures are highly similar and do not undergo significant conformational changes in the presence of different ligands. The statistics of the final crystal structures of the Lha0223 complexes, which were deposited to the Research Collaboratory for Structural Bioinformatics (RCSB) Protein Data Bank, are summarized in Table S1.

In line with sequence analysis, our structures revealed a two-domain architecture in which both N-terminal (residues 1–123) and C-terminal (residues 135–268) domains adopt a mixed α/β fold characteristic of the GNAT superfamily (Fig. 1A). Recently, the structure of Lpg0103/VipF in complex with CoA and chloramphenicol was reported (23). The defined structure of Lha0223 acetyl-CoA complex superimposes with Lpg0103 complex structure with RMSD of 1.08 Å over 264 α-carbons, thus suggesting that VipF orthologs, despite having significant variation in primary sequence, share a conserved overall structure (Fig. S2). As previously described for Lpg0103 structure, the last β-strand in each of the Lha0223’s GNAT domains cross-pass to form an antiparallel β-sheet that provides enough continuity to link both GNAT lobes together (Fig. 1A). The dyad symmetry of this enzyme results in a large continuous central cleft (approximately 2,300 Å^3^) with a negatively charged oxyanion channel opening into the acetyl-CoA binding pocket of C-terminal GNAT domain. Although the N-terminal GNAT domain of Lha0223/acetyl-CoA complex structure also contained the density corresponding to one acetyl-CoA molecule, it had no connection to the central tunnel (Fig. S3A and B).

The N- and C-terminal domains of the Lha0223 structure superimpose with RMSD of 3.7 Å over 124 α-carbons while sharing less than 14% of primary sequence identity. Despite the structural similarity, we observed important differences in ligand binding. Both lobes contain a conserved P-loop motif [Q/R-X-X-G-X-G/A] to coordinate the pyrophosphate moiety of acetyl-CoA, composed of an N-terminal R80-R81-N82-G83-I84-A85 and C-terminal Q224-G225-Q226-G227-W228-G229 sequences. However, the acetyl group of acetyl-CoA in the N-terminal domain localized to an occluded, hydrophobic pocket that would be inaccessible to the incoming co-substrate and lacks residues that could act as a general acid and base for catalysis (Fig. S3B) typical of enzymatically active GNAT representatives. The side chains of L74, W117, and W288 enclose the acetyl moiety, contributing to the hydrophobic nature of this pocket (Fig. 1C; Fig. S3B). In contrast, the acetyl-CoA molecule bound in the C-terminal domain localized to a solvent-exposed cleft with its sulfur atom positioned close to the conserved residues Y263 and D251, which can act as a general acid and base, respectively (Fig. 1D; Fig. S3A). Similar conformational differences between acetyl-CoA binding have been observed in other structurally characterized tandem GNAT proteins, such as MshD which superimposes with Lha0223 structure with RMSD of 4.1 Å over 264 α-carbons (PDB: 1OZP) (Fig. S4A and B). In the MshD structure visualized in complex with acetyl-CoA, the conformation of this co-substrate in the hydrophobic pocket of the N-terminal lobe is also unfavorable for catalysis, resulting in only the C-terminal domain of this GNAT enzyme being catalytically active (19). Along the same lines, the C- rather than N-terminal domain in Lha0223-ligand complexes showed strong structural similarity to structurally characterized non-tandem GNAT enzyme-ligand complexes, such as archaeal amino-terminal acetyltransferase (NAT) from *Saccharolobus solfataricus* (PDB: 4LX9) and the RimI GNAT from *Salmonella typhimurium* (PDB: 2CNM) (Fig. S4C and D) (25, 26). Based on this analysis, we hypothesized that only the C-terminal domain in Lha0223 is catalytically active and this feature is shared by other VipF effectors.

### VipF demonstrates activity against small molecule and peptide substrates mediated by a catalytic acid/base dyad

Since the catalytic properties of VipF effectors beyond Lpg0103 have not been investigated, we tested Lha0223 acetyltransferase activity against an array of small molecule substrates as previously described (27). Among tested substrates, we observed that Lha0223 activity was highest against chloramphenicol, in line with the general activity reported for *L. pneumophila* VipF/Lpg0103 (Fig. 1B) (22, 23). However, Lha0223 also demonstrated strong activity against poly-lysine substrate suggesting that it can be active against peptide or protein targets. A similar assay demonstrated that Lpg0103 also showed activity against this peptide (Fig. S5) and that both Lpg0103 and Lha0223 showed robust activity against a short peptide corresponding to residues 1–21 of human histone H3—a standard substrate for eukaryotic protein acetyltransferases (Fig. 1B; Fig. S5) (28). These data demonstrate that, despite sequence variation, the two tested VipF effectors share a common acetyltransferase activity profile against small molecule and peptide substrates.

The similarity in biochemical activity between VipF effectors from *L. hackeliae* and *L. pneumophila* suggested that these enzymes share common active site features. Consequently, we used sequence conservation between these orthologs as our guide to identifying the role of specific residues for catalysis and substrate binding (Fig. S1A). We focused our analysis on the C-terminal domain since, only in this domain, the position of the co-substrate was in line with the acetyl transfer mechanism typically demonstrated by GNAT enzymes (Fig. S3A).

Direct nucleophilic attack of acetyl-CoA by an acceptor amine is the predominant mechanism of acetyl transfer in many characterized GNAT enzymes (17). This mechanism relies on the presence of an active site acid to protonate the sulfhydryl group of acetyl-CoA following the breakdown of the tetrahedral intermediate. Most GNAT enzymes using this mechanism contain conserved Tyr and Glu/Asp residues in the active site positioned proximal to acetyl-CoA. As mentioned above, the analysis of Lha0223 complex structures identified Y263, which is located at the distal end of α4 helix, as a possible candidate for catalytic general acid. The R-group of this residue is positioned directly underneath the sulfur atom of acetyl-CoA at a distance of 3.7 Å. In the anti-parallel β-sheet that connects both GNAT domains of Lha0223, D251 occupies a position immediately adjacent to the active site, suggesting that this is potentially the general base. The comparative sequence analysis within *Legionella* VipF orthologs demonstrated the complete conservation of these two residues (Fig. S1A).

To investigate the involvement of these residues in VipF catalysis, we constructed and purified Lha0223 and Lpg0103 variants carrying D251A or Y263A mutation (Fig. 1E). Substitution of both tyrosine and aspartate residues resulted in complete loss of *in vitro* activity of these VipF effectors against the histone H3 peptide (residues 1–21) (Fig. 1F). These results are in line with our structural analysis, indicating that only the C-terminal domain of the VipF effectors is responsible for observed *in vitro* acetyltransferase activity, which was also shown in a previous report of Lpg0103 (23).

### VipF effectors engage with the host eIF3 complex by directly interacting with the eIF3-K subunit

**Fig 2:**
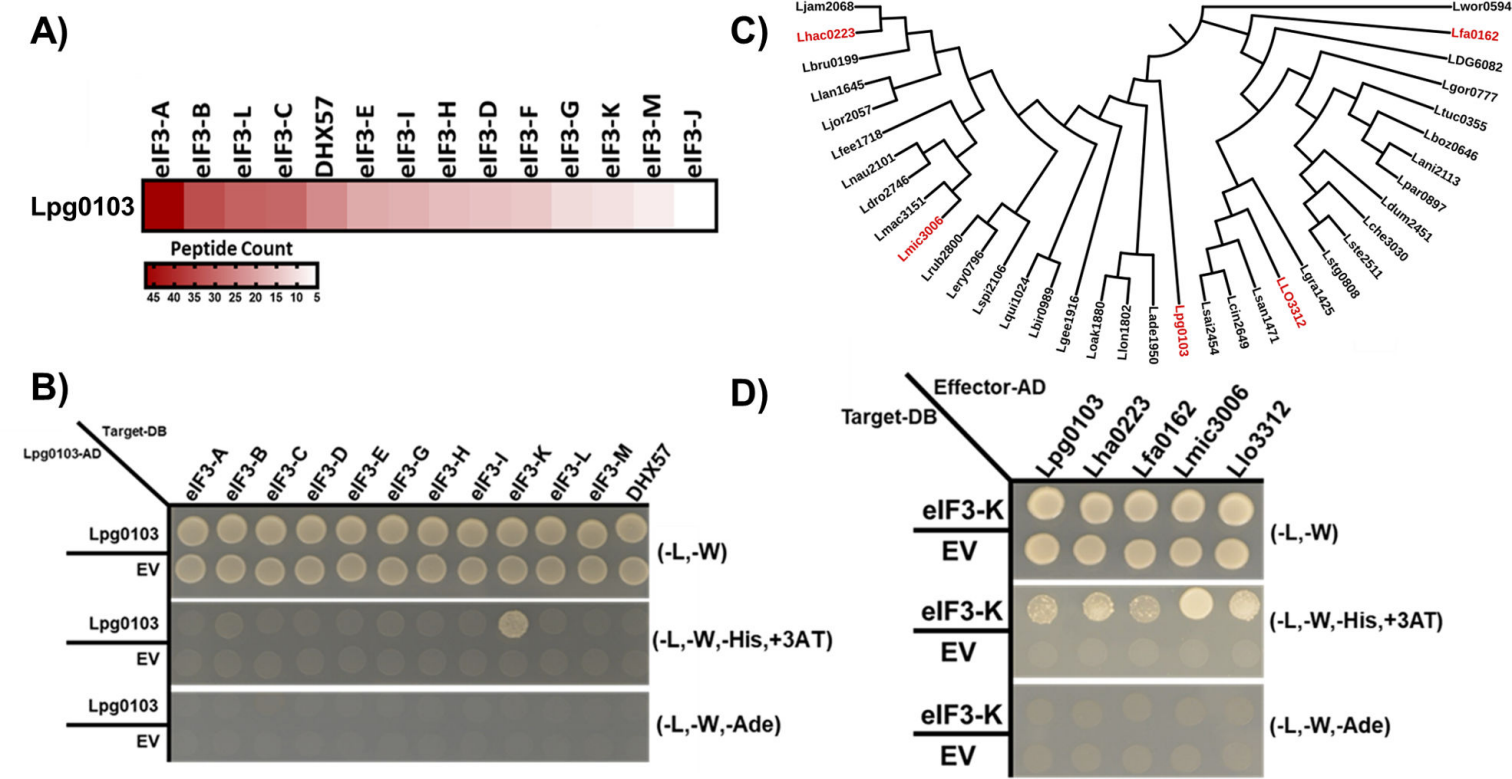
The human putative protein targets of VipF. (**A**) The putative human protein targets of VipF were identified using Affinity purification followed by mass spectrometry (AP-MS) with U937 cell lysate. The heat map representation of the AP-MS results is displayed as the average total spectrum peptide counts in at least nine MS runs. (**B**) A yeast two-hybrid (Y2H) experiment was performed for all subunits of the eIF3 complex and DHX57 in order to determine direct interactions between VipF and candidate human protein targets, confirming that VipF interacts with eIF3-K. (**C**) A maximum-likelihood phylogenetic tree of 37 VipF orthologs was generated using the amino acid sequence. The phylogenetic tree was constructed with IQ-TREE and visualized using iTOL. (**D**) Y2H experiment of VipF orthologs with low to high sequence similarity to Lpg0103, demonstrating that orthologs of VipF can interact with eIF3-K. The AP-MS and Y2H experiments were performed in three independent experiments with similar results.

The biological function of bacterial effectors typically requires engagement with the host proteome. To gain a better understanding of the function of VipF effectors, we searched for possible human protein interaction partners for Lpg0103 using mass spectrometry-assisted affinity purification (see Materials and Methods for details). In brief, Lpg0103 or Lha0223 effectors immobilized on streptavidin-coated beads were co-incubated with human U937 cell lysates. Precipitates from beads with no effector proteins or carrying the unrelated previously characterized effector NleG8-1 from enterohemorrhagic *Escherichia coli* (29) were used as controls (Fig S6A; Table S2). In addition, human proteins identified in the Lpg0103 and Lha0223 co-precipitates from Table S2 were also cross-checked for common human proteins with non-specific binding using the contaminant repository for affinity purification (CRAPome, https://reprint-apms.org/) database and were not considered for further analysis. This analysis suggested that samples co-precipitated with both Lpg0103 and Lha0223 effectors are consistently and specifically enriched with all 13 subunits comprising the human translation initiation factor 3 (eIF3) complex, including the DEAD-box helicase 57 that is also part of the eIF3 complex (Fig. 2A; Table S2). The eIF3 complex is considered the largest initiation factor serving as a scaffold in organizing other initiation factors on the surface of the 40S ribosome subunit (30). Of particular importance, the eIF3 complex is indispensable for eukaryotic cell viability and, while varying in specific subunit composition, is broadly conserved across eukaryote species, including protozoa (31, 32). Analysis of the Lpg0103 co-precipitation complex also identified other human proteins not associated with the eIF3 complex, such as the AHNAK nucleoprotein and Homer protein homolog 3 (Fig. 2A; Table S2).

To determine if Lpg0103 is involved in direct interaction with any of the individual eIF3 complex subunits, we tested the pairwise interaction yeast two-hybrid (Y2H). Out of 11 eIF3 subunits that we tested with Lpg0103, the co-expression of only subunit K (eIF3-K) resulted in the growth of yeast on the interaction selective medium lacking histidine, which selects for less stringent interactions (Fig. 2B). Furthermore, based on western blot analysis, we were unable to observe expression of eIF3-A, eIF3-C, and eIF3-H; therefore, the potential of interaction with these subunits will require further investigation (Fig. S7). Notably, no growth phenotype was observed for Lpg0103 co-expression with subunit K on the selective medium lacking adenine. Since such growth phenotype on a medium lacking adenine selects for more stable and tightly bound interactions between tested proteins, these data suggested that interactions between Lpg0103 and eIF3-K are rather transient. We also tested AHNAK nucleoprotein, Homer protein homolog 3, insulin-like growth factor-binding protein 2, sperm-specific antigen 2, and thyroid hormone receptor-associated protein 3 for direct interactions with Lpg0103 using the Y2H assay. However, no yeast growth was observed on a selective medium for these proteins when co-expressed with Lpg0103 (data not shown). Thus, we focused on the characterization of the observed interactions between VipF and eIF3-K.

To test if interaction with eIF3-K interaction is shared among VipF orthologs, we performed a Y2H assay between eIF3-K and four representatives of this effector family, including Lha0223, Lfa0162, Lmic3006, and Llo3312, which share different percentages of primary sequence similarity with Lpg0103 (Fig. 2C). All four VipF orthologs demonstrated the ability to support yeast growth when co-expressed with the subunit K of human eIF3 complex (Fig. 2D). To further test VipF interactions with eIF3, we transfected HEK293T cells with a construct expressing Lpg0103 as a wild-type (WT) or as a catalytically inactive (Y263A) variant each fused to an N-terminal FLAG affinity tag. In line with our previous results, VipF constructs were co-precipitated with the eIF3 complex using an antibody specific to the scaffolding subunit of the eIF3 complex—eIF3-B (Fig. S8A). Furthermore, we performed immunofluorescence microscopy to determine the cellular localization of Lpg0103 in HEK293T cells. We observed that Lpg0103 is located throughout the cell; specifically, the cytoplasmic localization would allow this effector to directly interact with the eIF3 complex (Fig S8B). Based on these data, we postulated that *L. pneumophila* VipF can engage with the host eIF3 complex through direct interactions with the eIF3-K subunit, and this ability is shared by its homologs. The presence of all 13 eIF3 subunits in the VipF co-precipitation mix also suggested that the interaction between eIF3-K and VipF does not lead to this subunit’s dissociation from the eIF3 complex.

### Conserved hydrophobic residues in the interdomain cleft of VipF mediate interaction with the C-terminal tail of eIF3-K

The eIF3-K subunit is the smallest subunit of the eIF3 complex that has been structurally characterized both individually and as part of this complex (30, 32). This analysis revealed that the N-terminal portion of eIF3-K features a HEAT analogous motif and winged helix-like domain, followed by the so-called C-terminal tail (32). While this subunit is lacking in some yeast species, including *Saccharomyces cerevisiae*, it is conserved in higher eukaryotes as well as in multiple amoeba species that have been reported as susceptible hosts to *Legionella* infection (31–33).

**Fig 3:**
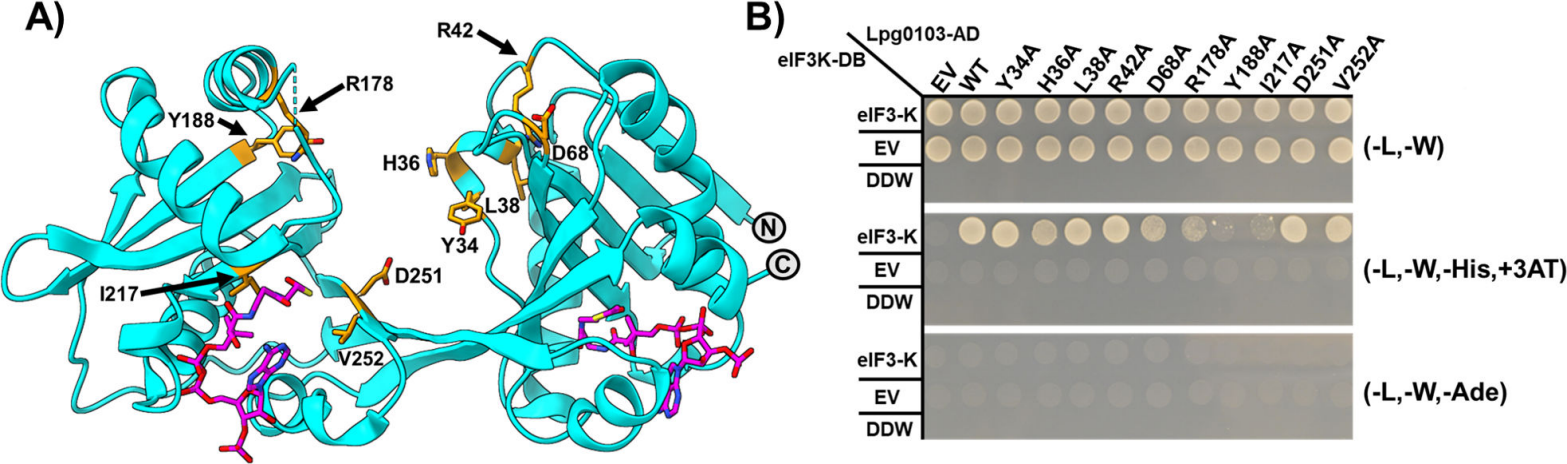
Mutational analysis of highly conserved residues that mediate interaction with human eIF3-K. (**A**) Mapping of highly conserved residues (orange) located in the interdomain cleft of the Lha0223 crystal structure. (**B**) Y2H experiment of the alanine substitution of highly conserved residues residing in the interdomain cleft of VipF. Double distilled water (DDW) was spotted on plates to ensure growth was not due to contamination. The Y2H experiment was performed in three independent experiments with similar results.

Since we identified that at least five VipF orthologs can form interaction with the human eIF3-K subunit, despite significant sequence variation, we hypothesized that the residues involved in this interaction must be conserved across these proteins. An amino acid conservation analysis using the Consurf Server (https://consurf.tau.ac.il/consurf_index.php) revealed several such residues co-localized to the GNAT interdomain cleft, which was visualized on the Lha0223-ligand complex crystal structure discussed above (Fig. 3A). With priority given to hydrophobic and charged residues, we individually targeted 10 conserved residues in Lpg0103 by site-directed mutagenesis and tested these variants for interactions with eIF3-K using Y2H assay (Fig. 3A). In the case of two substitutions, Y188A and I217A/S, we observed a dramatic loss of yeast growth phenotype, which suggests the disruption of VipF interaction with eIF3-K (Fig. 3B; Fig. S9A). Since these two conserved residues co-localized to the interdomain groove in the Lha0223 structure, this result confirmed the importance of this structural element for interactions with eIF3-K.

**Fig 4:**
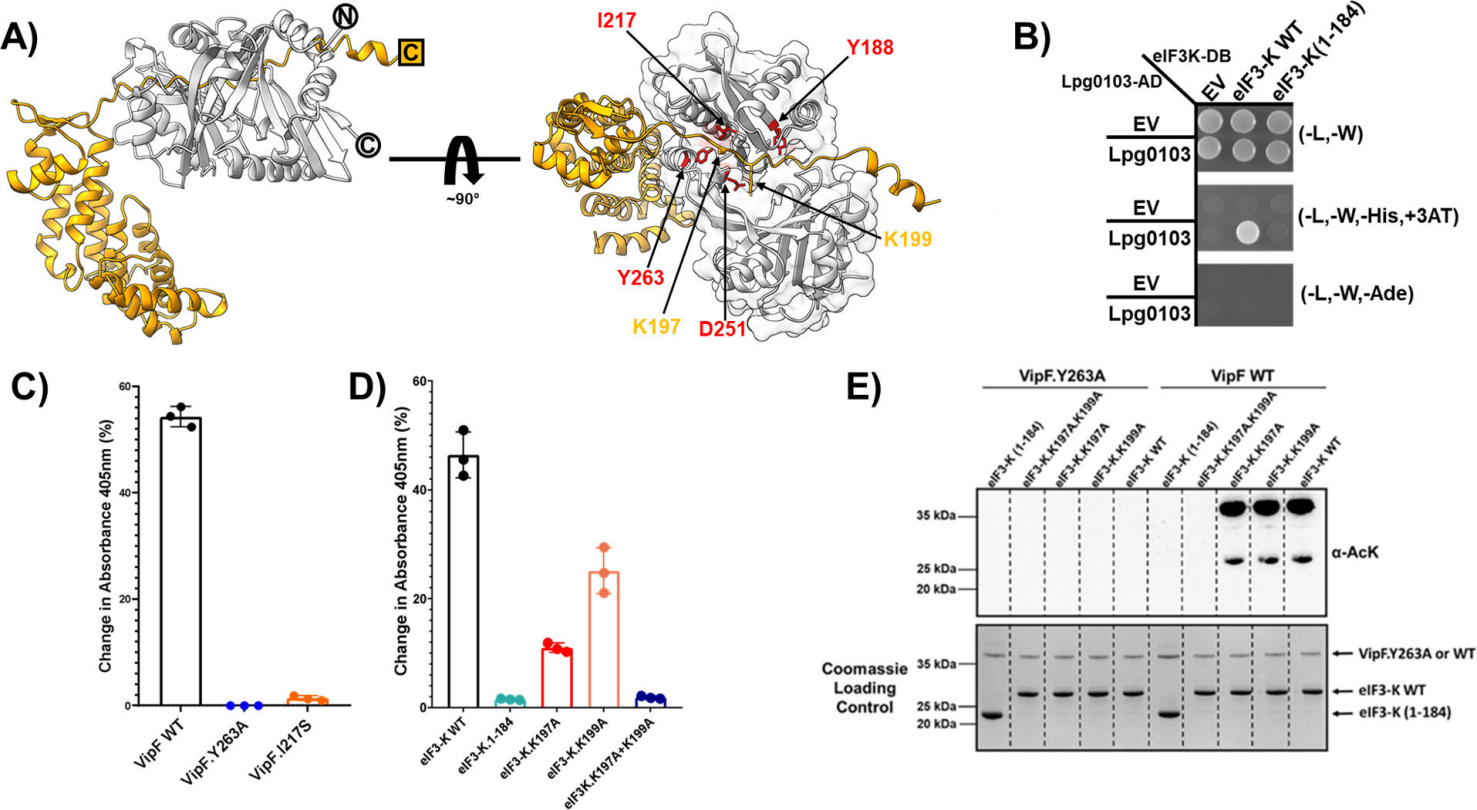
VipF interacts with the C-terminal tail of eIF3-K and is capable of acetylating eIF3-K *in vitro*. (**A**) AlphaFold2 model of the VipF (light gray)-eIF3-K (gold) complex. VipF residues essential for interaction and catalysis are highlighted in red. (**B**) Y2H analysis of pairwise interaction between VipF WT and either eIF3-K WT or eIF3-K (1-184). (**C**) 5´5´-Dithiobis(2-nitrobenzoic acid-based detection of the acetylation of eIF3-K WT with VipF WT, VipF Y263A, or VipF I217S. (**D**) Determination of the acetylation of two lysine residues present in the C-terminal tail of eIF3-K WT, eIF3-K (1-184), eIF3-K K197A, eIF3-K K199A, or eIF3-K K197A + K199A. (**E**) Western blot analysis of the acetylation of eIF3-K WT in the presence of VipF WT. For the samples with full-length eIF3-K, eIF3-K K197A, and eIF3-K K199A, we also observed an acetylated protein band corresponding to Lpg0103, which may be a result of self-catalysis promoted by the presence of eIF3-K. The amount of protein used for immunoblotting analysis is indicated by the Coomassie blue stain gel (bottom). The enzymatic assays and western blot experiments were performed in three independent experiments, rendering similar results.

Next, we modeled the interactions between Lpg0103 and eIF3-K using the AlphaFold2 server (https://colab.research.google.com/github/sokrypton/ColabFold). The most confident complex model predicted the interaction involving the interdomain groove of Lpg0103 and the C-terminal tail of eIF3-K (Fig. 4A; Fig. S10); furthermore, AlphaFold2 also produced similar models for VipF orthologs complexed with the eIF3-K subunit. Since the suggested interface on the Lpg0103 effector correlated with our mutagenesis analysis, we decided to validate the possible involvement of the C-terminal tail of eIF3-K in this interaction. The Y2H assay showed that the co-expression of Lpg0103 and the eIF3-K (1-184) variant lacking the C-terminal tail resulted in the loss of yeast growth phenotype on a selective medium (Fig. 4B; Fig. S11), thus supporting the suggested model of the Lpg0103-eIF3-K interaction. Furthermore, the position of the eIF3-K C-terminal tail in the interdomain cleft of VipF placed it in proximity to the active site of Lpg0103 with this region of eIF3-K featuring two lysine residues—K197 and K199—that are also broadly conserved in the protozoan hosts of *Legionella*.

Based on these observations, we postulated that eIF3-K’s C-terminal tail may represent the substrate of Lpg0103 catalytic activity. We investigated whether lysine residues on eIF3-K were modified by Lpg0103 using mass spectrometry. The analysis of the primary sequence coverage of eIF3-K when purified from Lpg0103-transfected HEK293T cells was over 70%; however, there was no coverage of the C-terminal tail in multiple mass spectrometry runs (Fig S6B). Thus, we reasoned that the peptide is likely not compatible for mass spectrometry-based detection. Alternatively, we first compared the *in vitro* activity of the wild-type Lpg0103 against full-length eIF3-K to that of the Y263A catalytically inactive or I217S binding-deficient variants. The former mutant of Lpg0103 demonstrated complete loss of activity against both small molecule and peptide generic substrates, while the latter one was incapacitated in eIF3-K interaction in Y2H assay (Fig. 4C; Fig. S9A and C). Both Lpg0103 Y263A and I217S mutants showed complete loss of activity against eIF3-K *in vitro* (Fig. 4C). Next, based on our model, we performed the *in vitro* acetyltransferase activity assay with Lpg0103 wild-type against full-length eIF3-K, eIF3-K lacking the C-terminal tail region [eIF3-K (1-184)] or eIF3-K mutants lacking individual (K197A and K199A) or both (K197A/K199A) lysine residues. Wild-type Lpg0103 demonstrated strong acetyltransferase activity against full-length eIF3-K *in vitro*, whereas the activity was almost completely lost in the case of eIF3-K (1-184) and K197A.K199A variants (Fig. 4D). We also observed a decrease in activity in the case of eIF3-K lacking only one of two lysine residues (Fig. 4D). Combined, these *in vitro* data were in line with our hypothesis that the two broadly conserved lysine residues located on the C-terminal tail are critical for VipF-mediated acetylation. To further test this, we performed a western blot analysis of Lpg0103 activity reaction samples containing eIF3-K, eIF3-K.K197A, eIF3-K.K199A, eIF3-K.K197A.K199A, or the eIF3-K (1-184) fragment using an acetyl lysine-specific antibody (Fig. 4E). Similar samples with catalytically inactive Lpg0103 Y263A variant were used as a control. In support of Lpg0103 catalytic activity against eIF3-K, we observed an acetyl lysine containing bands corresponding to eIF3-K in the case of the sample containing the full-length eIF3-K, eIF3-K.K197A, and eIF3-K.K199A, but not in case of the eIF3-K (1-184) fragment and eIF3-K.K197A.K199A (Fig. 4E). The lack of difference in observed acetylated signal between the wild-type and the single lysine mutants of eIF3-K was attributed to steric hindrance, thus not allowing for the antibodies to bind both acetylated lysine residues in the case of the wild-type eIF3-K.

### VipF activity suppresses protein translation *in vitro*

**Fig 5:**
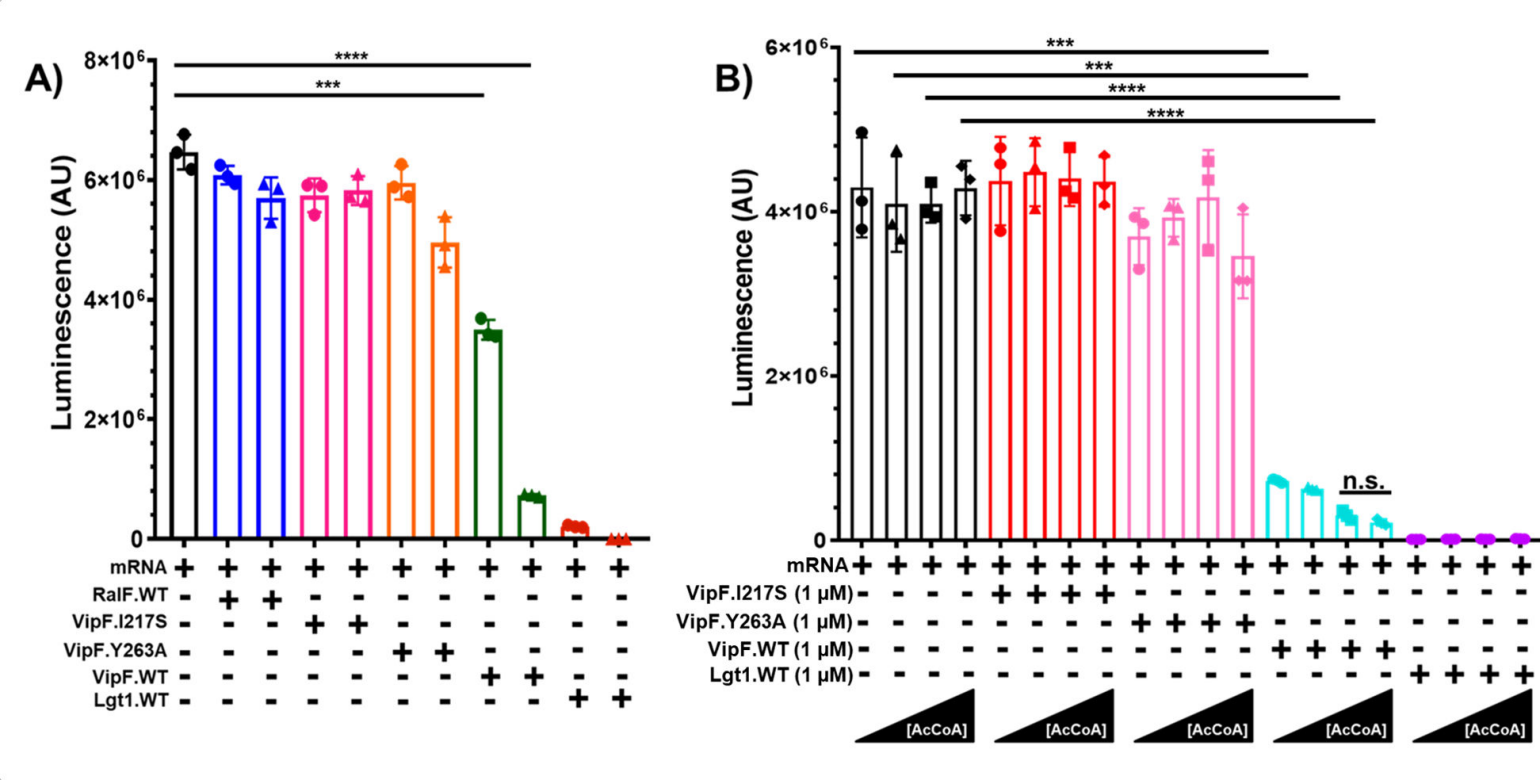
VipF dampens protein translation in a eukaryotic cell lysate. (**A**) Translation of luciferase mRNA in RRLs incubates with either RalF, Lgt1, VipF WT, VipF I217S, or VipF Y263A. Lysate was incubated with either 0.1 µM (circle symbol) or 1 µM (triangle symbol) of purified effector protein. (**B**) Translation of luciferase mRNA in RRLs incubated with either Lgt1, VipF WT, VipF I217S, or VipF Y263A. Lysates were incubated with 1 µM of purified effector protein. RRLs were also incubated in the absence (circle symbol) or presence of acetyl coenzyme A (AcCoA) (triangle symbol = 25 µM, square symbol = 50 µM, and diamond symbol = 75 µM). The data are the representation of three independent experiments that had similar results. The asterisks represent the statistical significance generated using the *t*-test (***, *P* < 0.001; ****, *P* < 0.0001; n.s., not significant).

Due to the essential role played by the eIF3 complex in eukaryotic protein translation, we investigated the potential role of the newly established VipF activity against its K subunit in this process. For this, we tested the effect of VipF/Lpg0103 in the *in vitro* protein translation assay that quantifies the translation of firefly luciferase mRNA in an *Oryctolagus cuniculus* (rabbit) reticulocyte cell lysate (RRLs). This assay has been previously used to characterize the activity of *L. pneumophila* effectors targeting protein translation, such as Lgt1, which is a glycosyltransferase that exclusively targets S53 on eukaryotic elongation factor 1A (eEF1A) (34). The glycosylation of eEF1A causes a drastic inhibition of protein synthesis *in vitro*. Relevant to the VipF function, the RRL contains a functional eIF3 complex with subunit K, which is almost identical to the human counterpart in amino acid composition (99.1% ID) and possesses the equivalents of both K197 and K199 in its C-terminal tail. In addition to the assay with Lgt1, which we used as a positive control, we also performed the same assay in the presence of another *L. pneumophila* effector, RalF, the function of which has been well characterized. RalF is a guanine nucleotide exchange factor that recruits human ADP-ribosylation factor 1 onto the LCV and then activates this host factor (35). Therefore, RalF was used as the negative control along with an assay containing no effector protein. In accordance with our enzymatic data, we observed a considerable inhibition of protein translation in the case of an assay containing Lpg0103.WT, but not the catalytically inactive Y263A mutant or protein binding-deficient I217S mutant (Fig. 5A). The decrease in protein translation observed for Lpg0103 was less dramatic than the one observed for Lgt1, suggesting that VipF acetylation of eIF3-K does not result in a complete loss of eIF3 function in protein translation. The Lpg0103 inhibition of the protein translation signal appears to be dependent on the concentration of acetyl-CoA co-substrate, which is in line with its acetyltransferase activity and is essential for the observed effect (Fig. 5B). Furthermore, between 50 µM and 75 µM, there was a saturation of acetyl-CoA that led to no statistically significant decrease in protein translation caused by Lpg0103.WT (Fig. 5B). Combined, our results establish that Lpg0103 is capable of dampening protein translation *in vitro* in accordance with its acetylation of subunit K of the eIF3 complex.

## DISCUSSION

The conservation of a small subset of Dot/Icm translocated effectors across *Legionella* spp. suggests a potentially important role in the intracellular life cycle of these bacteria. Part of these “core” effectors is the VipF family that is represented by Lpg0103 in *L. pneumophila*, and carries primary sequence features typical of GNAT acetyltransferases, but the function of VipF inside the host cell remained unknown. Due to the lack of tractable phenotype associated with *lpg0103* deletion in *L. pneumophila* (16), we undertook a comprehensive molecular characterization of representatives of this family using structural, *in vitro*, and model system approaches.

Expanding on previous structural studies, we determined crystal structures of the VipF ortholog, Lha0223, in complex with acetyl-CoA or CoA. In agreement with sequence prediction, the dual domain symmetry of Lha0223 confirms VipF and its orthologs as members of the tandem GNAT subfamily. Notably, VipF and its orthologs remain the only known instance of bacterial effectors belonging to this enzyme sub-group. Our assessment of the Lpg0103 and Lha0223 catalytic profiles using a general substrate screen showed both effectors having comparable activity against the same set of small molecules as well as peptide substrates. In line with this enzymatic data, the structural alignment of the Lha0223 structure with previously characterized GNAT enzymes indicated that this effector’s active site structural features are similar to those of members of the Rim GNAT acetyltransferase family, which modify bacterial ribosomal proteins (36).

In line with previously structurally characterized tandem GNAT enzymes, we observed dramatically different orientations of the acetyl-CoA substrate molecule in the binding site of the N- versus C-terminal domains of the Lha0223 structure. The binding site in Lha0223’s N-terminal domain consisted of hydrophobic residues lacking catalytical properties typical for the active site of GNAT enzymes. In contrast, our analysis of the binding pocket in the C-terminal domain of Lha0223 revealed conserved residues, Asp251 and Tyr263, positioned toward the co-substrate molecule in the manner typical of a catalytic dyad. Accordingly, the alanine substitutions of either residue in Lpg0103 or Lha0223 resulted in the complete abolishment of these effectors’ enzymatic activity. This contrasts with the non-essential role suggested for the latter residue by a previous report (23). The difference in observed effect of Tyr263 residue mutation may be attributed to variations in experimental procedures used in ours versus previous report. Specifically, the enzymatic data in previous report were obtained using higher amounts of enzyme and over significantly longer assay time. Furthermore, to the best of our knowledge, tandem GNAT enzymes have not been experimentally validated to rely on residues outside of the GNAT fold, as previously described for the role of Glu129 in Lpg0103. A possibility is that Glu129 is involved in the stabilization of the substrate in the interdomain cleft but may not be required in the transfer of the acetyl group onto the substrate. The role of the non-catalytic domain in VipF effectors along with similar domains in the other representatives of tandem GNAT remains elusive. Its ability to bind the co-substrate was suggested to play an allosteric role; however, such function in the case of VipF would require further investigation (23). Importantly, the N-terminal lobe of VipF contributes residues to the central interdomain cleft which we identified to be involved in the binding of VipF’s protein substrate.

Our search for a potential host interactor of Lpg0103 using mass spectrometry-assisted affinity purification pointed to the eIF3 complex, the largest and most complex protein complex involved in translation that is found in all eukaryotic species from humans to protists (30–33). Lpg0103 consistently co-precipitated with all 13 subunits of human eIF3 as well as with several host factors known to be associated with this complex. The eIF3 complex interacts with other translational factors, such as eIF1 and eIF4, and is involved in the initiation step of protein translation, including the recruitment of ribosomes to the translational machinery, guiding of the 43S complex to mRNA, and scanning of mRNA (30). Using Y2H assays, we identified subunit K of the eIF3 to be directly interacting with Lpg0103, along with four other VipF homologs. These data identify eIF3-K as a possible common target for VipF effectors in eukaryotic hosts. Using the combination of molecular modeling and Y2H analysis, we determined that the Lpg0103 effector is binding the C-terminal tail portion of eIF3-K and acetylates two lysine residues, K197 and K199, *in vitro*. Along with other eukaryotic species, these residues are broadly conserved in eIF3-K of protist species representing the natural hosts of *L. pneumophila*. Future studies will involve the identification of the acetylation of the eIF3-K subunit and other potential substrates of Lpg0103 from infected amoebae and differentiated U937 cells. Furthermore, the modification of multiple lysine residues within the vicinity of each other on the host target has been adopted by other bacterial effectors, such as IcsB from *Shigella flexneri* that fatty acylates multiple lysine residues on the C-terminal polybasic tail of multiple members of the Ras, Rho, and Ras small GTPase families (37). The biological significance of this modification for *S. flexneri* infection has not been elucidated.

The hijacking of host protein translation during *L. pneumophila* infection has been exemplified by the characterization of the functions of Lgt1-3, SidI, and SidL effectors (34, 38–40). Similarly, the VipF catalytic function results in the suppression of eukaryotic protein translation *in vitro* which we suggest is triggered by modification of the eIF3-K subunit. It has been suggested that halting protein synthesis during the initiation and elongation steps would lead to the degradation of nascent peptide chains that can fuel the nutritional requirements of infecting bacteria (40, 41). However, the functional consequences of VipF and other protein translation regulating *L. pneumophila* effectors during infection require further exploration. Notably, the genetic knockdown of the eIF3-K encoding gene does not have a deleterious effect on human cell viability and does not affect the expression of the remaining eIF3 subunits (42). In addition to being part of eIF3, eIF3-K has been shown to engage in several biological interactions outside of this complex (43–47). These include associations with cell cycle regulatory factor cyclin D3 and 5-HT_7_ class receptors involved in immune regulation and inflammatory cascades in alveolar macrophages (43–45). Furthermore, eIF3-K has been shown to be involved in regulating cellular responses to endoplasmic reticulum stress (46). Recently, eIF3-K was shown to act as an inhibitor of the Nuclear factor kappa B (NF-κB) pathway through interactions with MyD88 that aid in the evasion of the innate immune response in teleost fish cells infected with *Vibrio harveyi* (47). These examples provide several avenues for VipF to affect cell physiology and innate immune system activation via the modification of eIF3-K which can be independent of the eIF3 complex.

*Legionella’s* effector arsenal allows for the efficient colonization of a wide range of hosts ranging from unicellular amoeba, ciliated protists, and human alveolar macrophages. Universally conserved across this genus, the “core” effectors may contribute to this generalist nature of *Legionella’s* intracellular pathogenesis by targeting elements of eukaryotic physiology that are highly conserved across the eukaryotic kingdom. Supporting this hypothesis, our study into the molecular function of “core” effector VipF establishes it as a tandem GNAT acetyltransferase that catalyzes the acetylation on two lysine residues present on the C-terminal portion of a specific subunit in the host eIF3 complex. These new data significantly advance our understanding of eukaryotic machinery targeted by the core portion of the *Legionella* species effector arsenal.

## MATERIALS AND METHODS

### DNA manipulations

Phusion DNA polymerase (New England Biolabs, Canada) was used to perform PCR reactions to introduce ligation-independent cloning (Lic) sequences, *attB* flank regions, and restriction sites at 5´ and 3´ of amplified fragments for Lic, Gateway, and restriction enzyme-mediated cloning, respectively. Point mutations were introduced by PCR amplifying plasmids harboring the insert of interest using primers encoding for the mutation and self-ligation of the PCR product. Constructs were validated by sequencing and plasmid DNA was isolated using a Presto Mini Plasmid Kit and Geneaid Plasmid Maxi Kit (Geneaid, Taiwan).

### Protein expression and purification

The expression plasmid pMCSG68SBPTEV was used for recombinant protein expression of all proteins and their respective mutants in this study. This overexpression plasmid has an N-terminal 6xHis-Streptavidin-Binding Peptide (SBP). Proteins were overexpressed in *E. coli* BL21-Gold (DE3) (Stratagene). Cultures were grown at 37°C, 175 RPM in Lysogeny Broth (LB) medium supplemented with ampicillin (100 ug/mL) to OD_600 nm_ of 0.6–0.8, at which point protein expression was induced with the addition of 0.5 mM isopropyl-β-D-thiogalactopyranoside, BioShop) overnight at 16°C. Cells were harvested by centrifugation (7,000 × *g* for 1 hour at 4°C) and then resuspended in binding buffer [300 mM NaCl, 50 mM HEPES pH 7.5, 5 mM imidazole, 5% (vol/vol) glycerol] in the presence of 0.5 mM phenylmethylsulfonyl fluoride, then lysed by sonication on ice (65% amplitude, 5 s on, 10 sec off, for 15 min). The soluble protein fraction was obtained by ultracentrifugation (20,000 × *g* for 1 hour at 4°C). The soluble protein fraction was then incubated with nickel-nitrilotriacetic acid agarose beads (Ni-NTA, Bio-Rad) for 1 hour at 4°C. The protein was washed with a wash buffer [300 mM NaCl, 50 mM HEPES pH 7.5, 30 mM imidazole, and 5% (vol/vol) glycerol], then eluted with elution buffer [300 mM NaCl, 50 mM HEPES pH 7.5, 300 mM imidazole, 5% (vol/vol) glycerol]. 6xHis-SBP-tagged proteins used for affinity purification mass spectrometry were dialyzed overnight in dialysis buffer [300 mM NaCl, 30 mM HEPES pH 7.5, 5% (vol/vol) glycerol, and 1 mM tris(2-carboxyethyl)phosphine], then concentrated using an Amicon concentrator (Millipore). However, in the case of *in vitro* enzymatic assays, purified tagged proteins were dialyzed overnight in dialysis buffer in the presence of TEV protease. The 6xHis-SBP tag and TEV proteases were separated by binding onto a Ni-NTA column where the flow-through was collected to obtain the cleaved protein of interest. Proteins were subjected to size exclusion chromatography for further purification using a Superdex 200 Increase 10/300 column (Cytiva) equilibrated with dialysis buffer. The purity of the collected protein fractions was analyzed by SDS-polyacrylamide gel electrophoresis. Pure protein samples were concentrated, followed by flash freezing in LN2, and then stored at −80°C.

### *In vitro* activity screens

Reagent-grade small molecules and the human histone H3 peptide (residues 1–21) synthesized were purchased from Sigma-Aldrich. Activity screens were performed against general substrates following a previously described procedure (27). Briefly, 1 µg of purified effector was added to a reaction mixture containing 50 mM Tris-HCl pH 8.0, 0.5 mM acetyl-CoA, and 2.5 mM substrate to 50 µL final volume and incubated for 10 min at 33°C. The reaction was quenched with 200 µL probe solution [6 M guanidine HCl, 0.2 mM 5´5´-dithiobis(2-nitrobenzoic acid)] and allowed to develop for 10 min at room temperature. Absorbance at 412 nm was measured using a Wallac 1420 Victor2 Microplate reader (PerkinElmer). Assays were performed in triplicate with controls of no substrate and no enzyme to detect spontaneous hydrolysis of acetyl-CoA, as well as blank of the substrate with acetyl-CoA in the absence of enzyme. Lastly, a blank of the enzyme with acetyl-CoA in the absence of substrate was also used as a control. All reactions were set by hand with multi-channel pipettors. The average readout for controls was calculated as a percent change in absorbance at 412 nm relative to the highest background control.

### *In vitro* protein translation assay

Luciferase-based protein translation assays were performed using a Promega Flexi rabbit reticulocyte lysate system (L4540). The experiment was performed following the protocol of the manufacturer. In brief summary, tubes were aliquoted with a master mix of the lysate, followed by the addition of either Lgt1.WT, RalF.WT, VipF.WT, VipF.I217S, or VipF.Y263A. Each protein had a final concentration of 0.1 µm or 1 µm, which was indicated for each experiment. The protein-lysate reaction mixtures were mixed and then centrifuged, followed by a 90-min incubation at 30°C. The bioluminescent signal of the translated luciferase mRNA was detected and quantified using a PerkinElmer Victor NIVO plate reader.

### Crystallization

Prior to crystallization, Lha0223 was dialyzed against 300 mM potassium chloride and 10 mM HEPES (pH 7.5) and then concentrated up to 20 mg/mL using an Amicon concentrator (Millipore). Crystallization of Lha0223 was performed at room temperature using the vapor diffusion method in sitting drops with 0.6 µL protein or protein:ligand mixture plus 0.6 µL reservoir solution, using TTP Labtech Mosquito protein crystallization robot. VipF-CoA complex was crystallized by combining SeMet-derivative Lha0223 protein with a reservoir solution composed of 1.1 M ammonium tartrate, 0.1 M sodium acetate pH 4.6, and 50 mM Tris pH 9.0. VipF carried CoA from purification. VipF-acetyl-CoA complex was crystallized using native Lha0223 and incubated for 60 min with 5 mM acetyl-CoA plus reservoir solution composed of 20% (wt/vol) Polyethylene glycol (PEG) 3350 and 0.2 M calcium chloride. Prior to data collection, crystals of both complexes were cryoprotected by transferring them to Para-tone oil and flash-frozen in liquid nitrogen.

### Structure determination and refinement

Diffraction data were collected at 100 K at beamline 21-ID-G at the Structural Biology Center, Advanced Photon Source at Argonne National Laboratory at a wavelength 0.9794. All diffraction data were reduced with HKL-3000. Data were indexed with XDS, and integration and scaling were performed in CCP4. Selenomethionine-substituted Lha0223 (VipF•CoA) structure was solved by single-wavelength anomalous dispersion phasing using PHENIX.solve and the model was constructed using PHENIX.autobuild. The structure of native Lha0223 in complex with acetyl-CoA (VipF•acetyl-CoA) was determined by molecular replacement using VipF•CoA as a search model with PHENIX.phaser. Both structures were refined using PHENIX.refine and Coot. Geometries were verified with PHENIX.refine and the wwPDB validation server. Coordinates have been deposited in the RCSB Protein Data Bank under accession codes 6WQB and 6WQC for VipF•acetyl-CoA and VipF•CoA structures, respectively.

### *In situ* affinity purification coupled with mass spectrometry

Effector interactions with host proteins were assayed by affinity purification coupled with mass spectrometry. Streptavidin magnetic beads were used in combination with tube racks affixed with neodymium magnets to aid purification. The host cell lysate was prepared from 5 × 10^7^ U937 cells per pulldown repeat through freeze-thaw lysis in Affinity purification coupled with mass spectrometry (AP-MS) buffer [50 mM HEPES pH 8.0, 150 mM NaCl, 0.2% (vol/vol) NP-40, 1 mM DTT, 0.5 mM EDTA, and cOmplete Mini (Roche) protease inhibitor]. Following clarification of the soluble cell fraction from the total lysate by centrifugation, endogenous biotin was pre-cleared using streptavidin magnetic beads (NEB) equilibrated in AP buffer for 60 min at 4°C with gentle rotation.

Approximately 200 ng of purified SBP-tagged constructs were incubated with 50 µL streptavidin-coupled magnetic sepharose beads (Life Technologies) for 1 hour at 4°C to generate bait-bead complexes used for each pulldown repeat. Bait-bead complexes were incubated with clarified lysate for 180 min at 4°C and purified by magnetic separation in combination with three AP buffer washes before being transferred into 50 mM ammonium bicarbonate (AMBIC). The bait-target complexes were eluted off the beads by incubation with 2.5 mM biotin in 50 mM AMBIC for 15 min at room temperature.

Sequencing grade trypsin (Promega) was used to digest effector-interactor complexes overnight at 37°C and halted by the addition of 0.5% trichloroacetic acid. Tryptic peptides were purified using C18 OMIX tips (Agilent) and eluted into 0.1% formic acid in acetonitrile and afterward dried by vacuum centrifugation before resuspension in 0.1% formic acid in Liquid chromatography–mass spectrometry (LC-MS) grade water.

### Yeast two-hybrid

Yeast two-hybrid experiments were performed as described (46). Briefly, bait and prey proteins were fused to either the GAL4 transcriptional activation domain (AD) or the DNA-binding domain (DB) using pDEST-AD-ccdB and pDEST-DB-ccdB constitutively expressing Gateway destination plasmids co-transformed into *S. cerevisiae* strain Y8800 (MATa leu2-3,112 trp1-901 his3-200 ura3-52 gal4D gal80D GAL2-ADE2 LYS2::GAL1-HIS3 MET2::GAL7-lacZ cyh2R). Transformants were grown on synthetic defined (SD) media containing 2% glucose as a carbon source, omitting tryptophan and leucine for the selection of bait and prey plasmids. Three independent colonies from fresh plates were grown overnight in SD liquid media, collected, washed with ultra pure water, and spotted onto plates either lacking leucine and tryptophan for selection of bait and prey plasmids, lacking leucine, tryptophan, and histidine for selection of HIS3 reporter, and finally lacking leucine, tryptophan, and adenine for selection of ADE2 reporter. Plates were imaged continuously over 72 hours following spotting.

### Mammalian cell culture

Human monocyte-like U937 cells (ATCC CRL-1593.2) were obtained for the preparation of human proteome samples to be used for *in situ* affinity purifications. Cells were cultured in Roswell Park Memorial Institute (RPMI)-1640 medium supplemented with 2 mM L-glutamine (Thermo Scientific), 10% fetal bovine serum (FBS; VWR) and 1% penicillin/streptomycin (Pen/Strep) solution (Sigma-Aldrich) maintained at 37°C and 5% CO_2_. U937 cells were grown in suspension in T-175 tissue culture (TC) flasks (Sarstedt AG & Co. KG) until 90% confluent, then they were harvested by centrifugation, flash frozen in liquid nitrogen, and stored at −80°C in 5 × 10^7^ cell aliquots.

HEK293T/17 SF cells (ACS-4500) were obtained for transfections. Cells were cultured in Dulbecco’s Modified Eagle Medium (DMEM) supplemented with 10% FBS and 1% Pen/Strep and maintained at 37°C and 5% CO_2_. For routine passaging, adherent cells were detached from the culture vessel with the addition of 0.25% trypsin-EDTA warmed to room temperature for 5 min, and neutralized by the addition of complete medium once detached. Cells used for assays were maintained in DMEM with 10% FBS lacking Pen/Strep and visually monitored for contamination. For assay seeding, trypsinized cells were counted, collected in a conical tube, gently pelleted 130 × *g* for 5 min at room temperature, and resuspended at the appropriate concentration.

Mammalian expression constructs fused to an N-terminal FLAG tag used for transient transfections were generated using Lpg0103 cloned into Gateway-compatible expression vector pDEST-pcDNA5-N-FLAG. Transient transfections were performed using Lipofectamine 3000 reagent (Life Technologies). Prior to transfection, HEK293T cells were plated in advance to be 70% confluent at the time of transfection in antibiotic-free complete DMEM. For imaging experiments, HEK293T cells were plated to be 30% confluent at the time of transfection. DNA-lipofectamine complexes were mixed in Opti-MEM and added to the cells as specified by the manufacturer’s protocol. The fresh antibiotic-free medium was added 6 hours post-transfection and cells were maintained for 30–36 hours to allow for protein expression.

### Immunofluorescence

For immunofluorescence experiments, HEK293T cells were grown on poly-L-lysine-coated coverslips (Corning; 354086) in the wells of a six-well TC-treated culture dish (Thermo Scientific; 140675) in antibiotic-free complete medium to 30% confluency prior to transfection. Each well was transfected with 2.5 µg of indicated plasmid construct mixed with Lipofectamine 3000 reagent. After 36 hours, media was aspirated, and cells were fixed with warmed 4% (vol/vol) paraformaldehyde in Phosphate-buffered saline (PBS) for 15 min at room temperature. Following fixation, the cells were rinsed three times with PBS for 5 min at room temperature, shaking. HEK293T cells were permeabilized with 0.3% (vol/vol) Triton X-100 in PBS for 5 min at room temperature. The cells were washed three times as before, then blocked with 3% (wt/vol) bovine serum albumin (BSA) in PBS for 30 min at room temperature. All subsequent steps were performed in the dark.

Primary antibodies were used at 2 µg/mL diluted in 3% BSA-PBS and incubated overnight at 4°C, shaking. Mouse monoclonal antibody to FLAG M2 epitope (Sigma Aldrich; F1804) was used to label 3xFLAG fusion proteins, while rabbit anti-eIF3-B (Bethyl; A301-761A) was used to label endogenous eIF3-B. Following three washes with PBS, coverslips were incubated with secondary antibodies prepared according to manufacturer instructions for 60 min. Donkey anti-rabbit conjugated to Alexa Fluor 488 (Abcam; ab150073) was used to fluorescently label eIF3-B, while donkey anti-mouse conjugated to Alexa Fluor 647 (Abcam; ab150107) was used to label 3xFLAG-tagged constructs. For localization requiring nuclear staining, 1 µg/mL of 4´,6-diamidino-2-phenylindole dihydrochloride in PBS was incubated with the coverslips for 1 min before a final set of washes was carried out. The coverslips were mounted with ProLong Gold Antifade reagent (Life Technologies; P10144) and allowed to be set overnight at 4°C before being sealed. Microscopy was performed using a Leica DMI4000B wide-field fluorescent microscope with a 60× oil-immersion lens and processed using Fiji (48).

### Immunoprecipitations

Coimmunoprecipitation experiments were performed as previously described (49). Briefly, 2 × 106 HEK293T/17 SF cells were seeded onto 100 mm TC-treated culture dishes in an antibiotic-free complete medium. Transfections were performed using Lipofectamine 3000 according to the manufacturer’s protocol. Cells were harvested 30 hours post-transfection and lysed by freeze-thaw in TNN buffer [50 mM Tris pH 7.5, 150 mM NaCl, 0.1% (vol/vol) Nonidet P-40, 1 mM EDTA, 1 mM DTT with Roche cOmplete mini protease inhibitor]. The soluble fraction was obtained by centrifugation and incubated with Protein A magnetic agarose beads (Cytiva, 28-9440-06) in the presence of 2 µg rabbit anti-eIF3-B antibody overnight at 4°C. The beads were magnetically separated, resuspended into 50 µL 3× Laemmli sample buffer, and boiled for 5 min. Reciprocal pulldown of FLAG-tag effector was performed using anti-FLAG magnetic agarose beads conjugated to anti-FLAG mouse monoclonal antibody (Sigma, M8823). Clarified lysate from transfected HEK293T/17 SF cells was prepared as before and incubated with anti-FLAG beads for 2 hours. The beads were magnetically separated, and samples were spiked with 5 µg of 3xFLAG peptide (APExBIO, A6001) to competitively elute from the beads. The elutions were boiled in 3× Laemmli sample buffer for 5 min. Immunoprecipitated proteins were resolved on SDS-PAGE gels and visualized by western blot.

### Western blot

Proteins were separated by electrophoresis in 12% SDS-PAGE gels and transferred to a nitrocellulose membrane. FLAG fusion proteins were detected with mouse anti-FLAG (Sigma, F1804) and anti-mouse IgG:HRP. eIF3-B and eIF3-K were detected with rabbit anti-eIF3-B (Bethyl, A301-760A), rabbit anti-eIF3-K (Abcam, ab85968), and anti-rabbit IgG:HRP. GAL4 fusion proteins were detected with anti-GAL4 (residues 1–147) (Abcam, ab135398), anti-GAL4 (residues 768–881) (Abcam, ab135397), and anti-mouse IgG:HRP. HRP-conjugated secondary antibodies were used for chemiluminescent exposures with Immobilon HRP Substrate (Millipore, Canada) and detected by imaging with a ChemiDoC MP (Bio-Rad, USA).

For the acetylation western blots, the *in vitro* acetylation assays were performed in a 50 µL reaction mixture as previously described (50). Briefly, each reaction mixture contained 0.5 µg of VipF WT or VipF Y263A, 33 µg of eIF3-K WT, eIF3-K.K197A, eIF3-K.K199A, eIF3-K.K197A.K199A, or eIF3-K (1-184), and 25 µM acetyl-CoA in a reaction buffer composed of 25 mM Tris-HCl pH 8.0, 200 mM NaCl, and 5% glycerol. These acetylation reactions were incubated for 1 hour at 33°C. After the reaction was completed, 10 µL of each sample was loaded onto a 12% SDS-PAGE gel for western blot analysis. Acetylated lysine was detected using mouse anti-acetyl lysine (Cell Signaling Technology, # 9681S).

## Data Availability

The data generated in this study are available in Supplementary Data I; RCSB Protein Data Bank (crystal structures) under accession codes 6WQB and 6WQC.

## SUPPLEMENTAL MATERIAL

Supplemental Figures mbio.03221-23-s0001.pdf Figures S1 to S11.

Supplemental Tables mbio.03221-23-s0002.docx Tables S1 and S2.

ASM does not own the copyrights to Supplemental Material that may be linked to, or accessed through, an article. The authors have granted ASM a non-exclusive, world-wide license to publish the Supplemental Material files. Please contact the corresponding author directly for reuse.
