# Supplementary material for: An acetyltransferase effector conserved across Legionella species targets the eukaryotic eIF3 complex to modulate protein translation
Source: mBio. 2024 Feb 9;15(3):e03221-23. doi: 10.1128/mbio.03221-23 (PMC10936415; doi:10.1128/mbio.03221-23)
Supplement: Supplemental Figures — Figures S1 to S11. [file mbio.03221-23-s0001.pdf]

Supplemental Information Figures

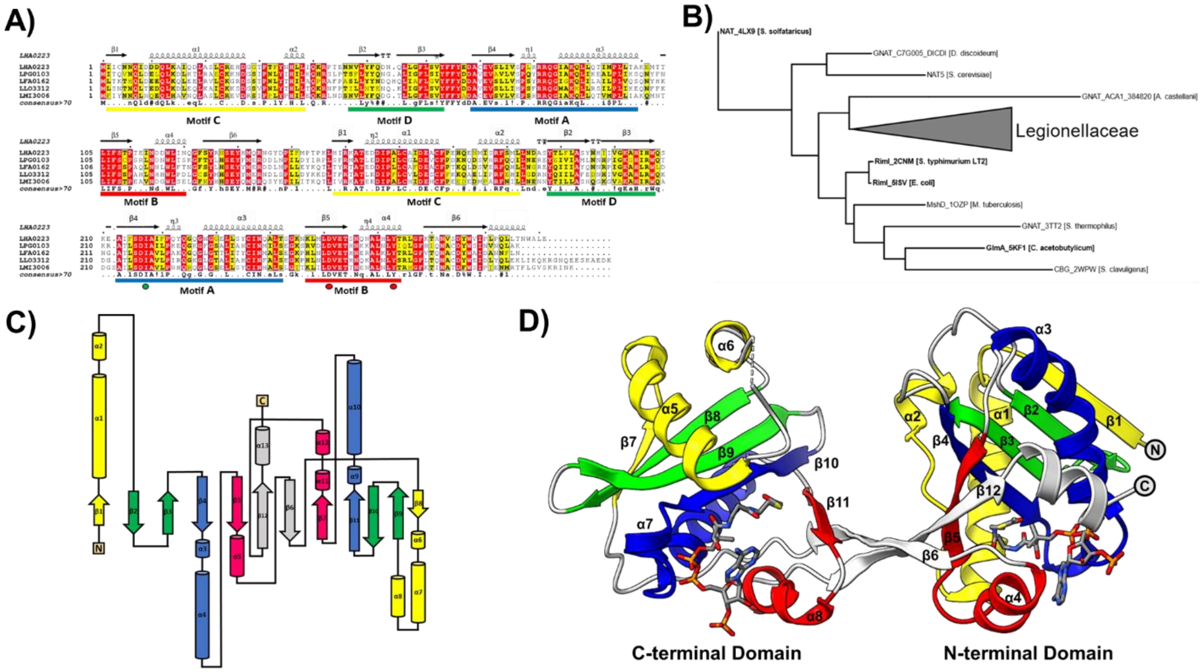

Supplemental Figure 1. Comparison of VipF ortholog amino acid sequences. (A) A multiple amino acid sequence alignments of VipF orthologs from species of *Legionella.* Residues highlighted in red are conserved in all VipF orthologs while yellow residues are partially conserved. (B) Maximum-likelihood tree representation of the VipF ortholog multiple sequence alignment in (A) and GNAT homologs from prokaryotes and single-celled eukaryotes. The tree was generated with 1000 bootstraps. IQ-TREE was the program used to generate the tree and ITOL was used to visualize. (C) Secondary structure topology of Lha0223. The secondary structure features corresponding to conserved motifs are colored yellow, green, blue, and red, respectively. The blue and red colored motifs form the acetyl-CoA binding site and catalytic region, which is conserved among GNAT enzymes. The yellow and green motifs are suggested to be involved in substrate recognition. (D) Representation of GNAT conserved motifs from (C) on the Lha0223 structure.

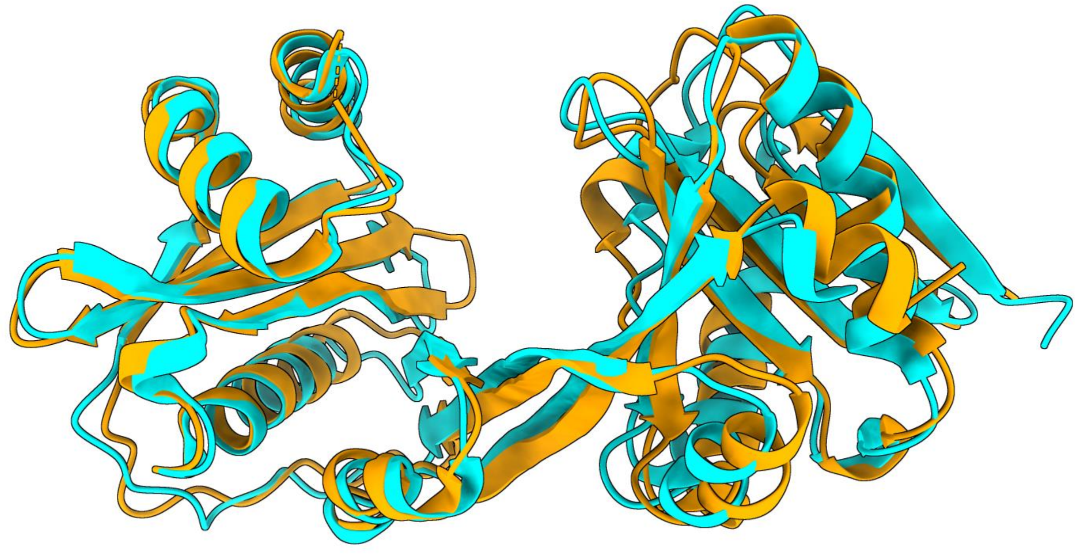

Supplemental Figure 2. Cartoon representation of the superimposition of the Lpg0103 crystal structure (cyan, PDB 7WX5) onto the Lha0223 crystal structure (orange, PDB 6WQB).

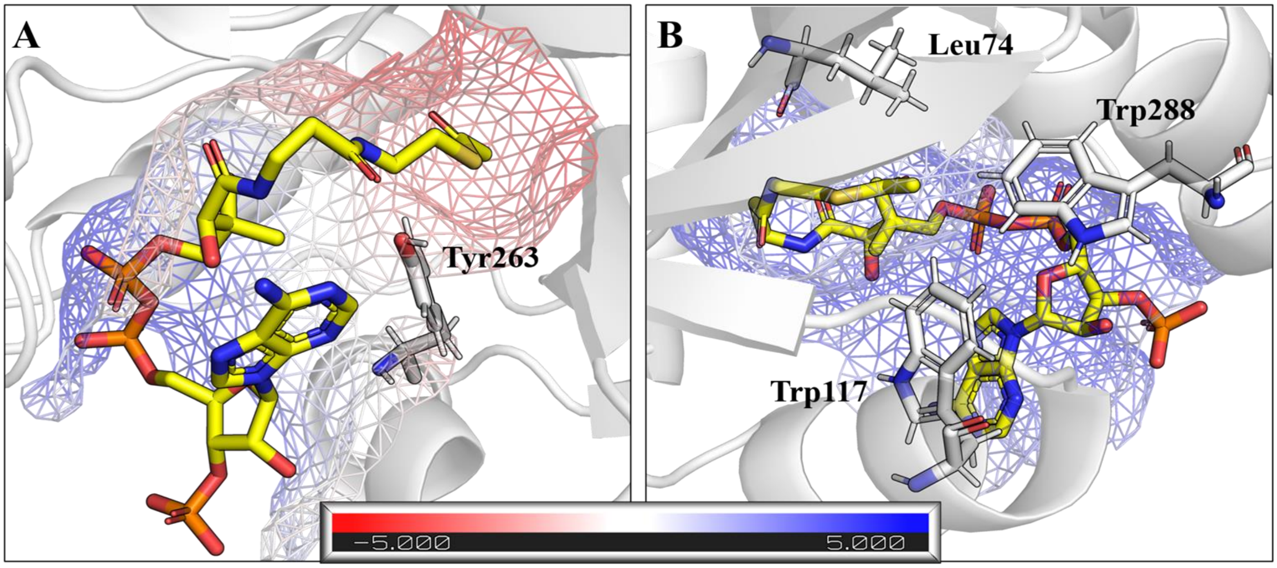

Supplemental Figure 3. Structural differences support the C-terminal GNAT domain as the site of catalysis of Lha0223. (A) The conformation of acetyl-CoA in the C-terminal GNAT domain lies in planar orientation to the solvent-exposed, negatively charged oxyanion channel (red mesh) of the central cleft in close proximity to a conserved Tyr263. (B) The N-terminal acetyl-CoA is enclosed by several hydrophobic sidechains in a closed hydrophobic pocket (blue mesh).

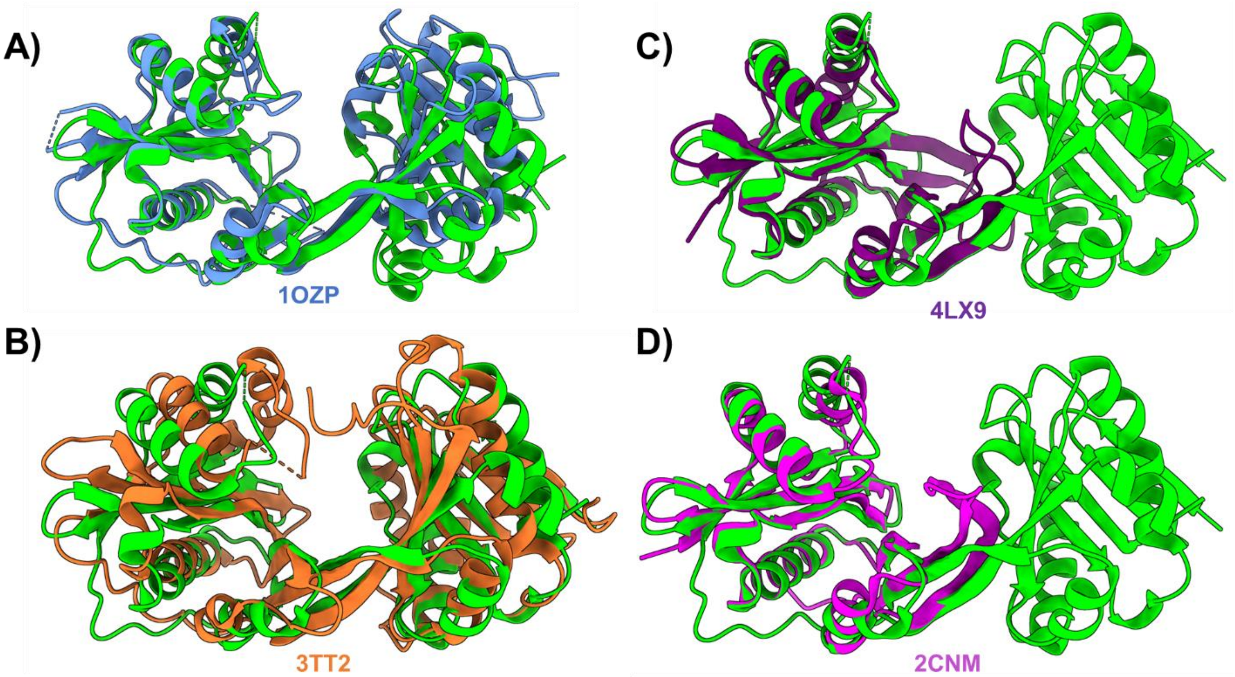

Supplemental Figure 4. Structural alignment of Lha0223 with proteins that adopt an overall GNAT fold. (A) Structures 1-4 are the most structurally similar proteins identified by the Dali server. The PDB codes for each structurally similar protein are shown under the alignment. Each structure was superimposed onto the cartoon representation of Lha0223 (lime green).

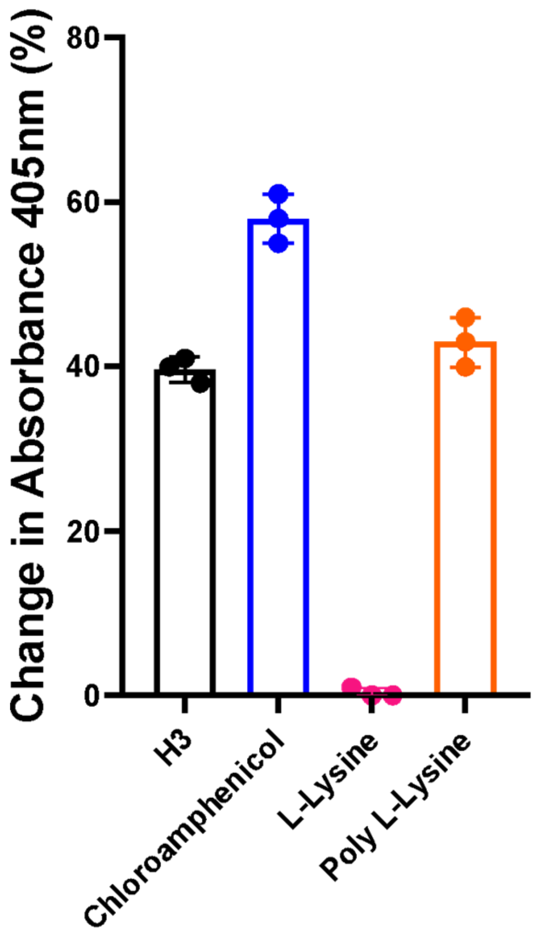

Supplemental Figure 5. Similar to Lha0223, Lpg0103 also has a comparable biochemical activity against small molecules and peptide substrates. The experiments were performed in triplicate with similar results.

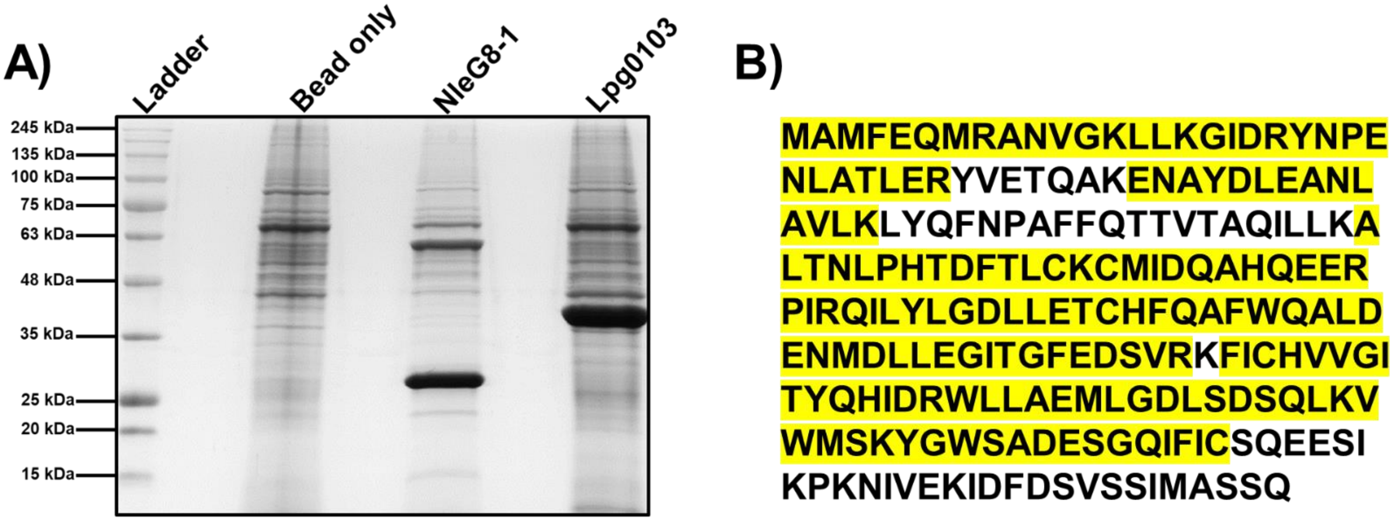

Supplemental Figure 6. (A) Coomassie blue stained gel of the bead only (negative control), NleG8-1 (positive control), and Lpg0103 AP-MS samples. (B) Primary sequence coverage (yellow highlight, 74.3%) of the eIF3-K subunit from mass spectrometry experiments to identify the acetylation of lysine residues by Lpg0103.

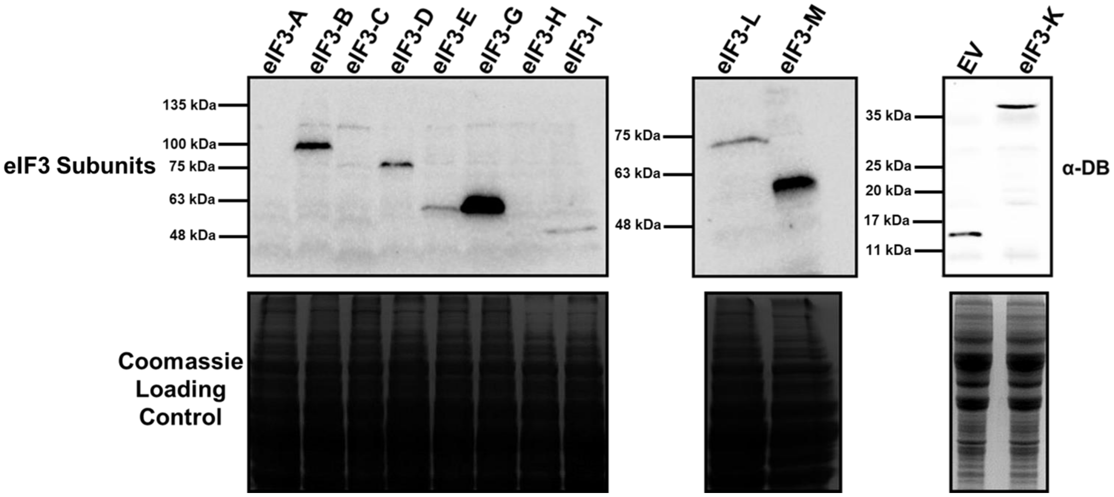

Supplemental Figure 7. Western Blot analysis and loading control of eIF3 subunits to assess the presence of human protein expression in the *S. cerevisiae* Y880 strain used for testing one-on-one interactions with Lpg0103 via Y2H in Figure 2B. Proteins were fused to an N-terminal Gal4 DNA binding domain and were detected using the anti-Gal4 DNA binding domain.

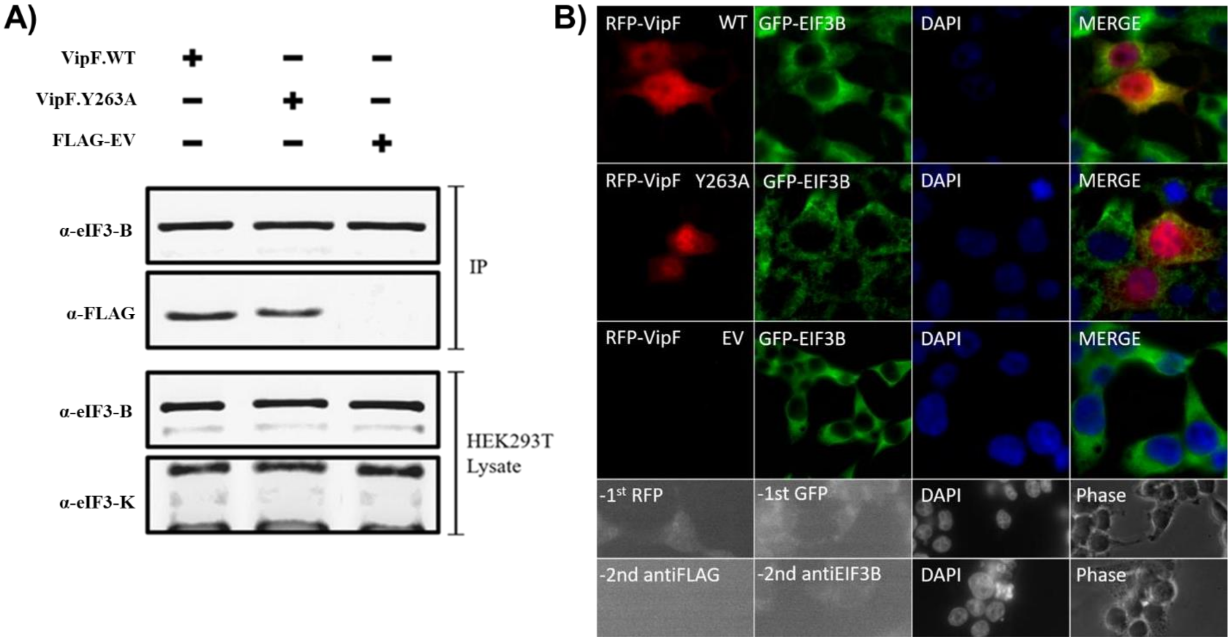

Supplemental Figure S8. Co-immunoprecipitation of human eIF3 complex and eIF3-K and cellular localization of VipF in HEK293T cells. (A) Co-immunoprecipitation western blot of either full-length Flag-tagged VipF.WT, VipF.Y263A, or a Flag-tag only that were transfected in HEK293T cells. Lysate of transfected cells was incubated with anti-eIF3-B and protein A magnetic agarose beads to purify VipF, or VipF Y263A, bound to the eIF3 complex. The bottom panel indicates the protein levels of eIF3-B and eIF3-K in the total HEK293T lysate. These data were performed in three independent experiments with similar results. (B) Immunofluorescent labeling of Flag-tagged constructs of wild-type VipF, the catalytic mutant Y263A, and 3xFLAG-empty vector were expressed in HEK293T cells and detected by anti-FLAG immunofluorescence (Alexa Fluor 647, red), anti-eIF3B (Alexa Fluro 488, green), and counterstained for nuclei (DAPI, blue).

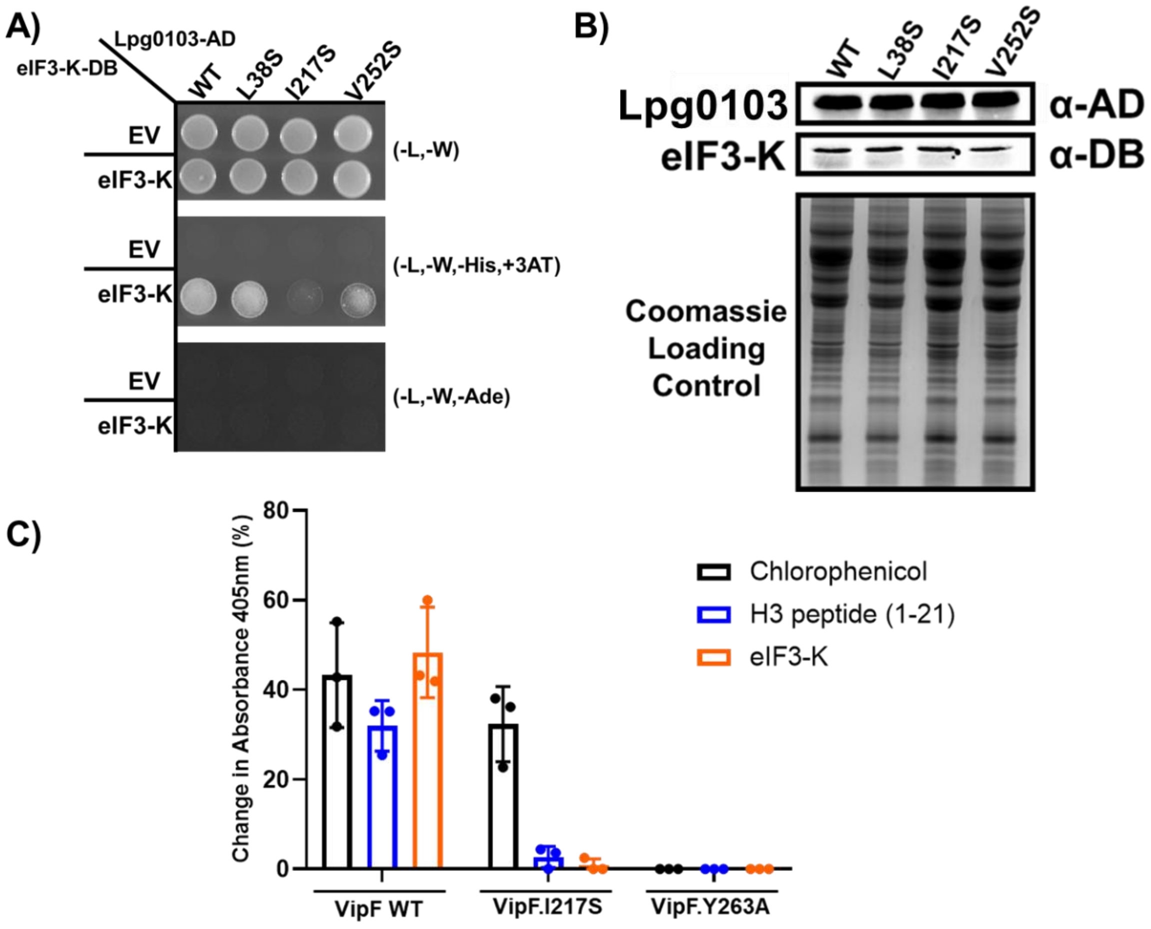

Supplemental Figure 9. I217 is Important for Interactions with eIF3-K and the VipF.I217S mutant is capable of acetylating Chloramphenicol, but not H3 peptide and eIF3-K. (A) Y2H assay of conserved hydrophobic residues substituted with serine tested against eIF3-K. (B) Western blot analysis of assessing the expression of VipF mutants and eIF3-K. (C) DTNB-based assay on the detection of the acetylation of eIF3-K WT, H3 peptide (residues 1-21), and chloramphenicol with VipF.WT, VipF.I217S, and VipF.Y263A. The experiments were performed in three independent experiments with similar results.

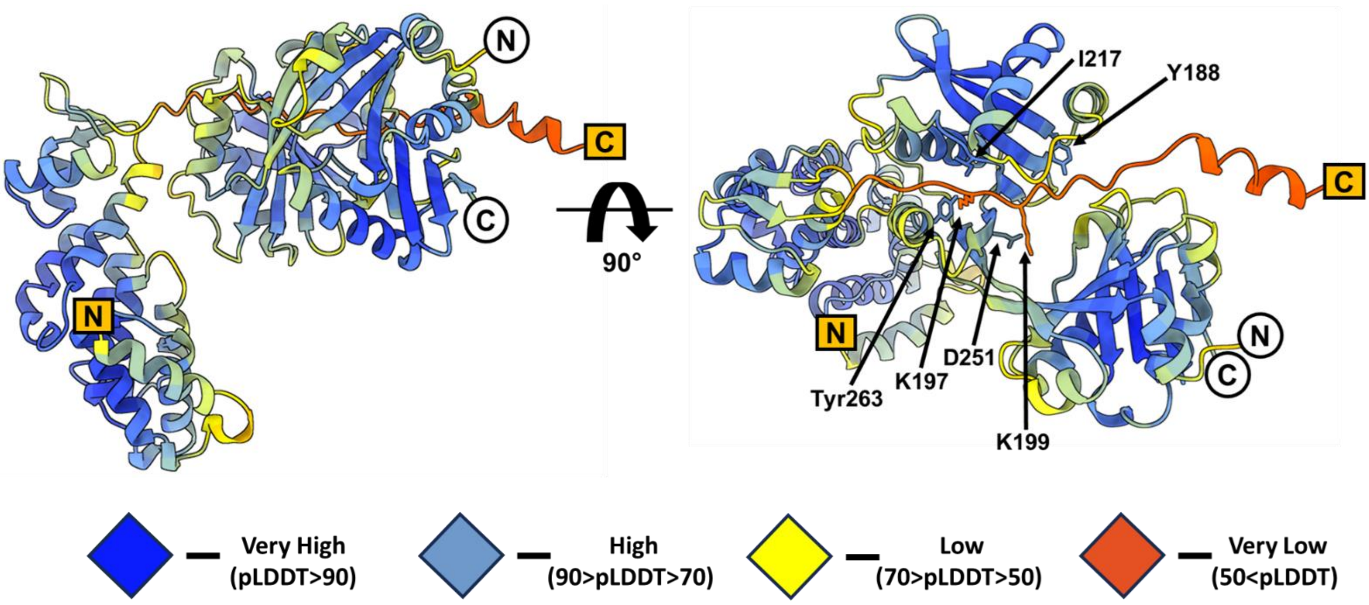

Supplemental Figure 10. The per-residue model confidence score (pLDDT) mapped onto the predicted Lpg0103-eIF3-K complex by AlphaFold2 is shown in Figure 4A. The color of each residue represents a score ranging between 0 to 100. The N- and C- terminal of VipF (Lpg0103) are labeled with a white circle, whereas the termini of eIF3-K are indicated by orange squares. Due to the tail of eIF3-K being disordered, Alphafold2 is expected to produce a low confidence score of this region.

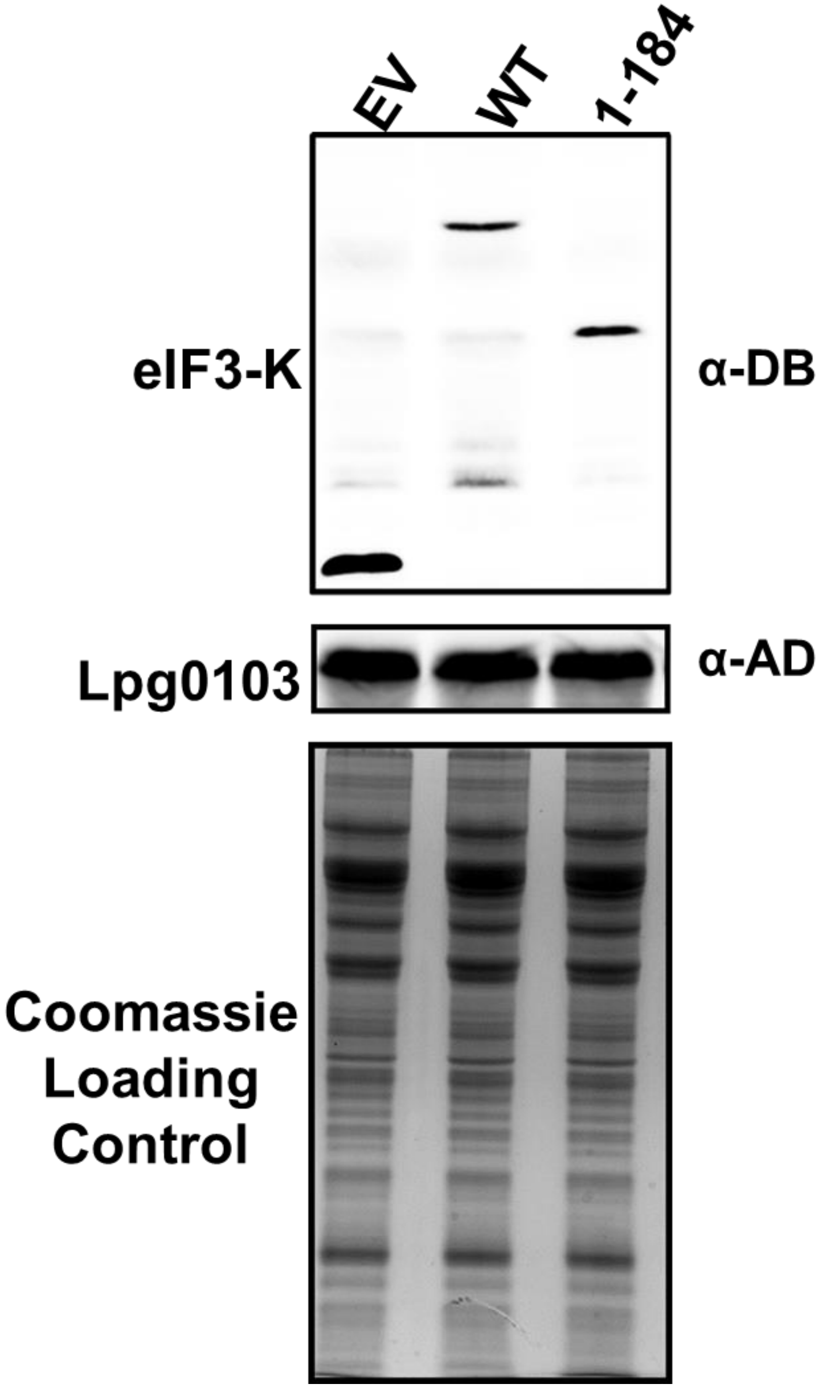

Supplemental Figure S11. Western blot analysis to assess the expression of eIF3-K WT and eIF3-K (1-184) expression in the *S. cerevisiae* Y880 strain used for testing one-on-one interactions with VipF via Y2H in Fig 4B.
