# Supplementary material for: An acetyltransferase effector conserved across Legionella species targets the eukaryotic eIF3 complex to modulate protein translation
Source: mBio. 2024 Feb 9;15(3):e03221-23. doi: 10.1128/mbio.03221-23 (PMC10936415; doi:10.1128/mbio.03221-23)
Supplement: Supplemental Tables — Tables S1 and S2. [file mbio.03221-23-s0002.docx]

**Supplemental Tables
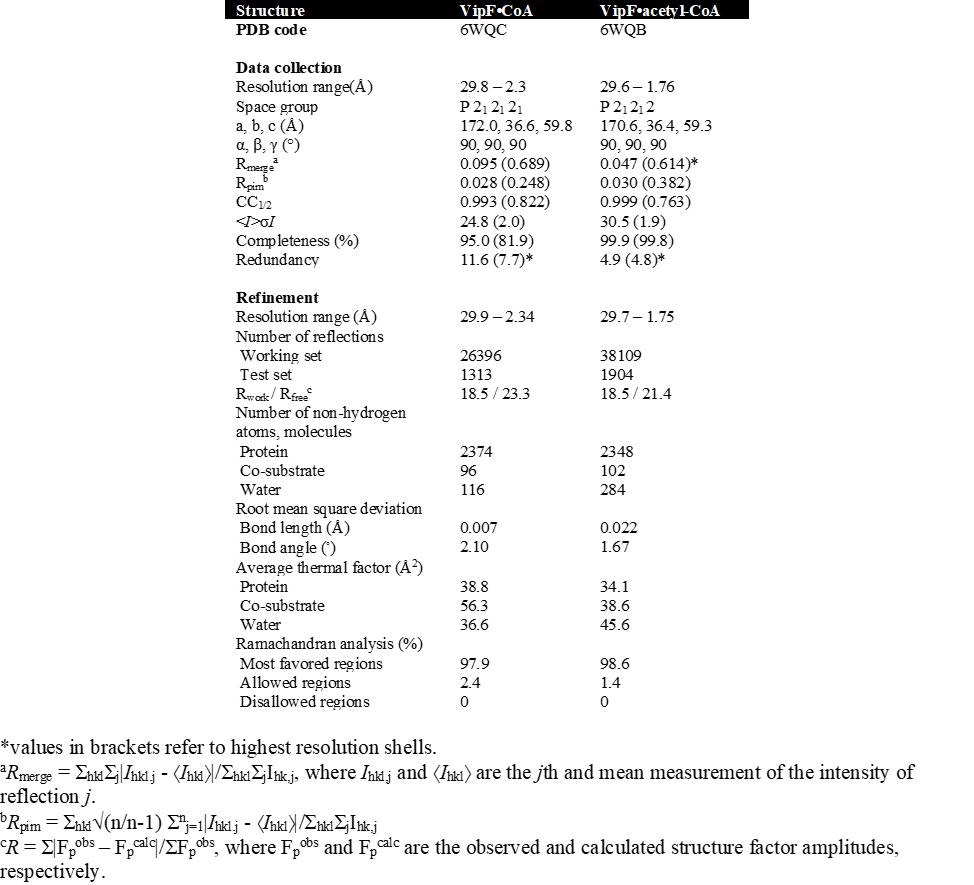
**

**Table S1.** Data collection, phasing, and refinement statistics for Lha0223-AcCoA and Lha0223-CoA

**Table S2.** Total peptide counts from affinity purification mass spectrometry analysis of the putative human host targets of NleG8-1, Lpg0103, and Lha0223. Rows highlighted in yellow indicate proteins associated with the eIF3 complex, while green highlight represents human proteins that co-precipitated with Lpg0103 and Lha0223 but are not associated with the eIF3 complex. Human proteins known to be common contaminants of AP-MS from the CRAPome database were highlighted in orange.

| **Accession Number** | **Protein Description** | **Bead Only** | **NleG8-1** | **Lpg0103** | **Lha0223** |
| --- | --- | --- | --- | --- | --- |
| Lpg0103 | Lpg0103 | 0 | 0 | 366 | 0 |
| Lha0223 | Lha0223 | 0 | 0 | 0 | 270 |
| D2TI20_CITRI (+1) | Putative T3SS effector protein NleG8 OS=Citrobacter rodentium (strain ICC168) OX=637910 GN=nleG8 PE=4 SV=1 | 0 | 103 | 0 | 0 |
| gi\|4503509 | eukaryotic translation initiation factor 3 subunit A [Homo sapiens] | 0 | 0 | 45 | 63 |
| gi\|167234419 | thyroid hormone receptor-associated protein 3 [Homo sapiens] | 0 | 0 | 36 | 35 |
| gi\|33239445 (+3) | eukaryotic translation initiation factor 3 subunit B [Homo sapiens] | 0 | 0 | 33 | 37 |
| gi\|312433975 (+3) | eukaryotic translation initiation factor 3 subunit C isoform a [Homo sapiens] | 0 | 0 | 33 | 28 |
| gi\|7705433 | eukaryotic translation initiation factor 3 subunit L isoform 1 [Homo sapiens] | 0 | 0 | 33 | 33 |
| gi\|4503523 (+1) | eukaryotic translation initiation factor 3 subunit D [Homo sapiens] | 0 | 0 | 22 | 13 |
| gi\|4503513 (+1) | eukaryotic translation initiation factor 3 subunit I [Homo sapiens] | 0 | 0 | 19 | 15 |
| gi\|4503521 | eukaryotic translation initiation factor 3 subunit E [Homo sapiens] | 0 | 0 | 17 | 22 |
| gi\|39777586 (+2) | putative ATP-dependent RNA helicase DHX57 [Homo sapiens] | 0 | 0 | 16 | 24 |
| gi\|4503515 | eukaryotic translation initiation factor 3 subunit H [Homo sapiens] | 0 | 0 | 14 | 18 |
| gi\|4503519 | eukaryotic translation initiation factor 3 subunit F [Homo sapiens] | 0 | 0 | 13 | 19 |
| gi\|23397429 | eukaryotic translation initiation factor 3 subunit M [Homo sapiens] | 0 | 0 | 11 | 10 |
| gi\|49472822 | eukaryotic translation initiation factor 3 subunit G [Homo sapiens] | 0 | 0 | 10 | 12 |
| gi\|224809408 (+4) | homer protein homolog 3 isoform 1 [Homo sapiens] | 0 | 0 | 10 | 13 |
| gi\|10801345 | eukaryotic translation initiation factor 3 subunit K isoform 1 [Homo sapiens] | 0 | 0 | 8 | 12 |
| gi\|530397283 | PREDICTED: neuroblast differentiation-associated protein AHNAK isoform X3 [Homo sapiens] | 0 | 0 | 13 | 15 |
| gi\|83281438 | eukaryotic translation initiation factor 3 subunit J isoform 1 [Homo sapiens] | 0 | 0 | 3 | 7 |
| gi\|28557788 (+2) | sperm-specific antigen 2 isoform 2 [Homo sapiens] | 0 | 0 | 7 | 5 |
| gi\|55925576 | insulin-like growth factor-binding protein 2 precursor [Homo sapiens] | 0 | 0 | 6 | 10 |
| gi\|301171467 (+2) | ATP-dependent RNA helicase DDX3X isoform 2 [Homo sapiens] | 0 | 0 | 3 | 13 |
| gi\|578838411 | PREDICTED: transcriptional regulator ATRX isoform X3 [Homo sapiens] | 0 | 0 | 7 | 24 |
| gi\|767974325 | PREDICTED: protein phosphatase 1 regulatory subunit 12A isoform X1 [Homo sapiens] | 0 | 0 | 7 | 24 |
| gi\|530360925 (+1) | PREDICTED: lysine-specific histone demethylase 1A isoform X1 [Homo sapiens] | 0 | 0 | 8 | 5 |
| gi\|50053795 (+1) | eukaryotic translation initiation factor 4B isoform 2 [Homo sapiens] | 0 | 0 | 11 | 20 |
| gi\|666335584 (+1) | bcl-2-associated transcription factor 1 isoform 4 [Homo sapiens] | 0 | 0 | 19 | 22 |
| gi\|20270337 | RNA polymerase-associated protein LEO1 isoform 1 [Homo sapiens] | 0 | 0 | 11 | 12 |
| gi\|75677353 | ATPase family AAA domain-containing protein 3B [Homo sapiens] | 0 | 0 | 11 | 13 |
| gi\|42518070 | tight junction protein ZO-2 isoform 1 [Homo sapiens] | 0 | 0 | 7 | 23 |
| gi\|4758302 | enhancer of rudimentary homolog [Homo sapiens] | 0 | 0 | 7 | 5 |
| gi\|22325356 (+1) | PALM2-AKAP2 protein isoform 2 [Homo sapiens] | 0 | 0 | 6 | 16 |
| gi\|312222779 (+3) | NAD kinase isoform 1 [Homo sapiens] | 0 | 0 | 6 | 9 |
| gi\|528881280 (+1) | protein kinase C-binding protein 1 isoform h [Homo sapiens] | 0 | 0 | 6 | 5 |
| gi\|53829368 (+1) | neuralized-like protein 4 isoform 2 [Homo sapiens] | 0 | 0 | 6 | 4 |
| gi\|216548085 (+3) | leucine zipper protein 1 [Homo sapiens] | 0 | 0 | 5 | 11 |
| gi\|148368962 (+12) | pseudopodium-enriched atypical kinase 1 [Homo sapiens] | 0 | 0 | 5 | 10 |
| gi\|323276616 | COMMD3-BMI1 read-through protein [Homo sapiens] | 0 | 0 | 5 | 6 |
| gi\|15147335 | nuclear receptor coactivator 5 [Homo sapiens] | 0 | 0 | 5 | 6 |
| gi\|5729864 | HBS1-like protein isoform 1 [Homo sapiens] | 0 | 0 | 5 | 13 |
| gi\|20911035 | peptidyl-prolyl cis-trans isomerase-like 4 [Homo sapiens] | 0 | 0 | 5 | 2 |
| gi\|112734859 | zinc finger protein 654 [Homo sapiens] | 0 | 0 | 5 | 2 |
| gi\|66346683 | plasminogen activator inhibitor 1 RNA-binding protein isoform 3 [Homo sapiens] | 0 | 0 | 4 | 4 |
| gi\|14149716 | protein phosphatase 1 regulatory subunit 12C isoform a [Homo sapiens] | 0 | 0 | 4 | 11 |
| gi\|109255228 (+12) | centrosomal protein of 170 kDa isoform alpha [Homo sapiens] | 0 | 0 | 4 | 21 |
| gi\|296011010 (+17) | protein FAM208B [Homo sapiens] | 0 | 0 | 4 | 9 |
| gi\|47078276 | choline kinase alpha isoform a [Homo sapiens] | 0 | 0 | 4 | 9 |
| gi\|151301219 (+3) | protein arginine N-methyltransferase 1 isoform 3 [Homo sapiens] | 0 | 0 | 4 | 3 |
| gi\|62244004 (+5) | SAFB-like transcription modulator isoform b [Homo sapiens] | 0 | 0 | 4 | 3 |
| gi\|4505423 | protein NOV homolog precursor [Homo sapiens] | 0 | 0 | 4 | 7 |
| gi\|197313748 (+3) | histone-lysine N-methyltransferase SETD2 [Homo sapiens] | 0 | 0 | 4 | 3 |
| gi\|24308227 (+4) | zinc finger protein 687 [Homo sapiens] | 0 | 0 | 4 | 4 |
| gi\|214010181 (+2) | amyloid-like protein 2 isoform 2 precursor [Homo sapiens] | 0 | 0 | 4 | 1 |
| gi\|301171467 (+2) | ATP-dependent RNA helicase DDX3X isoform 2 [Homo sapiens] | 0 | 0 | 3 | 13 |
| gi\|156630992 (+3) | OTU domain-containing protein 4 isoform 3 [Homo sapiens] | 0 | 0 | 3 | 8 |
| gi\|255918139 (+2) | chromosome alignment-maintaining phosphoprotein 1 [Homo sapiens] | 0 | 0 | 3 | 8 |
| gi\|261399907 (+8) | rho GTPase-activating protein 25 isoform a [Homo sapiens] | 0 | 0 | 3 | 4 |
| gi\|4506567 (+5) | mRNA cap guanine-N7 methyltransferase [Homo sapiens] | 0 | 0 | 3 | 3 |
| gi\|17999539 (+3) | pre-mRNA-splicing factor ATP-dependent RNA helicase PRP16 [Homo sapiens] | 0 | 0 | 3 | 8 |
| gi\|530394600 (+1) | PREDICTED: nucleolar and coiled-body phosphoprotein 1 isoform X1 [Homo sapiens] | 0 | 0 | 3 | 11 |
| gi\|259906018 (+5) | apoptotic chromatin condensation inducer in the nucleus isoform 2 [Homo sapiens] | 0 | 0 | 3 | 12 |
| gi\|530404193 | PREDICTED: YLP motif-containing protein 1 isoform X1 [Homo sapiens] | 0 | 0 | 3 | 13 |
| gi\|156105705 (+3) | SNF-related serine/threonine-protein kinase [Homo sapiens] | 0 | 0 | 3 | 11 |
| gi\|116008442 (+12) | zinc finger CCCH domain-containing protein 13 [Homo sapiens] | 0 | 0 | 3 | 5 |
| gi\|14249158 (+1) | hepatoma-derived growth factor-related protein 2 isoform 2 [Homo sapiens] | 0 | 0 | 3 | 9 |
| gi\|4506005 (+1) | serine/threonine-protein phosphatase PP1-beta catalytic subunit isoform 1 [Homo sapiens] | 0 | 0 | 3 | 7 |
| gi\|50233812 (+1) | zinc finger CCCH domain-containing protein 14 isoform 2 [Homo sapiens] | 0 | 0 | 3 | 10 |
| gi\|7661636 | pre-mRNA-splicing factor SYF2 isoform 1 [Homo sapiens] | 0 | 0 | 3 | 4 |
| gi\|4758024 | coilin [Homo sapiens] | 0 | 0 | 3 | 3 |
| gi\|30520329 (+4) | glycerol-3-phosphate acyltransferase 6 [Homo sapiens] | 0 | 0 | 3 | 0 |
| gi\|4826690 (+1) | ATP-dependent RNA helicase DHX8 isoform 1 [Homo sapiens] | 0 | 0 | 3 | 3 |
| gi\|223005950 | SH3KBP1-binding protein 1 [Homo sapiens] | 0 | 0 | 2 | 9 |
| gi\|41327771 | probable ATP-dependent RNA helicase DDX23 [Homo sapiens] | 0 | 0 | 2 | 7 |
| gi\|4506003 (+1) | serine/threonine-protein phosphatase PP1-alpha catalytic subunit isoform 1 [Homo sapiens] | 0 | 0 | 2 | 4 |
| gi\|10863889 | U4/U6.U5 tri-snRNP-associated protein 1 [Homo sapiens] | 0 | 0 | 2 | 6 |
| gi\|115298668 | U5 small nuclear ribonucleoprotein 40 kDa protein [Homo sapiens] | 0 | 0 | 2 | 2 |
| gi\|156766043 (+1) | PERQ amino acid-rich with GYF domain-containing protein 2 isoform b [Homo sapiens] | 0 | 0 | 2 | 6 |
| gi\|90193622 | integrator complex subunit 6 isoform b [Homo sapiens] | 0 | 0 | 2 | 4 |
| gi\|376319203 | U4/U6.U5 tri-snRNP-associated protein 2 isoform 3 [Homo sapiens] | 0 | 0 | 2 | 1 |
| gi\|21040371 (+1) | ATP-dependent RNA helicase DDX39A [Homo sapiens] | 0 | 0 | 2 | 0 |
| gi\|4826998 (+2) | splicing factor, proline- and glutamine-rich [Homo sapiens] | 0 | 0 | 2 | 2 |
| gi\|283837894 (+5) | zinc finger MYM-type protein 3 isoform 2 [Homo sapiens] | 0 | 0 | 2 | 1 |
| gi\|157388957 (+7) | schlafen family member 11 [Homo sapiens] | 0 | 0 | 2 | 1 |
| gi\|7662010 (+7) | zinc finger protein 516 [Homo sapiens] | 0 | 0 | 2 | 2 |
| gi\|51479192 | E3 ubiquitin-protein ligase RING1 [Homo sapiens] | 0 | 0 | 2 | 5 |
| gi\|313851001 (+1) | myosin light chain 6B [Homo sapiens] | 0 | 0 | 2 | 3 |
| gi\|767938481 (+2) | PREDICTED: probable tRNA(His) guanylyltransferase isoform X1 [Homo sapiens] | 0 | 0 | 2 | 2 |
| gi\|123173757 | ribonucleoprotein PTB-binding 1 [Homo sapiens] | 0 | 0 | 2 | 3 |
| gi\|16306580 (+1) | lysine-specific demethylase 2A isoform a [Homo sapiens] | 0 | 0 | 2 | 9 |
| gi\|5032179 | transcription intermediary factor 1-beta [Homo sapiens] | 0 | 0 | 2 | 3 |
| gi\|24431950 (+2) | U4/U6 small nuclear ribonucleoprotein Prp4 isoform 1 [Homo sapiens] | 0 | 0 | 2 | 4 |
| gi\|767918750 | PREDICTED: Holliday junction recognition protein isoform X1 [Homo sapiens] | 0 | 0 | 2 | 2 |
| gi\|24432106 | cell cycle and apoptosis regulator protein 2 [Homo sapiens] | 0 | 0 | 2 | 0 |
| gi\|6005747 (+2) | E3 ubiquitin-protein ligase RING2 [Homo sapiens] | 0 | 0 | 2 | 6 |
| gi\|444299649 (+3) | protein CASC5 isoform 1 [Homo sapiens] | 0 | 0 | 2 | 7 |
| gi\|195976782 | RNA polymerase-associated protein RTF1 homolog [Homo sapiens] | 0 | 0 | 2 | 4 |
| gi\|161169023 (+5) | transcription elongation factor SPT5 isoform a [Homo sapiens] | 0 | 0 | 2 | 9 |
| gi\|14790190 | msx2-interacting protein [Homo sapiens] | 0 | 0 | 2 | 8 |
| gi\|14150185 | zinc finger BED domain-containing protein 3 [Homo sapiens] | 0 | 0 | 2 | 2 |
| gi\|21464101 | 14-3-3 protein gamma [Homo sapiens] | 0 | 0 | 2 | 1 |
| gi\|24762236 (+1) | pre-mRNA-splicing factor 38A [Homo sapiens] | 0 | 0 | 2 | 4 |
| gi\|12758125 (+1) | ribosomal RNA-processing protein 8 [Homo sapiens] | 0 | 0 | 2 | 5 |
| gi\|118600961 (+4) | ral GTPase-activating protein subunit alpha-2 [Homo sapiens] | 0 | 0 | 2 | 1 |
| gi\|767992815 | PREDICTED: uncharacterized protein LOC105371478 [Homo sapiens] | 0 | 0 | 2 | 2 |
| gi\|55770830 (+1) | E3 SUMO-protein ligase CBX4 [Homo sapiens] | 0 | 0 | 2 | 1 |
| gi\|14043072 (+2) | heterogeneous nuclear ribonucleoproteins A2/B1 isoform B1 [Homo sapiens] | 0 | 0 | 2 | 0 |
| gi\|336176064 (+7) | RNA-binding protein 39 isoform c [Homo sapiens] | 0 | 0 | 2 | 1 |
| gi\|20162566 | M-phase-specific PLK1-interacting protein [Homo sapiens] | 0 | 0 | 2 | 1 |
| gi\|281182690 (+9) | WASH complex subunit FAM21C isoform 1 [Homo sapiens] | 0 | 0 | 2 | 3 |
| gi\|530372904 (+1) | PREDICTED: WD repeat-containing protein 48 isoform X1 [Homo sapiens] | 0 | 0 | 2 | 3 |
| gi\|33354279 (+2) | GPALPP motifs-containing protein 1 [Homo sapiens] | 0 | 0 | 2 | 2 |
| gi\|28557677 (+1) | PHD finger protein 6 isoform 1 [Homo sapiens] | 0 | 0 | 2 | 2 |
| gi\|7661878 (+6) | kinesin-like protein KIF14 [Homo sapiens] | 0 | 0 | 2 | 4 |
| gi\|26051231 | serine beta-lactamase-like protein LACTB, mitochondrial isoform a precursor [Homo sapiens] | 0 | 0 | 2 | 5 |
| gi\|4507951 | 14-3-3 protein eta [Homo sapiens] | 0 | 0 | 2 | 0 |
| gi\|255982614 | putative pre-mRNA-splicing factor ATP-dependent RNA helicase DHX16 isoform 1 [Homo sapiens] | 0 | 0 | 2 | 2 |
| gi\|7657198 | probable dimethyladenosine transferase [Homo sapiens] | 0 | 0 | 2 | 0 |
| gi\|578831568 (+7) | PREDICTED: kinase suppressor of Ras 1 isoform X2 [Homo sapiens] | 0 | 0 | 2 | 1 |
| gi\|187608777 | tonsoku-like protein [Homo sapiens] | 0 | 0 | 2 | 2 |
| gi\|157168349 | F-box/LRR-repeat protein 19 isoform 1 [Homo sapiens] | 0 | 0 | 2 | 1 |
| gi\|767917020 (+6) | PREDICTED: shugoshin-like 2 isoform X2 [Homo sapiens] | 0 | 0 | 2 | 1 |
| gi\|291084575 | MAP7 domain-containing protein 3 isoform 3 [Homo sapiens] | 0 | 0 | 2 | 0 |
| gi\|4508047 (+4) | zyxin [Homo sapiens] | 0 | 0 | 2 | 0 |
| gi\|208022622 (+2) | ubiquitin-40S ribosomal protein S27a precursor [Homo sapiens] | 0 | 0 | 2 | 0 |
| gi\|14150100 | cancer-related nucleoside-triphosphatase [Homo sapiens] | 0 | 0 | 1 | 3 |
| gi\|768047504 (+2) | PREDICTED: protein PRRC2A isoform X9 [Homo sapiens] | 0 | 0 | 1 | 10 |
| gi\|150418007 (+5) | E3 SUMO-protein ligase RanBP2 [Homo sapiens] | 0 | 0 | 1 | 0 |
| gi\|20149598 (+1) | mitochondrial dicarboxylate carrier isoform 2 [Homo sapiens] | 0 | 0 | 1 | 1 |
| gi\|13027644 (+4) | small nuclear ribonucleoprotein-associated protein N [Homo sapiens] | 0 | 0 | 1 | 2 |
| gi\|51599156 (+6) | chromodomain-helicase-DNA-binding protein 4 isoform 1 [Homo sapiens] | 0 | 0 | 1 | 2 |
| gi\|767912423 | PREDICTED: lamin-B receptor isoform X2 [Homo sapiens] | 0 | 0 | 1 | 0 |
| gi\|14165435 (+9) | heterogeneous nuclear ribonucleoprotein K isoform b [Homo sapiens] | 0 | 0 | 1 | 2 |
| gi\|767948171 (+1) | PREDICTED: MICOS complex subunit MIC19 isoform X1 [Homo sapiens] | 0 | 0 | 1 | 0 |
| gi\|11342664 (+4) | interferon-induced GTP-binding protein Mx2 [Homo sapiens] | 0 | 0 | 1 | 9 |
| gi\|115298678 | complement C3 precursor [Homo sapiens] | 0 | 0 | 1 | 1 |
| gi\|31377667 | lon protease homolog 2, peroxisomal isoform 1 [Homo sapiens] | 0 | 0 | 1 | 2 |
| gi\|4504897 (+1) | importin subunit alpha-1 [Homo sapiens] | 0 | 0 | 1 | 5 |
| gi\|28178821 (+1) | isocitrate dehydrogenase [NAD] subunit beta, mitochondrial isoform a precursor [Homo sapiens] | 0 | 0 | 1 | 0 |
| gi\|101943240 (+4) | general transcription factor 3C polypeptide 1 isoform 1 [Homo sapiens] | 0 | 0 | 1 | 3 |
| gi\|530402576 (+13) | PREDICTED: RNA-binding protein 26 isoform X5 [Homo sapiens] | 0 | 0 | 1 | 4 |
| gi\|167466160 | H(+)/Cl(-) exchange transporter 7 isoform b [Homo sapiens] | 0 | 0 | 1 | 0 |
| gi\|5454064 | RNA-binding protein 14 isoform 1 [Homo sapiens] | 0 | 0 | 1 | 6 |
| gi\|260898743 (+3) | NADH dehydrogenase [ubiquinone] iron-sulfur protein 2, mitochondrial isoform 2 precursor [Homo sapiens] | 0 | 0 | 1 | 1 |
| gi\|767939637 | PREDICTED: zinc finger protein 318 isoform X1 [Homo sapiens] | 0 | 0 | 1 | 4 |
| gi\|31083028 | nocturnin [Homo sapiens] | 0 | 0 | 1 | 0 |
| gi\|24497618 | ATPase family AAA domain-containing protein 2 [Homo sapiens] | 0 | 0 | 1 | 5 |
| gi\|344925845 | REST corepressor 1 [Homo sapiens] | 0 | 0 | 1 | 4 |
| gi\|373938427 (+12) | nucleolar protein 8 isoform b [Homo sapiens] | 0 | 0 | 1 | 7 |
| g+689:763i\|545309640 | eukaryotic translation initiation factor 4E type 2 isoform C [Homo sapiens] | 0 | 0 | 1 | 4 |
| gi\|171543895 | ataxin-2 [Homo sapiens] | 0 | 0 | 1 | 3 |
| gi\|40018629 (+2) | WASH complex subunit 7 isoform 2 [Homo sapiens] | 0 | 0 | 1 | 0 |
| gi\|55956904 (+1) | mitogen-activated protein kinase kinase kinase 4 isoform a [Homo sapiens] | 0 | 0 | 1 | 0 |
| gi\|42476169 | RNA polymerase II-associated factor 1 homolog isoform 1 [Homo sapiens] | 0 | 0 | 1 | 2 |
| gi\|21450828 (+1) | RILP-like protein 2 [Homo sapiens] | 0 | 0 | 1 | 6 |
| gi\|530372543 (+2) | PREDICTED: NK-tumor recognition protein isoform X1 [Homo sapiens] | 0 | 0 | 1 | 3 |
| gi\|14249678 (+1) | splicing factor 45 [Homo sapiens] | 0 | 0 | 1 | 6 |
| gi\|194097365 | pre-rRNA processing protein FTSJ3 [Homo sapiens] | 0 | 0 | 1 | 1 |
| gi\|27477136 | zinc finger CCCH-type antiviral protein 1 isoform 1 [Homo sapiens] | 0 | 0 | 1 | 0 |
| gi\|4758556 (+1) | U4/U6 small nuclear ribonucleoprotein Prp3 [Homo sapiens] | 0 | 0 | 1 | 2 |
| gi\|42560244 (+2) | peptidyl-prolyl cis-trans isomerase G [Homo sapiens] | 0 | 0 | 1 | 5 |
| gi\|17149830 (+2) | ARF GTPase-activating protein GIT2 isoform 1 [Homo sapiens] | 0 | 0 | 1 | 3 |
| gi\|221307494 (+1) | chromodomain Y-like protein isoform a [Homo sapiens] | 0 | 0 | 1 | 1 |
| gi\|64276486 | peptidyl-prolyl cis-trans isomerase CWC27 homolog isoform 1 [Homo sapiens] | 0 | 0 | 1 | 2 |
| gi\|190014623 (+1) | exosome complex exonuclease RRP44 isoform a [Homo sapiens] | 0 | 0 | 1 | 2 |
| gi\|38488727 (+2) | serine/Arginine-related protein 53 isoform 1 [Homo sapiens] | 0 | 0 | 1 | 4 |
| gi\|7661786 | NADH dehydrogenase [ubiquinone] 1 alpha subcomplex assembly factor 4 [Homo sapiens] | 0 | 0 | 1 | 0 |
| gi\|254692833 (+1) | NHS-like protein 2 [Homo sapiens] | 0 | 0 | 1 | 3 |
| gi\|217330641 (+1) | protein IWS1 homolog [Homo sapiens] | 0 | 0 | 1 | 5 |
| gi\|14149807 | nuclear speckle splicing regulatory protein 1 isoform 1 [Homo sapiens] | 0 | 0 | 1 | 5 |
| gi\|41327781 | NADH dehydrogenase [ubiquinone] 1 alpha subcomplex assembly factor 3 isoform a precursor [Homo sapiens] | 0 | 0 | 1 | 0 |
| gi\|33285002 (+1) | selenoprotein S isoform 2 [Homo sapiens] | 0 | 0 | 1 | 3 |
| gi\|164664518 (+3) | probable ATP-dependent RNA helicase DDX6 [Homo sapiens] | 0 | 0 | 1 | 5 |
| gi\|224586815 (+1) | Golgi apparatus protein 1 isoform 2 precursor [Homo sapiens] | 0 | 0 | 1 | 2 |
| gi\|767936123 (+1) | PREDICTED: lysine-specific demethylase 3B isoform X1 [Homo sapiens] | 0 | 0 | 1 | 4 |
| gi\|578811957 (+5) | PREDICTED: negative elongation factor E isoform X1 [Homo sapiens] | 0 | 0 | 1 | 1 |
| gi\|108773782 (+1) | ATP-binding cassette sub-family E member 1 [Homo sapiens] | 0 | 0 | 1 | 1 |
| gi\|767968910 | PREDICTED: liprin-alpha-1 isoform X6 [Homo sapiens] | 0 | 0 | 1 | 4 |
| gi\|31044432 | LEM domain-containing protein 2 isoform 1 [Homo sapiens] | 0 | 0 | 1 | 3 |
| gi\|4506619 | 60S ribosomal protein L24 [Homo sapiens] | 0 | 0 | 1 | 1 |
| gi\|4759154 | synaptosomal-associated protein 29 [Homo sapiens] | 0 | 0 | 1 | 3 |
| gi\|29570791 (+2) | casein kinase II subunit alpha isoform a [Homo sapiens] | 0 | 0 | 1 | 5 |
| gi\|530386297 (+7) | PREDICTED: PERQ amino acid-rich with GYF domain-containing protein 1 isoform X1 [Homo sapiens] | 0 | 0 | 1 | 7 |
| gi\|544346219 | E3 ubiquitin-protein ligase MARCH7 isoform c [Homo sapiens] | 0 | 0 | 1 | 1 |
| gi\|321117267 | HSPE1-MOB4 protein [Homo sapiens] | 0 | 0 | 1 | 1 |
| gi\|5729862 (+1) | histone RNA hairpin-binding protein [Homo sapiens] | 0 | 0 | 1 | 2 |
| gi\|13027630 | protein DGCR14 [Homo sapiens] | 0 | 0 | 1 | 3 |
| gi\|15208663 (+2) | tripartite motif-containing protein 14 [Homo sapiens] | 0 | 0 | 1 | 4 |
| gi\|268607506 | protein phosphatase 1 regulatory subunit 12B isoform a [Homo sapiens] | 0 | 0 | 1 | 4 |
| gi\|20357522 (+4) | lysine-specific demethylase 3A [Homo sapiens] | 0 | 0 | 1 | 0 |
| gi\|71725360 (+1) | zinc finger protein 609 [Homo sapiens] | 0 | 0 | 1 | 4 |
| gi\|21361159 (+1) | pre-mRNA-splicing regulator WTAP isoform 1 [Homo sapiens] | 0 | 0 | 1 | 5 |
| gi\|208431793 (+5) | dynactin subunit 4 isoform a [Homo sapiens] | 0 | 0 | 1 | 0 |
| gi\|214830438 (+2) | sequestosome-1 isoform 2 [Homo sapiens] | 0 | 0 | 1 | 0 |
| gi\|4501993 | alkyldihydroxyacetonephosphate synthase, peroxisomal precursor [Homo sapiens] | 0 | 0 | 1 | 0 |
| gi\|271398350 (+3) | nucleoporin NDC1 isoform 1 [Homo sapiens] | 0 | 0 | 1 | 0 |
| gi\|14249338 | BUD13 homolog isoform 1 [Homo sapiens] | 0 | 0 | 1 | 3 |
| gi\|209862833 (+5) | amyloid beta A4 protein isoform d [Homo sapiens] | 0 | 0 | 1 | 1 |
| gi\|10835218 (+1) | DNA topoisomerase 3-alpha [Homo sapiens] | 0 | 0 | 1 | 2 |
| gi\|4757834 | BAG family molecular chaperone regulator 2 [Homo sapiens] | 0 | 0 | 1 | 0 |
| gi\|5902158 | RING finger protein 113A [Homo sapiens] | 0 | 0 | 1 | 2 |
| gi\|219842247 (+5) | periphilin-1 isoform 7 [Homo sapiens] | 0 | 0 | 1 | 2 |
| gi\|157364937 | OTU domain-containing protein 6B isoform 1 [Homo sapiens] | 0 | 0 | 1 | 4 |
| gi\|18079218 (+10) | oxysterol-binding protein-related protein 8 isoform a [Homo sapiens] | 0 | 0 | 1 | 1 |
| gi\|221307566 (+6) | C-myc promoter-binding protein isoform 2 [Homo sapiens] | 0 | 0 | 1 | 0 |
| gi\|332164779 (+6) | pyruvate kinase PKM isoform e [Homo sapiens] | 0 | 0 | 1 | 0 |
| gi\|23618867 | sideroflexin-1 [Homo sapiens] | 0 | 0 | 1 | 0 |
| gi\|256818794 (+7) | eukaryotic translation initiation factor 4E transporter isoform a [Homo sapiens] | 0 | 0 | 1 | 1 |
| gi\|16262452 (+1) | cingulin [Homo sapiens] | 0 | 0 | 1 | 3 |
| gi\|164698438 | thymidine kinase, cytosolic [Homo sapiens] | 0 | 0 | 1 | 0 |
| gi\|530422386 (+2) | PREDICTED: protein DDX26B isoform X1 [Homo sapiens] | 0 | 0 | 1 | 0 |
| gi\|187829640 (+8) | vasculin isoform 2 [Homo sapiens] | 0 | 0 | 1 | 3 |
| gi\|21618347 (+3) | dual specificity mitogen-activated protein kinase kinase 3 isoform A [Homo sapiens] | 0 | 0 | 1 | 0 |
| gi\|21361499 | sentrin-specific protease 3 [Homo sapiens] | 0 | 0 | 1 | 1 |
| gi\|768056179 (+1) | PREDICTED: RING1 and YY1-binding protein isoform X1 [Homo sapiens] | 0 | 0 | 1 | 3 |
| gi\|122939208 | nuclear autoantigen Sp-100 isoform 1 [Homo sapiens] | 0 | 0 | 1 | 2 |
| gi\|164663816 (+2) | LIM domain-binding protein 1 isoform 1 [Homo sapiens] | 0 | 0 | 1 | 1 |
| gi\|767927731 (+1) | PREDICTED: fragile X mental retardation syndrome-related protein 1 isoform X1 [Homo sapiens] | 0 | 0 | 1 | 1 |
| gi\|56549113 | lariat debranching enzyme [Homo sapiens] | 0 | 0 | 1 | 3 |
| gi\|431822375 (+4) | dedicator of cytokinesis protein 7 isoform 1 [Homo sapiens] | 0 | 0 | 1 | 2 |
| gi\|302699211 (+11) | pogo transposable element with ZNF domain isoform 3 [Homo sapiens] | 0 | 0 | 1 | 2 |
| gi\|27477070 (+1) | TATA-binding protein-associated factor 172 [Homo sapiens] | 0 | 0 | 1 | 0 |
| gi\|530396040 (+8) | PREDICTED: protein-methionine sulfoxide oxidase MICAL2 isoform X1 [Homo sapiens] | 0 | 0 | 1 | 2 |
| gi\|530360621 (+3) | PREDICTED: migration and invasion-inhibitory protein isoform X2 [Homo sapiens] | 0 | 0 | 1 | 0 |
| gi\|6857818 | nucleoplasmin-3 [Homo sapiens] | 0 | 0 | 1 | 0 |
| gi\|190610012 (+1) | zinc finger protein 24 [Homo sapiens] | 0 | 0 | 1 | 2 |
| gi\|31543910 (+2) | ubiquitin carboxyl-terminal hydrolase 1 [Homo sapiens] | 0 | 0 | 1 | 0 |
| gi\|24497603 (+4) | nuclear pore glycoprotein p62 [Homo sapiens] | 0 | 0 | 1 | 1 |
| gi\|570359575 (+3) | death-associated protein kinase 1 [Homo sapiens] | 0 | 0 | 1 | 0 |
| gi\|4506961 (+2) | gem-associated protein 2 isoform alpha [Homo sapiens] | 0 | 0 | 1 | 0 |
| gi\|530382396 (+4) | PREDICTED: 39S ribosomal protein L14, mitochondrial isoform X2 [Homo sapiens] | 0 | 0 | 1 | 0 |
| gi\|5901998 | U6 snRNA-associated Sm-like protein LSm6 [Homo sapiens] | 0 | 0 | 1 | 0 |
| gi\|156564398 | target of EGR1 protein 1 [Homo sapiens] | 0 | 0 | 1 | 0 |
| gi\|27436969 (+1) | voltage-gated potassium channel subunit beta-2 isoform 2 [Homo sapiens] | 0 | 0 | 1 | 0 |
| gi\|11342680 | beta-centractin [Homo sapiens] | 0 | 0 | 1 | 0 |
| gi\|578830104 | PREDICTED: mannose-P-dolichol utilization defect 1 protein isoform X3 [Homo sapiens] | 0 | 0 | 1 | 0 |
| gi\|11968150 (+8) | GDNF-inducible zinc finger protein 1 [Homo sapiens] | 0 | 0 | 1 | 0 |
| gi\|169636418 | 39S ribosomal protein L38, mitochondrial [Homo sapiens] | 0 | 0 | 1 | 0 |
| gi\|47834348 (+1) | minor histocompatibility protein HA-1 isoform 1 precursor [Homo sapiens] | 0 | 0 | 0 | 4 |
| gi\|238231392 (+6) | dedicator of cytokinesis protein 8 isoform 1 [Homo sapiens] | 0 | 0 | 0 | 6 |
| gi\|530364422 | PREDICTED: protein PRRC2C isoform X6 [Homo sapiens] | 0 | 0 | 0 | 1 |
| gi\|209969703 (+1) | protein RCC2 [Homo sapiens] | 0 | 0 | 0 | 4 |
| gi\|4506489 | replication factor C subunit 3 isoform 1 [Homo sapiens] | 0 | 0 | 0 | 2 |
| gi\|392583868 | SRA stem-loop-interacting RNA-binding protein, mitochondrial isoform 3 precursor [Homo sapiens] | 0 | 0 | 0 | 2 |
| gi\|300192933 | AFG3-like protein 2 [Homo sapiens] | 0 | 0 | 0 | 1 |
| gi\|302318952 (+3) | PML-RARA-regulated adapter molecule 1 [Homo sapiens] | 0 | 0 | 0 | 1 |
| gi\|156616275 (+4) | DNA polymerase delta catalytic subunit [Homo sapiens] | 0 | 0 | 0 | 2 |
| gi\|4506399 (+5) | mRNA export factor [Homo sapiens] | 0 | 0 | 0 | 2 |
| gi\|384475537 (+5) | DNA-directed RNA polymerase III subunit RPC5 isoform 4 [Homo sapiens] | 0 | 0 | 0 | 2 |
| gi\|45446743 (+2) | ATP-dependent RNA helicase DDX42 [Homo sapiens] | 0 | 0 | 0 | 17 |
| gi\|122937227 (+1) | U2 snRNP-associated SURP motif-containing protein [Homo sapiens] | 0 | 0 | 0 | 3 |
| gi\|14043070 (+2) | heterogeneous nuclear ribonucleoprotein A1 isoform b [Homo sapiens] | 0 | 0 | 0 | 2 |
| gi\|33598968 | LIM domain only protein 7 isoform 1 [Homo sapiens] | 0 | 0 | 0 | 1 |
| gi\|199559805 (+1) | phostensin [Homo sapiens] | 0 | 0 | 0 | 1 |
| gi\|119395734 | breast cancer type 2 susceptibility protein [Homo sapiens] | 0 | 0 | 0 | 0 |
| gi\|124378039 (+18) | protein transport protein Sec16A isoform 1 [Homo sapiens] | 0 | 0 | 0 | 1 |
| gi\|331284176 (+2) | nuclear receptor corepressor 2 isoform 2 [Homo sapiens] | 0 | 0 | 0 | 1 |
| gi\|555289980 (+4) | protein SCAF8 isoform b [Homo sapiens] | 0 | 0 | 0 | 4 |
| gi\|162329583 (+3) | cleavage and polyadenylation specificity factor subunit 6 isoform 1 [Homo sapiens] | 0 | 0 | 0 | 4 |
| gi\|40018640 (+1) | parafibromin [Homo sapiens] | 0 | 0 | 0 | 2 |
| gi\|7661950 | RNA polymerase-associated protein CTR9 homolog [Homo sapiens] | 0 | 0 | 0 | 0 |
| gi\|239582772 | uncharacterized protein C2orf47, mitochondrial precursor [Homo sapiens] | 0 | 0 | 0 | 1 |
| gi\|5454084 | serine palmitoyltransferase 1 isoform a [Homo sapiens] | 0 | 0 | 0 | 1 |
| gi\|289577080 (+2) | eukaryotic translation initiation factor 4 gamma 2 isoform 1 [Homo sapiens] | 0 | 0 | 0 | 1 |
| gi\|530362970 (+1) | PREDICTED: ligand-dependent nuclear receptor-interacting factor 1 isoform X1 [Homo sapiens] | 0 | 0 | 0 | 2 |
| gi\|343887347 (+1) | CD2 antigen cytoplasmic tail-binding protein 2 [Homo sapiens] | 0 | 0 | 0 | 2 |
| gi\|4759068 | protein SCO1 homolog, mitochondrial [Homo sapiens] | 0 | 0 | 0 | 1 |
| gi\|332164786 (+4) | replication factor C subunit 5 isoform 4 [Homo sapiens] | 0 | 0 | 0 | 1 |
| gi\|25777730 (+1) | aldehyde dehydrogenase X, mitochondrial precursor [Homo sapiens] | 0 | 0 | 0 | 1 |
| gi\|24308334 (+4) | constitutive coactivator of peroxisome proliferator-activated receptor gamma isoform c [Homo sapiens] | 0 | 0 | 0 | 0 |
| gi\|4502897 (+1) | cleft lip and palate transmembrane protein 1 isoform 2 [Homo sapiens] | 0 | 0 | 0 | 1 |
| gi\|153791350 (+13) | replication initiator 1 isoform 1 [Homo sapiens] | 0 | 0 | 0 | 2 |
| gi\|224028244 (+2) | non-POU domain-containing octamer-binding protein isoform 1 [Homo sapiens] | 0 | 0 | 0 | 5 |
| gi\|530416988 | PREDICTED: 60S ribosomal protein L28 isoform X1 [Homo sapiens] | 0 | 0 | 0 | 1 |
| gi\|4826848 (+1) | NADH dehydrogenase [ubiquinone] 1 alpha subcomplex subunit 5 isoform 1 [Homo sapiens] | 0 | 0 | 0 | 1 |
| gi\|46255026 | BTB/POZ domain-containing protein KCTD3 [Homo sapiens] | 0 | 0 | 0 | 5 |
| gi\|662033848 | cytochrome b-c1 complex subunit 6, mitochondrial isoform 3 precursor [Homo sapiens] | 0 | 0 | 0 | 2 |
| gi\|186928852 | 28S ribosomal protein S7, mitochondrial [Homo sapiens] | 0 | 0 | 0 | 3 |
| gi\|4503481 | elongation factor 1-gamma [Homo sapiens] | 0 | 0 | 0 | 2 |
| gi\|209862765 (+1) | iporin [Homo sapiens] | 0 | 0 | 0 | 2 |
| gi\|116174778 (+12) | centrosome and spindle pole-associated protein 1 isoform 1 [Homo sapiens] | 0 | 0 | 0 | 3 |
| gi\|41327773 (+1) | probable ATP-dependent RNA helicase DDX46 isoform 2 [Homo sapiens] | 0 | 0 | 0 | 4 |
| gi\|194018476 (+2) | TBC1 domain family member 2B isoform b [Homo sapiens] | 0 | 0 | 0 | 3 |
| gi\|530419773 | PREDICTED: phosphatidylserine decarboxylase proenzyme isoform X2 [Homo sapiens] | 0 | 0 | 0 | 1 |
| gi\|21361322 (+1) | tubulin beta-4A chain isoform 3 [Homo sapiens] | 0 | 0 | 0 | 16 |
| gi\|256222019 | ras-related protein Rab-10 [Homo sapiens] | 0 | 0 | 0 | 0 |
| gi\|30181233 (+1) | leucine-rich repeat and calponin homology domain-containing protein 4 isoform 1 [Homo sapiens] | 0 | 0 | 0 | 1 |
| gi\|4503943 (+4) | glutaryl-CoA dehydrogenase, mitochondrial isoform a precursor [Homo sapiens] | 0 | 0 | 0 | 0 |
| gi\|4758138 (+3) | probable ATP-dependent RNA helicase DDX5 [Homo sapiens] | 0 | 0 | 0 | 6 |
| gi\|7661920 | eukaryotic initiation factor 4A-III [Homo sapiens] | 0 | 0 | 0 | 4 |
| gi\|103472005 | antigen KI-67 isoform 1 [Homo sapiens] | 0 | 0 | 0 | 2 |
| gi\|157743247 | F-box/SPRY domain-containing protein 1 [Homo sapiens] | 0 | 0 | 0 | 1 |
| gi\|186928850 (+2) | 28S ribosomal protein S27, mitochondrial isoform 2 [Homo sapiens] | 0 | 0 | 0 | 1 |
| gi\|22035565 (+1) | PAX3- and PAX7-binding protein 1 isoform 1 [Homo sapiens] | 0 | 0 | 0 | 3 |
| gi\|33946327 | nuclear pore complex protein Nup214 [Homo sapiens] | 0 | 0 | 0 | 1 |
| gi\|768016784 | PREDICTED: activity-dependent neuroprotector homeobox protein isoform X2 [Homo sapiens] | 0 | 0 | 0 | 1 |
| gi\|15812178 | zinc finger protein 36, C3H1 type-like 2 [Homo sapiens] | 0 | 0 | 0 | 1 |
| gi\|530403479 (+14) | PREDICTED: signal-induced proliferation-associated 1-like protein 1 isoform X3 [Homo sapiens] | 0 | 0 | 0 | 2 |
| gi\|22027525 (+5) | rho guanine nucleotide exchange factor 6 [Homo sapiens] | 0 | 0 | 0 | 3 |
| gi\|20127519 (+4) | targeting protein for Xklp2 [Homo sapiens] | 0 | 0 | 0 | 3 |
| gi\|217035105 | dnaJ homolog subfamily C member 11 [Homo sapiens] | 0 | 0 | 0 | 1 |
| gi\|88999583 | myosin light polypeptide 6 isoform 2 [Homo sapiens] | 0 | 0 | 0 | 3 |
| gi\|24307899 | GTPase Era, mitochondrial [Homo sapiens] | 0 | 0 | 0 | 1 |
| gi\|156713442 (+1) | proteasome subunit alpha type-4 isoform 1 [Homo sapiens] | 0 | 0 | 0 | 1 |
| gi\|20070260 | negative elongation factor B [Homo sapiens] | 0 | 0 | 0 | 1 |
| gi\|20357529 | guanine nucleotide-binding protein G(I)/G(S)/G(T) subunit beta-2 [Homo sapiens] | 0 | 0 | 0 | 1 |
| gi\|4759160 (+1) | small nuclear ribonucleoprotein Sm D3 [Homo sapiens] | 0 | 0 | 0 | 1 |
| gi\|57863269 | ribosomal RNA processing protein 1 homolog B [Homo sapiens] | 0 | 0 | 0 | 2 |
| gi\|767946929 | PREDICTED: RNA-binding protein 33 isoform X6 [Homo sapiens] | 0 | 0 | 0 | 1 |
| gi\|767957687 | PREDICTED: uncharacterized protein C9orf78 isoform X1 [Homo sapiens] | 0 | 0 | 0 | 4 |
| gi\|21396500 | HIRA-interacting protein 3 isoform 1 [Homo sapiens] | 0 | 0 | 0 | 3 |
| gi\|4505371 (+3) | NADH dehydrogenase [ubiquinone] iron-sulfur protein 8, mitochondrial precursor [Homo sapiens] | 0 | 0 | 0 | 1 |
| gi\|38569394 (+1) | elongator complex protein 1 [Homo sapiens] | 0 | 0 | 0 | 1 |
| gi\|530420151 | PREDICTED: phosphatidylinositol 4-kinase alpha isoform X1 [Homo sapiens] | 0 | 0 | 0 | 1 |
| gi\|38683860 | insulin receptor substrate 2 [Homo sapiens] | 0 | 0 | 0 | 1 |
| gi\|44890068 (+6) | zinc finger MYM-type protein 4 [Homo sapiens] | 0 | 0 | 0 | 1 |
| gi\|15809016 (+7) | myosin regulatory light chain 12B [Homo sapiens] | 0 | 0 | 0 | 1 |
| gi\|56788356 (+2) | tuftelin-interacting protein 11 [Homo sapiens] | 0 | 0 | 0 | 4 |
| gi\|116063534 (+2) | ankyrin repeat domain-containing protein 27 [Homo sapiens] | 0 | 0 | 0 | 5 |
| gi\|122937492 (+18) | SET domain-containing protein 5 isoform 1 [Homo sapiens] | 0 | 0 | 0 | 2 |
| gi\|300192959 (+14) | zinc finger MYM-type protein 2 [Homo sapiens] | 0 | 0 | 0 | 2 |
| gi\|13259510 (+3) | dynactin subunit 1 isoform 1 [Homo sapiens] | 0 | 0 | 0 | 1 |
| gi\|110227615 (+3) | echinoderm microtubule-associated protein-like 3 isoform 3 [Homo sapiens] | 0 | 0 | 0 | 2 |
| gi\|116063573 (+6) | filamin-A isoform 1 [Homo sapiens] | 0 | 0 | 0 | 1 |
| gi\|343478203 (+2) | cytospin-B isoform 1 [Homo sapiens] | 0 | 0 | 0 | 1 |
| gi\|13994259 | 28S ribosomal protein S5, mitochondrial [Homo sapiens] | 0 | 0 | 0 | 1 |
| gi\|192807312 (+17) | transcription activator BRG1 isoform B [Homo sapiens] | 0 | 0 | 0 | 1 |
| gi\|13129098 (+1) | ankyrin repeat and SOCS box protein 8 [Homo sapiens] | 0 | 0 | 0 | 1 |
| gi\|530418381 (+2) | PREDICTED: zinc finger protein 217 isoform X1 [Homo sapiens] | 0 | 0 | 0 | 1 |
| gi\|119226260 | calcium homeostasis endoplasmic reticulum protein [Homo sapiens] | 0 | 0 | 0 | 1 |
| gi\|767964243 | PREDICTED: disks large homolog 5 isoform X1 [Homo sapiens] | 0 | 0 | 0 | 3 |
| gi\|19115966 (+5) | DIS3-like exonuclease 1 isoform 2 [Homo sapiens] | 0 | 0 | 0 | 4 |
| gi\|63025222 (+1) | transforming growth factor beta-1 precursor [Homo sapiens] | 0 | 0 | 0 | 1 |
| gi\|20143480 | phosphatidate cytidylyltransferase 2 [Homo sapiens] | 0 | 0 | 0 | 1 |
| gi\|767968726 (+1) | PREDICTED: splicing factor 1 isoform X2 [Homo sapiens] | 0 | 0 | 0 | 1 |
| gi\|7427519 | DNA replication licensing factor MCM6 [Homo sapiens] | 0 | 0 | 0 | 1 |
| gi\|7661822 | dynein light chain roadblock-type 1 isoform a [Homo sapiens] | 0 | 0 | 0 | 1 |
| gi\|319996623 (+7) | putative RNA-binding protein 15 isoform 2 [Homo sapiens] | 0 | 0 | 0 | 2 |
| gi\|15082258 (+2) | chromobox protein homolog 3 [Homo sapiens] | 0 | 0 | 0 | 1 |
| gi\|148596968 | probable E3 ubiquitin-protein ligase TRIM8 [Homo sapiens] | 0 | 0 | 0 | 1 |
| gi\|530406735 (+14) | PREDICTED: rho GTPase-activating protein 11A isoform X3 [Homo sapiens] | 0 | 0 | 0 | 1 |
| gi\|306482646 (+1) | serine/arginine-rich splicing factor 2 [Homo sapiens] | 0 | 0 | 0 | 1 |
| gi\|149192855 | protein PRRC2B [Homo sapiens] | 0 | 0 | 0 | 3 |
| gi\|768015681 (+7) | PREDICTED: death-inducer obliterator 1 isoform X1 [Homo sapiens] | 0 | 0 | 0 | 1 |
| gi\|262205380 (+6) | protein FAM122B isoform 3 [Homo sapiens] | 0 | 0 | 0 | 3 |
| gi\|146231998 (+3) | ARF GTPase-activating protein GIT1 isoform 1 [Homo sapiens] | 0 | 0 | 0 | 8 |
| gi\|20127479 (+5) | RNA-binding protein 10 isoform 1 [Homo sapiens] | 0 | 0 | 0 | 2 |
| gi\|24308075 | rab11 family-interacting protein 5 [Homo sapiens] | 0 | 0 | 0 | 3 |
| gi\|23111062 (+1) | splicing factor, suppressor of white-apricot homolog isoform 2 [Homo sapiens] | 0 | 0 | 0 | 1 |
| gi\|20336294 (+3) | putative ATP-dependent RNA helicase DHX30 isoform 1 [Homo sapiens] | 0 | 0 | 0 | 0 |
| gi\|4507391 (+3) | transcription factor 12 isoform b [Homo sapiens] | 0 | 0 | 0 | 1 |
| gi\|218751903 (+3) | transcription factor Sp1 isoform b [Homo sapiens] | 0 | 0 | 0 | 1 |
| gi\|11321583 | succinyl-CoA ligase [ADP-forming] subunit beta, mitochondrial precursor [Homo sapiens] | 0 | 0 | 0 | 1 |
| gi\|7710129 | prickle-like protein 3 [Homo sapiens] | 0 | 0 | 0 | 1 |
| gi\|614458221 (+2) | phosphoenolpyruvate carboxykinase [GTP], mitochondrial isoform 1 precursor [Homo sapiens] | 0 | 0 | 0 | 1 |
| gi\|530378843 (+1) | PREDICTED: WD repeat-containing protein 70 isoform X1 [Homo sapiens] | 0 | 0 | 0 | 1 |
| gi\|151301096 (+1) | trimethylguanosine synthase [Homo sapiens] | 0 | 0 | 0 | 3 |
| gi\|767976063 (+3) | PREDICTED: probable RNA-binding protein 19 isoform X1 [Homo sapiens] | 0 | 0 | 0 | 1 |
| gi\|530403266 (+1) | PREDICTED: SNW domain-containing protein 1 isoform X1 [Homo sapiens] | 0 | 0 | 0 | 2 |
| gi\|530369483 (+16) | PREDICTED: DNA annealing helicase and endonuclease ZRANB3 isoform X2 [Homo sapiens] | 0 | 0 | 0 | 1 |
| gi\|4758958 (+3) | cAMP-dependent protein kinase type II-alpha regulatory subunit [Homo sapiens] | 0 | 0 | 0 | 3 |
| gi\|187607323 (+2) | translation initiation factor eIF-2B subunit delta isoform 1 [Homo sapiens] | 0 | 0 | 0 | 1 |
| gi\|283046701 (+16) | la-related protein 4 isoform a [Homo sapiens] | 0 | 0 | 0 | 1 |
| gi\|301336155 (+1) | tRNA (cytosine(34)-C(5))-methyltransferase isoform 2 [Homo sapiens] | 0 | 0 | 0 | 2 |
| gi\|224451133 (+2) | splicing factor, arginine/serine-rich 15 isoform 2 [Homo sapiens] | 0 | 0 | 0 | 2 |
| gi\|24308207 | leucine-rich repeat-containing protein 47 [Homo sapiens] | 0 | 0 | 0 | 1 |
| gi\|387528013 (+2) | phosphatidate phosphatase LPIN1 isoform 3 [Homo sapiens] | 0 | 0 | 0 | 4 |
| gi\|409264581 | zinc finger protein 598 [Homo sapiens] | 0 | 0 | 0 | 1 |
| gi\|767926921 | PREDICTED: phospholipase D1 isoform X3 [Homo sapiens] | 0 | 0 | 0 | 2 |
| gi\|20336305 (+7) | B-cell lymphoma/leukemia 11A isoform 1 [Homo sapiens] | 0 | 0 | 0 | 2 |
| gi\|33356547 | DNA replication licensing factor MCM2 [Homo sapiens] | 0 | 0 | 0 | 2 |
| gi\|7706607 (+1) | inner nuclear membrane protein Man1 isoform 1 [Homo sapiens] | 0 | 0 | 0 | 2 |
| gi\|215422366 (+3) | death domain-associated protein 6 isoform b [Homo sapiens] | 0 | 0 | 0 | 5 |
| gi\|8923271 | pre-mRNA-splicing factor CWC25 homolog [Homo sapiens] | 0 | 0 | 0 | 1 |
| gi\|57242777 | C-Myc-binding protein [Homo sapiens] | 0 | 0 | 0 | 4 |
| gi\|110611908 (+3) | mitogen-activated protein kinase kinase kinase kinase 1 isoform 1 [Homo sapiens] | 0 | 0 | 0 | 2 |
| gi\|29789367 (+9) | forkhead box protein P4 isoform 2 [Homo sapiens] | 0 | 0 | 0 | 1 |
| gi\|578815063 (+2) | PREDICTED: tyrosine-protein kinase SgK223 isoform X1 [Homo sapiens] | 0 | 0 | 0 | 2 |
| gi\|109809737 (+9) | general transcription factor IIH subunit 2-like protein [Homo sapiens] | 0 | 0 | 0 | 2 |
| gi\|11056016 (+2) | vasculin-like protein 1 [Homo sapiens] | 0 | 0 | 0 | 2 |
| gi\|226495417 (+1) | coiled-coil domain-containing protein 102A [Homo sapiens] | 0 | 0 | 0 | 1 |
| gi\|7706423 | U6 snRNA-associated Sm-like protein LSm7 [Homo sapiens] | 0 | 0 | 0 | 2 |
| gi\|156104891 | general transcription factor IIF subunit 1 [Homo sapiens] | 0 | 0 | 0 | 1 |
| gi\|34222389 (+5) | tRNA (uracil-5-)-methyltransferase homolog A isoform a [Homo sapiens] | 0 | 0 | 0 | 1 |
| gi\|264681556 (+3) | WD repeat-containing protein 26 isoform b [Homo sapiens] | 0 | 0 | 0 | 1 |
| gi\|4504201 | general transcription factor IIH subunit 4 [Homo sapiens] | 0 | 0 | 0 | 1 |
| gi\|578813666 (+8) | PREDICTED: ubiquitin carboxyl-terminal hydrolase 42 isoform X1 [Homo sapiens] | 0 | 0 | 0 | 3 |
| gi\|33356172 | nibrin [Homo sapiens] | 0 | 0 | 0 | 4 |
| gi\|194578909 | eukaryotic translation initiation factor 4E isoform 2 [Homo sapiens] | 0 | 0 | 0 | 2 |
| gi\|58761510 | yrdC domain-containing protein, mitochondrial precursor [Homo sapiens] | 0 | 0 | 0 | 3 |
| gi\|187608732 (+1) | c+1938:1979ellular nucleic acid-binding protein isoform 2 [Homo sapiens] | 0 | 0 | 0 | 1 |
| gi\|19923613 (+3) | enhancer of mRNA-decapping protein 3 [Homo sapiens] | 0 | 0 | 0 | 4 |
| gi\|31543452 (+15) | PX domain-containing protein kinase-like protein isoform 1 [Homo sapiens] | 0 | 0 | 0 | 1 |
| gi\|15011972 (+3) | rho guanine nucleotide exchange factor 1 isoform 2 [Homo sapiens] | 0 | 0 | 0 | 2 |
| gi\|530360311 (+3) | PREDICTED: agrin isoform X1 [Homo sapiens] | 0 | 0 | 0 | 1 |
| gi\|375493565 (+6) | ubiquitin carboxyl-terminal hydrolase 3 isoform 2 [Homo sapiens] | 0 | 0 | 0 | 1 |
| gi\|530380347 | PREDICTED: glucosamine-6-phosphate isomerase 1 isoform X2 [Homo sapiens] | 0 | 0 | 0 | 1 |
| gi\|108773808 | coiled-coil domain-containing protein 174 [Homo sapiens] | 0 | 0 | 0 | 2 |
| gi\|289577125 (+2) | transcription factor Sp3 isoform 2 [Homo sapiens] | 0 | 0 | 0 | 1 |
| gi\|530387601 (+1) | PREDICTED: cell division cycle-associated protein 2 isoform X6 [Homo sapiens] | 0 | 0 | 0 | 2 |
| gi\|5031635 | cofilin-1 [Homo sapiens] | 0 | 0 | 0 | 1 |
| gi\|4507909 (+1) | wiskott-Aldrich syndrome protein [Homo sapiens] | 0 | 0 | 0 | 1 |
| gi\|172073175 (+8) | serine/threonine-protein kinase greatwall isoform 2 [Homo sapiens] | 0 | 0 | 0 | 2 |
| g+A927:A1329i\|20127607 (+5) | spermatogenesis-defective protein 39 homolog isoform 1 [Homo sapiens] | 0 | 0 | 0 | 2 |
| gi\|40217847 | U5 small nuclear ribonucleoprotein 200 kDa helicase [Homo sapiens] | 0 | 1 | 7 | 18 |
| gi\|91208426 | pre-mRNA-processing-splicing factor 8 [Homo sapiens] | 0 | 1 | 6 | 18 |
| gi\|50726968 | microfibrillar-associated protein 1 [Homo sapiens] | 0 | 1 | 6 | 7 |
| gi\|68509926 | putative pre-mRNA-splicing factor ATP-dependent RNA helicase DHX15 [Homo sapiens] | 0 | 1 | 3 | 1 |
| gi\|5453597 | F-actin-capping protein subunit alpha-1 [Homo sapiens] | 0 | 1 | 2 | 0 |
| gi\|4826686 | ATP-dependent RNA helicase DDX1 [Homo sapiens] | 0 | 1 | 1 | 1 |
| gi\|4505939 | DNA-directed RNA polymerase II subunit RPB1 [Homo sapiens] | 0 | 1 | 1 | 1 |
| gi\|507834119 | ADP-ribosylation factor-like protein 6-interacting protein 4 isoform 1 [Homo sapiens] | 0 | 1 | 1 | 1 |
| gi\|5901926 | cleavage and polyadenylation specificity factor subunit 5 [Homo sapiens] | 0 | 1 | 1 | 5 |
| gi\|296010990 (+14) | abl interactor 1 isoform f [Homo sapiens] | 0 | 1 | 1 | 1 |
| gi\|24308127 (+1) | dnaJ homolog subfamily C member 10 isoform 1 precursor [Homo sapiens] | 0 | 1 | 1 | 0 |
| gi\|14249398 | PHD finger-like domain-containing protein 5A [Homo sapiens] | 0 | 1 | 1 | 2 |
| gi\|254553334 (+2) | DDB1- and CUL4-associated factor 11 isoform 1 [Homo sapiens] | 0 | 1 | 1 | 0 |
| gi\|304555581 (+17) | elongation factor 1-delta isoform 1 [Homo sapiens] | 0 | 1 | 1 | 1 |
| gi\|503774453 | proline-, glutamic acid- and leucine-rich protein 1 isoform 1 [Homo sapiens] | 0 | 1 | 1 | 2 |
| gi\|12667788 (+1) | myosin-9 [Homo sapiens] | 0 | 1 | 0 | 6 |
| gi\|578837205 | PREDICTED: DNA replication licensing factor MCM5 isoform X1 [Homo sapiens] | 0 | 1 | 0 | 1 |
| gi\|4506685 | 40S ribosomal protein S13 [Homo sapiens] | 0 | 1 | 0 | 1 |
| gi\|5031839 | keratin, type II cytoskeletal 6A [Homo sapiens] | 0 | 1 | 0 | 0 |
| gi\|4758950 | peptidyl-prolyl cis-trans isomerase B precursor [Homo sapiens] | 0 | 1 | 0 | 0 |
| gi\|4758788 | NADH dehydrogenase [ubiquinone] iron-sulfur protein 3, mitochondrial precursor [Homo sapiens] | 0 | 1 | 0 | 1 |
| gi\|54112121 | splicing factor 3B subunit 3 [Homo sapiens] | 0 | 1 | 0 | 1 |
| gi\|106775678 (+2) | histone H2A type 2-A [Homo sapiens] | 0 | 1 | 0 | 1 |
| gi\|46249393 (+1) | rho-related GTP-binding protein RhoG precursor [Homo sapiens] | 0 | 1 | 0 | 0 |
| gi\|19923475 | tRNA (adenine(58)-N(1))-methyltransferase non-catalytic subunit TRM6 isoform 1 [Homo sapiens] | 0 | 1 | 0 | 1 |
| gi\|221316723 | N-acetyltransferase 10 isoform a [Homo sapiens] | 0 | 1 | 0 | 1 |
| gi\|5803177 | beta-2-syntrophin [Homo sapiens] | 0 | 1 | 0 | 0 |
| gi\|215599015 (+1) | H/ACA ribonucleoprotein complex subunit 4 isoform 2 [Homo sapiens] | 0 | 1 | 0 | 0 |
| gi\|306482694 (+1) | serine/arginine-rich splicing factor 7 isoform 2 [Homo sapiens] | 0 | 1 | 0 | 1 |
| gi\|7705618 | 39S ribosomal protein L11, mitochondrial isoform a [Homo sapiens] | 0 | 1 | 0 | 0 |
| gi\|578836355 (+3) | PREDICTED: splicing factor U2AF 35 kDa subunit isoform X1 [Homo sapiens] | 0 | 1 | 0 | 0 |
| gi\|84043963 | eukaryotic translation initiation factor 5B [Homo sapiens] | 0 | 1 | 0 | 0 |
| gi\|15431295 (+1) | 60S ribosomal protein L13 isoform 1 [Homo sapiens] | 0 | 1 | 0 | 1 |
| gi\|197927454 (+1) | protein DEK isoform 2 [Homo sapiens] | 0 | 1 | 0 | 0 |
| gi\|189011566 (+1) | adenomatous polyposis coli protein isoform b [Homo sapiens] | 0 | 1 | 0 | 0 |
| gi\|4506901 | serine/arginine-rich splicing factor 3 [Homo sapiens] | 0 | 1 | 0 | 0 |
| gi\|20127499 | serine/arginine-rich splicing factor 6 [Homo sapiens] | 0 | 1 | 0 | 0 |
| gi\|21536320 (+12) | heterogeneous nuclear ribonucleoprotein U-like protein 1 isoform d [Homo sapiens] | 0 | 1 | 0 | 1 |
| gi\|8922534 | rRNA methyltransferase 3, mitochondrial [Homo sapiens] | 0 | 1 | 0 | 0 |
| gi\|21626466 (+3) | matrin-3 isoform a [Homo sapiens] | 0 | 1 | 0 | 1 |
| gi\|47419936 | SRSF protein kinase 1 [Homo sapiens] | 0 | 1 | 0 | 0 |
| gi\|193211616 | protein NipSnap homolog 1 isoform 1 [Homo sapiens] | 0 | 1 | 0 | 0 |
| gi\|7661936 | scaffold attachment factor B2 [Homo sapiens] | 0 | 1 | 0 | 0 |
| gi\|41322916 (+1) | plectin isoform 1 [Homo sapiens] | 0 | 1 | 0 | 0 |
| gi\|134142828 | mRNA-capping enzyme isoform a [Homo sapiens] | 0 | 1 | 0 | 0 |
| gi\|4507511 | metalloproteinase inhibitor 2 precursor [Homo sapiens] | 0 | 1 | 0 | 0 |
| gi\|767937781 | PREDICTED: leucine--tRNA ligase, cytoplasmic isoform X3 [Homo sapiens] | 0 | 1 | 0 | 0 |
| gi\|16950603 | 28S ribosomal protein S35, mitochondrial isoform 1 precursor [Homo sapiens] | 0 | 1 | 0 | 1 |
| gi\|33188463 | 28S ribosomal protein S9, mitochondrial [Homo sapiens] | 0 | 1 | 0 | 2 |
| gi\|4507129 | small nuclear ribonucleoprotein E isoform 1 [Homo sapiens] | 0 | 1 | 0 | 0 |
| gi\|24234747 (+1) | interleukin enhancer-binding factor 2 isoform 1 [Homo sapiens] | 0 | 1 | 0 | 0 |
| gi\|46276893 (+1) | transcription elongation factor B polypeptide 2 isoform b [Homo sapiens] | 0 | 1 | 0 | 0 |
| gi\|4506609 (+1) | 60S ribosomal protein L19 [Homo sapiens] | 0 | 1 | 0 | 0 |
| gi\|4507157 (+2) | sortilin-related receptor preproprotein [Homo sapiens] | 0 | 1 | 0 | 0 |
| gi\|4885381 | histone H1.5 [Homo sapiens] | 0 | 1 | 0 | 0 |
| gi\|578816078 (+5) | PREDICTED: integrator complex subunit 8 isoform X2 [Homo sapiens] | 0 | 1 | 0 | 0 |
| gi\|40254823 (+1) | phosphatidylinositol 3,4,5-trisphosphate 5-phosphatase 1 isoform b [Homo sapiens] | 0 | 1 | 0 | 0 |
| gi\|118722349 (+5) | RNA-binding protein 12B [Homo sapiens] | 0 | 1 | 0 | 0 |
| gi\|52632383 (+1) | heterogeneous nuclear ribonucleoprotein L isoform a [Homo sapiens] | 0 | 1 | 0 | 0 |
| gi\|767965608 | PREDICTED: F-box only protein 3 isoform X1 [Homo sapiens] | 0 | 1 | 0 | 0 |
| gi\|88758613 | complement component C1q receptor precursor [Homo sapiens] | 0 | 1 | 0 | 0 |
| gi\|530396224 (+2) | PREDICTED: splicing factor 3B subunit 2 isoform X1 [Homo sapiens] | 0 | 1 | 0 | 0 |
| gi\|226509740 (+2) | splicing factor, arginine/serine-rich 19 [Homo sapiens] | 0 | 1 | 0 | 0 |
| gi\|33356174 | pinin [Homo sapiens] | 0 | 1 | 0 | 0 |
| gi\|33356163 (+2) | eukaryotic translation initiation factor 1A, Y-chromosomal isoform 1 [Homo sapiens] | 0 | 1 | 0 | 0 |
| gi\|4506699 | 40S ribosomal protein S21 [Homo sapiens] | 0 | 1 | 0 | 0 |
| gi\|4506715 | 40S ribosomal protein S28 [Homo sapiens] | 0 | 1 | 0 | 0 |
| gi\|14110414 (+3) | heterogeneous nuclear ribonucleoprotein D0 isoform c [Homo sapiens] | 0 | 1 | 0 | 0 |
| gi\|27436917 (+4) | misshapen-like kinase 1 isoform 3 [Homo sapiens] | 0 | 1 | 0 | 2 |
| gi\|187960109 (+2) | RNA-binding protein 28 isoform 1 [Homo sapiens] | 0 | 1 | 0 | 0 |
| gi\|148747866 | heparan-sulfate 6-O-sulfotransferase 1 [Homo sapiens] | 0 | 1 | 0 | 0 |
| gi\|269847776 (+1) | Abelson tyrosine-protein kinase 2 isoform f [Homo sapiens] | 0 | 1 | 0 | 2 |
| gi\|150417986 (+1) | brefeldin A-inhibited guanine nucleotide-exchange protein 2 [Homo sapiens] | 0 | 1 | 0 | 0 |
| gi\|16418361 (+4) | protein RFT1 homolog [Homo sapiens] | 0 | 1 | 0 | 0 |
| gi\|21361478 (+8) | septin-6 isoform B [Homo sapiens] | 0 | 1 | 0 | 0 |
| gi\|24430139 (+1) | RNA polymerase II-associated protein 1 [Homo sapiens] | 0 | 1 | 0 | 0 |
| gi\|118918395 (+3) | fibronectin type-III domain-containing protein 3A isoform 1 [Homo sapiens] | 0 | 1 | 0 | 2 |
| gi\|5031953 | dol-P-Man:Man(5)GlcNAc(2)-PP-Dol alpha-1,3-mannosyltransferase isoform a [Homo sapiens] | 0 | 1 | 0 | 0 |
| gi\|33356550 (+2) | G patch domain-containing protein 4 isoform 2 [Homo sapiens] | 0 | 1 | 0 | 0 |
| gi\|13443000 (+1) | F-box only protein 22 isoform b [Homo sapiens] | 0 | 1 | 0 | 0 |
| gi\|768002572 | PREDICTED: NADH dehydrogenase [ubiquinone] 1 beta subcomplex subunit 7 isoform X1 [Homo sapiens] | 0 | 1 | 0 | 0 |
| gi\|14165464 (+4) | polypyrimidine tract-binding protein 1 isoform b [Homo sapiens] | 0 | 1 | 0 | 0 |
| gi\|530361637 (+1) | PREDICTED: splicing factor 3A subunit 3 isoform X1 [Homo sapiens] | 0 | 1 | 0 | 0 |
| gi\|291190787 (+1) | E3 ubiquitin-protein ligase MYCBP2 [Homo sapiens] | 0 | 2 | 16 | 30 |
| gi\|4506625 | 60S ribosomal protein L27a [Homo sapiens] | 0 | 2 | 2 | 2 |
| gi\|530360783 (+4) | PREDICTED: serine/arginine repetitive matrix protein 1 isoform X1 [Homo sapiens] | 0 | 2 | 2 | 2 |
| gi\|4506753 | ruvB-like 1 [Homo sapiens] | 0 | 2 | 1 | 0 |
| gi\|217330646 (+2) | activated RNA polymerase II transcriptional coactivator p15 [Homo sapiens] | 0 | 2 | 1 | 2 |
| gi\|208431833 (+2) | polyadenylate-binding protein 4 isoform 1 [Homo sapiens] | 0 | 2 | 1 | 1 |
| gi\|119395754 | keratin, type II cytoskeletal 5 [Homo sapiens] | 0 | 2 | 0 | 1 |
| gi\|46367787 (+1) | polyadenylate-binding protein 1 [Homo sapiens] | 0 | 2 | 0 | 1 |
| gi\|4506583 | replication protein A 70 kDa DNA-binding subunit [Homo sapiens] | 0 | 2 | 0 | 0 |
| gi\|11415026 | 60S ribosomal protein L18a [Homo sapiens] | 0 | 2 | 0 | 1 |
| gi\|4506741 | 40S ribosomal protein S7 [Homo sapiens] | 0 | 2 | 0 | 2 |
| gi\|4506613 | 60S ribosomal protein L22 proprotein [Homo sapiens] | 0 | 2 | 0 | 1 |
| gi\|530373069 | PREDICTED: DNA topoisomerase 2-beta isoform X1 [Homo sapiens] | 0 | 2 | 0 | 0 |
| gi\|4502491 | complement component 1 Q subcomponent-binding protein, mitochondrial precursor [Homo sapiens] | 0 | 2 | 0 | 1 |
| gi\|40254924 | leucine-rich repeat-containing protein 59 [Homo sapiens] | 0 | 2 | 0 | 0 |
| gi\|441478305 (+1) | glutamine--tRNA ligase isoform b [Homo sapiens] | 0 | 2 | 0 | 0 |
| gi\|56699482 | protein FAM98A isoform 1 [Homo sapiens] | 0 | 2 | 0 | 0 |
| gi\|4506633 | 60S ribosomal protein L31 isoform 1 [Homo sapiens] | 0 | 2 | 0 | 0 |
| gi\|38327634 | ATP-dependent RNA helicase DDX18 [Homo sapiens] | 0 | 2 | 0 | 1 |
| gi\|530402322 (+1) | PREDICTED: high mobility group protein B1 isoform X2 [Homo sapiens] | 0 | 2 | 0 | 0 |
| gi\|149999611 | signal recognition particle 14 kDa protein [Homo sapiens] | 0 | 2 | 0 | 0 |
| gi\|14110407 (+1) | heterogeneous nuclear ribonucleoprotein D-like isoform a [Homo sapiens] | 0 | 2 | 0 | 1 |
| gi\|291045198 (+3) | protein cereblon isoform 2 [Homo sapiens] | 0 | 2 | 0 | 0 |
| gi\|4504341 | histone acetyltransferase type B catalytic subunit [Homo sapiens] | 0 | 2 | 0 | 1 |
| gi\|21735417 (+1) | protein bicaudal D homolog 2 isoform 2 [Homo sapiens] | 0 | 2 | 0 | 0 |
| gi\|320461686 (+1) | TRMT1-like protein isoform 2 [Homo sapiens] | 0 | 2 | 0 | 0 |
| gi\|57864582 | hornerin [Homo sapiens] | 0 | 2 | 0 | 0 |
| gi\|55956921 | heterogeneous nuclear ribonucleoprotein A/B isoform b [Homo sapiens] | 0 | 2 | 0 | 2 |
| gi\|210032390 (+1) | protein SEC13 homolog isoform 2 [Homo sapiens] | 0 | 2 | 0 | 0 |
| gi\|578817539 (+1) | PREDICTED: 28S ribosomal protein S2, mitochondrial isoform X1 [Homo sapiens] | 0 | 2 | 0 | 0 |
| gi\|225007648 (+2) | ribonucleases P/MRP protein subunit POP1 [Homo sapiens] | 0 | 2 | 0 | 0 |
| gi\|149193321 (+1) | G-rich sequence factor 1 isoform 1 [Homo sapiens] | 0 | 2 | 0 | 0 |
| gi\|237649012 (+1) | probable rRNA-processing protein EBP2 isoform 1 [Homo sapiens] | 0 | 2 | 0 | 0 |
| gi\|239049440 (+10) | FCH domain only protein 1 isoform a [Homo sapiens] | 0 | 2 | 0 | 1 |
| gi\|30348954 | E3 ubiquitin-protein ligase MIB1 [Homo sapiens] | 0 | 3 | 4 | 7 |
| gi\|208973238 (+9) | 14-3-3 protein zeta/delta [Homo sapiens] | 0 | 3 | 2 | 1 |
| gi\|530393314 (+3) | PREDICTED: protein RRP5 homolog isoform X2 [Homo sapiens] | 0 | 3 | 1 | 1 |
| gi\|7662645 | 28S ribosomal protein S18b, mitochondrial [Homo sapiens] | 0 | 3 | 1 | 1 |
| gi\|18490987 | mRNA turnover protein 4 homolog [Homo sapiens] | 0 | 3 | 1 | 3 |
| gi\|14670350 (+1) | general transcription factor II-I isoform 1 [Homo sapiens] | 0 | 3 | 0 | 3 |
| gi\|4885377 | histone H1.3 [Homo sapiens] | 0 | 3 | 0 | 0 |
| gi\|19913406 (+1) | DNA topoisomerase 2-alpha [Homo sapiens] | 0 | 3 | 0 | 1 |
| gi\|4506661 | 60S ribosomal protein L7a [Homo sapiens] | 0 | 3 | 0 | 1 |
| gi\|320461711 (+3) | peroxiredoxin-1 [Homo sapiens] | 0 | 3 | 0 | 0 |
| gi\|14591909 | 60S ribosomal protein L5 [Homo sapiens] | 0 | 3 | 0 | 2 |
| gi\|117190174 (+3) | heterogeneous nuclear ribonucleoproteins C1/C2 isoform b [Homo sapiens] | 0 | 3 | 0 | 1 |
| gi\|5031815 | lysine--tRNA ligase isoform 2 [Homo sapiens] | 0 | 3 | 0 | 0 |
| gi\|545478466 (+2) | septin-2 isoform b [Homo sapiens] | 0 | 3 | 0 | 0 |
| gi\|91718899 | mitogen-activated protein kinase 3 isoform 1 [Homo sapiens] | 0 | 3 | 0 | 0 |
| gi\|116812610 (+3) | MICOS complex subunit MIC27 precursor [Homo sapiens] | 0 | 3 | 0 | 0 |
| gi\|4505163 | alpha-1,6-mannosyl-glycoprotein 2-beta-N-acetylglucosaminyltransferase [Homo sapiens] | 0 | 3 | 0 | 0 |
| gi\|4505813 (+2) | dynein light chain 1, cytoplasmic [Homo sapiens] | 0 | 3 | 0 | 0 |
| gi\|24497453 (+1) | nuclear pore complex protein Nup88 [Homo sapiens] | 0 | 3 | 0 | 0 |
| gi\|4507217 | signal recognition particle 9 kDa protein isoform 2 [Homo sapiens] | 0 | 3 | 0 | 0 |
| gi\|12408675 (+1) | prefoldin subunit 2 [Homo sapiens] | 0 | 3 | 0 | 0 |
| gi\|4759344 | centromere/kinetochore protein zw10 homolog [Homo sapiens] | 0 | 3 | 0 | 0 |
| gi\|55769587 (+2) | nucleolar protein 14 isoform 1 [Homo sapiens] | 0 | 3 | 0 | 0 |
| gi\|530402180 (+4) | PREDICTED: sister chromatid cohesion protein PDS5 homolog B isoform X3 [Homo sapiens] | 0 | 3 | 0 | 1 |
| gi\|17105394 | 60S ribosomal protein L23a [Homo sapiens] | 0 | 4 | 1 | 1 |
| gi\|4506631 | 60S ribosomal protein L30 [Homo sapiens] | 0 | 4 | 0 | 3 |
| gi\|747165389 (+1) | 40S ribosomal protein SA isoform 2 [Homo sapiens] | 0 | 4 | 0 | 0 |
| gi\|4505227 | myeloid cell nuclear differentiation antigen [Homo sapiens] | 0 | 4 | 0 | 0 |
| gi\|34740329 (+2) | heterogeneous nuclear ribonucleoprotein A3 [Homo sapiens] | 0 | 4 | 0 | 2 |
| gi\|118498359 | ribosomal L1 domain-containing protein 1 [Homo sapiens] | 0 | 4 | 0 | 0 |
| gi\|60279268 (+1) | splicing factor U2AF 65 kDa subunit isoform b [Homo sapiens] | 0 | 4 | 0 | 0 |
| gi\|148352329 (+4) | septin-7 isoform 2 [Homo sapiens] | 0 | 4 | 0 | 0 |
| gi\|767904524 | PREDICTED: heterochromatin protein 1-binding protein 3 isoform X3 [Homo sapiens] | 0 | 4 | 0 | 0 |
| gi\|4506669 | 60S acidic ribosomal protein P1 isoform 1 [Homo sapiens] | 0 | 4 | 0 | 1 |
| gi\|10835067 (+1) | lupus La protein [Homo sapiens] | 0 | 5 | 1 | 1 |
| gi\|16579885 | 60S ribosomal protein L4 [Homo sapiens] | 0 | 5 | 0 | 2 |
| gi\|15431301 | 60S ribosomal protein L7 [Homo sapiens] | 0 | 5 | 0 | 0 |
| gi\|228008291 (+6) | heterogeneous nuclear ribonucleoprotein Q isoform 1 [Homo sapiens] | 0 | 5 | 0 | 0 |
| gi\|4557809 (+3) | ornithine aminotransferase, mitochondrial isoform 1 precursor [Homo sapiens] | 0 | 5 | 0 | 0 |
| gi\|216548145 (+1) | RNA pseudouridylate synthase domain-containing protein 3 isoform 1 [Homo sapiens] | 0 | 5 | 0 | 0 |
| gi\|4506671 | 60S acidic ribosomal protein P2 [Homo sapiens] | 0 | 6 | 4 | 4 |
| gi\|10863945 | X-ray repair cross-complementing protein 5 [Homo sapiens] | 0 | 6 | 0 | 0 |
| gi\|16753227 (+6) | 60S ribosomal protein L6 [Homo sapiens] | 0 | 7 | 0 | 1 |
| gi\|20986531 (+1) | mitogen-activated protein kinase 1 [Homo sapiens] | 0 | 7 | 0 | 0 |
| gi\|212549553 (+3) | interleukin enhancer-binding factor 3 isoform d [Homo sapiens] | 0 | 7 | 0 | 0 |
| gi\|47132620 | keratin, type II cytoskeletal 2 epidermal [Homo sapiens] | 0 | 8 | 3 | 8 |
| gi\|767943525 | PREDICTED: utrophin isoform X4 [Homo sapiens] | 0 | 8 | 0 | 0 |
| gi\|16933546 (+1) | 60S acidic ribosomal protein P0 [Homo sapiens] | 0 | 10 | 0 | 1 |
| gi\|550544217 (+1) | rapamycin-insensitive companion of mTOR isoform 2 [Homo sapiens] | 0 | 11 | 2 | 3 |
| gi\|50659095 | nucleolar RNA helicase 2 isoform 1 [Homo sapiens] | 0 | 11 | 0 | 0 |
| gi\|4503841 (+1) | X-ray repair cross-complementing protein 6 isoform 1 [Homo sapiens] | 0 | 11 | 0 | 0 |
| gi\|768037772 | PREDICTED: moesin isoform X3 [Homo sapiens] | 0 | 14 | 0 | 0 |
| gi\|62868213 (+1) | Golgi-associated PDZ and coiled-coil motif-containing protein isoform b [Homo sapiens] | 0 | 15 | 0 | 0 |
| gi\|319655556 (+6) | coiled-coil domain-containing protein 136 isoform 1 [Homo sapiens] | 0 | 19 | 0 | 0 |
| gi\|161702986 (+1) | ezrin [Homo sapiens] | 0 | 20 | 0 | 0 |
| gi\|4759140 | Na(+)/H(+) exchange regulatory cofactor NHE-RF1 [Homo sapiens] | 0 | 33 | 0 | 0 |
| gi\|359718912 | probable E3 ubiquitin-protein ligase HECTD4 [Homo sapiens] | 0 | 47 | 0 | 0 |
| gi\|27436946 | lamin isoform A [Homo sapiens] | 1 | 0 | 6 | 8 |
| gi\|192807334 (+4) | serrate RNA effector molecule homolog isoform d [Homo sapiens] | 1 | 0 | 4 | 6 |
| gi\|767944712 | PREDICTED: DNA-binding protein Ikaros isoform X5 [Homo sapiens] | 1 | 0 | 4 | 3 |
| gi\|530410915 (+3) | PREDICTED: schlafen family member 13 isoform X1 [Homo sapiens] | 1 | 0 | 4 | 2 |
| gi\|768009682 (+1) | PREDICTED: glutaminyl-peptide cyclotransferase-like protein isoform X1 [Homo sapiens] | 1 | 0 | 3 | 2 |
| gi\|102469034 (+3) | tyrosine-protein kinase JAK1 [Homo sapiens] | 1 | 0 | 3 | 0 |
| gi\|767908955 | PREDICTED: torsin-1A-interacting protein 1 isoform X2 [Homo sapiens] | 1 | 0 | 3 | 8 |
| gi\|302699237 (+7) | eukaryotic translation initiation factor 4 gamma 1 isoform 1 [Homo sapiens] | 1 | 0 | 3 | 18 |
| gi\|145580615 | protein KRI1 homolog [Homo sapiens] | 1 | 0 | 3 | 3 |
| gi\|110347463 | transcription factor HIVEP2 [Homo sapiens] | 1 | 0 | 3 | 2 |
| gi\|514052677 (+3) | zinc finger protein 316 [Homo sapiens] | 1 | 0 | 3 | 1 |
| gi\|32528306 (+2) | replication factor C subunit 1 isoform 1 [Homo sapiens] | 1 | 0 | 2 | 3 |
| gi\|4505781 (+3) | phosphorylase b kinase regulatory subunit alpha, liver isoform [Homo sapiens] | 1 | 0 | 2 | 0 |
| gi\|767948252 (+1) | PREDICTED: acylglycerol kinase, mitochondrial isoform X1 [Homo sapiens] | 1 | 0 | 2 | 1 |
| gi\|4759158 (+1) | small nuclear ribonucleoprotein Sm D2 isoform 1 [Homo sapiens] | 1 | 0 | 2 | 1 |
| gi\|256223453 | probable ATP-dependent RNA helicase DDX20 [Homo sapiens] | 1 | 0 | 2 | 2 |
| gi\|25777602 | 26S proteasome non-ATPase regulatory subunit 2 isoform 1 [Homo sapiens] | 1 | 0 | 2 | 1 |
| gi\|21071052 (+5) | helicase-like transcription factor [Homo sapiens] | 1 | 0 | 2 | 0 |
| gi\|295821207 (+5) | cell division control protein 45 homolog isoform 3 [Homo sapiens] | 1 | 0 | 2 | 2 |
| gi\|20149619 (+2) | dehydrogenase/reductase SDR family member 7B [Homo sapiens] | 1 | 0 | 2 | 0 |
| gi\|15011918 | renin receptor precursor [Homo sapiens] | 1 | 0 | 2 | 0 |
| gi\|390131986 (+3) | sentrin-specific protease 1 [Homo sapiens] | 1 | 0 | 2 | 1 |
| gi\|28872725 (+1) | 26S proteasome non-ATPase regulatory subunit 11 [Homo sapiens] | 1 | 0 | 2 | 0 |
| gi\|4502205 | ADP-ribosylation factor 4 [Homo sapiens] | 1 | 0 | 1 | 0 |
| gi\|374671775 (+4) | single-stranded DNA-binding protein, mitochondrial precursor [Homo sapiens] | 1 | 0 | 1 | 2 |
| gi\|530423974 (+2) | PREDICTED: phosphorylase b kinase regulatory subunit beta isoform X2 [Homo sapiens] | 1 | 0 | 1 | 0 |
| gi\|5031569 | alpha-centractin [Homo sapiens] | 1 | 0 | 1 | 0 |
| gi\|586597929 (+1) | antigen peptide transporter 2 isoform 3 [Homo sapiens] | 1 | 0 | 1 | 0 |
| gi\|38202257 (+2) | neutral alpha-glucosidase AB isoform 2 precursor [Homo sapiens] | 1 | 0 | 1 | 0 |
| gi\|4506209 | 26S protease regulatory subunit 7 isoform 1 [Homo sapiens] | 1 | 0 | 1 | 3 |
| gi\|94721261 | 2',3'-cyclic-nucleotide 3'-phosphodiesterase [Homo sapiens] | 1 | 0 | 1 | 1 |
| gi\|18644883 (+5) | ATP synthase-coupling factor 6, mitochondrial isoform a precursor [Homo sapiens] | 1 | 0 | 1 | 0 |
| gi\|746817424 (+3) | 60S ribosomal protein L10 isoform a [Homo sapiens] | 1 | 0 | 1 | 2 |
| gi\|118640875 (+3) | linker for activation of T-cells family member 2 precursor [Homo sapiens] | 1 | 0 | 1 | 1 |
| gi\|11141903 | FAST kinase domain-containing protein 5 [Homo sapiens] | 1 | 0 | 1 | 0 |
| gi\|768003080 | PREDICTED: tRNA (guanine(26)-N(2))-dimethyltransferase isoform X2 [Homo sapiens] | 1 | 0 | 1 | 2 |
| gi\|239582755 | unconventional myosin-Ig [Homo sapiens] | 1 | 0 | 1 | 1 |
| gi\|331284125 | E1A-binding protein p400 [Homo sapiens] | 1 | 0 | 1 | 1 |
| gi\|4503529 | eukaryotic initiation factor 4A-I isoform 1 [Homo sapiens] | 1 | 0 | 1 | 4 |
| gi\|260099723 (+1) | L-lactate dehydrogenase A chain isoform 3 [Homo sapiens] | 1 | 0 | 1 | 1 |
| gi\|209869993 (+6) | trifunctional purine biosynthetic protein adenosine-3 isoform 1 [Homo sapiens] | 1 | 0 | 1 | 0 |
| gi\|34147558 (+5) | protein lyl-1 [Homo sapiens] | 1 | 0 | 1 | 2 |
| gi\|4505881 | plasminogen isoform 1 precursor [Homo sapiens] | 1 | 0 | 1 | 1 |
| gi\|5729991 | 26S protease regulatory subunit 6B isoform 1 [Homo sapiens] | 1 | 0 | 1 | 0 |
| gi\|109689718 (+4) | monoacylglycerol lipase ABHD12 isoform a [Homo sapiens] | 1 | 0 | 1 | 1 |
| gi\|24797086 (+6) | importin-5 [Homo sapiens] | 1 | 0 | 1 | 0 |
| gi\|21361454 (+1) | pyrroline-5-carboxylate reductase 2 isoform 1 [Homo sapiens] | 1 | 0 | 1 | 0 |
| gi\|253970504 (+9) | RNA-binding protein EWS isoform 4 [Homo sapiens] | 1 | 0 | 1 | 1 |
| gi\|256773260 | mitochondrial import inner membrane translocase subunit Tim8 B [Homo sapiens] | 1 | 0 | 1 | 1 |
| gi\|6005717 | ATP synthase subunit e, mitochondrial [Homo sapiens] | 1 | 0 | 1 | 0 |
| gi\|67782362 | ATP-dependent RNA helicase DHX29 [Homo sapiens] | 1 | 0 | 1 | 0 |
| gi\|10937869 (+11) | survival motor neuron protein isoform d [Homo sapiens] | 1 | 0 | 1 | 0 |
| gi\|4502281 | sodium/potassium-transporting ATPase subunit beta-3 [Homo sapiens] | 1 | 0 | 1 | 0 |
| gi\|767942652 | PREDICTED: sentrin-specific protease 6 isoform X4 [Homo sapiens] | 1 | 0 | 1 | 2 |
| gi\|133925811 (+3) | transportin-1 isoform 1 [Homo sapiens] | 1 | 0 | 1 | 0 |
| gi\|191252801 (+4) | WD repeat- and FYVE domain-containing protein 4 [Homo sapiens] | 1 | 0 | 0 | 0 |
| gi\|32483374 | nucleolar protein 56 [Homo sapiens] | 1 | 0 | 0 | 0 |
| gi\|149999606 (+1) | mannosyl-oligosaccharide glucosidase isoform 1 [Homo sapiens] | 1 | 0 | 0 | 0 |
| gi\|153791313 (+3) | protein SCO2 homolog, mitochondrial precursor [Homo sapiens] | 1 | 0 | 0 | 1 |
| gi\|578801702 | PREDICTED: rho guanine nucleotide exchange factor 2 isoform X10 [Homo sapiens] | 1 | 0 | 0 | 0 |
| gi\|322303127 (+2) | 40S ribosomal protein S10 [Homo sapiens] | 1 | 0 | 0 | 0 |
| gi\|7706563 | ras-related protein Rab-8B [Homo sapiens] | 1 | 0 | 0 | 3 |
| gi\|54607135 | mitochondrial import receptor subunit TOM70 [Homo sapiens] | 1 | 0 | 0 | 0 |
| gi\|51944953 | nodal modulator 1 precursor [Homo sapiens] | 1 | 0 | 0 | 1 |
| gi\|169404009 | translocon-associated protein subunit alpha isoform 1 precursor [Homo sapiens] | 1 | 0 | 0 | 1 |
| gi\|4505357 | cytochrome c oxidase subunit NDUFA4 [Homo sapiens] | 1 | 0 | 0 | 1 |
| gi\|21361114 (+1) | mitochondrial 2-oxoglutarate/malate carrier protein isoform 1 [Homo sapiens] | 1 | 0 | 0 | 0 |
| gi\|5031977 (+1) | nicotinamide phosphoribosyltransferase precursor [Homo sapiens] | 1 | 0 | 0 | 0 |
| gi\|4506605 | 60S ribosomal protein L23 [Homo sapiens] | 1 | 0 | 0 | 1 |
| gi\|4505773 (+2) | prohibitin isoform 1 [Homo sapiens] | 1 | 0 | 0 | 1 |
| gi\|5032093 | neutral amino acid transporter B(0) isoform 1 [Homo sapiens] | 1 | 0 | 0 | 0 |
| gi\|39725634 | la-related protein 1 [Homo sapiens] | 1 | 0 | 0 | 1 |
| gi\|32307144 (+2) | procollagen-lysine,2-oxoglutarate 5-dioxygenase 1 precursor [Homo sapiens] | 1 | 0 | 0 | 1 |
| gi\|42794752 (+1) | long-chain-fatty-acid--CoA ligase 3 [Homo sapiens] | 1 | 0 | 0 | 0 |
| gi\|239787838 (+1) | testis-expressed sequence 10 protein isoform 1 [Homo sapiens] | 1 | 0 | 0 | 0 |
| gi\|195976805 (+11) | hypoxia up-regulated protein 1 precursor [Homo sapiens] | 1 | 0 | 0 | 0 |
| gi\|13375618 | delta(24)-sterol reductase precursor [Homo sapiens] | 1 | 0 | 0 | 1 |
| gi\|4505067 | mitotic spindle assembly checkpoint protein MAD2A [Homo sapiens] | 1 | 0 | 0 | 2 |
| gi\|4885281 | glutamate dehydrogenase 1, mitochondrial precursor [Homo sapiens] | 1 | 0 | 0 | 0 |
| gi\|50658063 (+3) | structural maintenance of chromosomes protein 4 isoform 1 [Homo sapiens] | 1 | 0 | 0 | 0 |
| gi\|13128860 | histone deacetylase 1 [Homo sapiens] | 1 | 0 | 0 | 1 |
| gi\|530372055 (+1) | PREDICTED: Fanconi anemia group D2 protein isoform X2 [Homo sapiens] | 1 | 0 | 0 | 0 |
| gi\|4506681 | 40S ribosomal protein S11 [Homo sapiens] | 1 | 0 | 0 | 1 |
| gi\|15431293 (+5) | 60S ribosomal protein L15 isoform 1 [Homo sapiens] | 1 | 0 | 0 | 1 |
| gi\|7661672 | polymerase delta-interacting protein 2 isoform 1 [Homo sapiens] | 1 | 0 | 0 | 1 |
| gi\|214830079 (+9) | spartin [Homo sapiens] | 1 | 0 | 0 | 0 |
| gi\|4503107 (+1) | cystatin-C precursor [Homo sapiens] | 1 | 0 | 0 | 0 |
| gi\|4503549 (+3) | neutrophil elastase preproprotein [Homo sapiens] | 1 | 0 | 0 | 0 |
| gi\|183603929 (+2) | serine/threonine-protein phosphatase 6 catalytic subunit isoform a [Homo sapiens] | 1 | 0 | 0 | 0 |
| gi\|20270303 (+1) | mitochondrial Rho GTPase 2 [Homo sapiens] | 1 | 0 | 0 | 0 |
| gi\|530393090 | PREDICTED: cytochrome c oxidase assembly protein COX15 homolog isoform X1 [Homo sapiens] | 1 | 0 | 0 | 0 |
| gi\|767948267 | PREDICTED: solute carrier family 25 member 40 isoform X2 [Homo sapiens] | 1 | 0 | 0 | 0 |
| gi\|45580709 | protein unc-93 homolog B1 [Homo sapiens] | 1 | 0 | 0 | 1 |
| gi\|34147522 (+5) | chaperone activity of bc1 complex-like, mitochondrial [Homo sapiens] | 1 | 0 | 0 | 0 |
| gi\|4502643 | T-complex protein 1 subunit zeta isoform a [Homo sapiens] | 1 | 0 | 0 | 0 |
| gi\|17136080 (+1) | peroxisomal biogenesis factor 16 isoform 2 [Homo sapiens] | 1 | 0 | 0 | 1 |
| gi\|4506787 | ras GTPase-activating-like protein IQGAP1 [Homo sapiens] | 1 | 0 | 0 | 0 |
| gi\|5454122 | mitochondrial import inner membrane translocase subunit Tim23 [Homo sapiens] | 1 | 0 | 0 | 0 |
| gi\|380837121 | vesicle-trafficking protein SEC22b precursor [Homo sapiens] | 1 | 0 | 0 | 0 |
| gi\|221316645 | ubiquitin-associated domain-containing protein 2 isoform 1 precursor [Homo sapiens] | 1 | 0 | 0 | 1 |
| gi\|4503175 (+1) | C-X-C chemokine receptor type 4 isoform b [Homo sapiens] | 1 | 0 | 0 | 0 |
| gi\|7705855 | estradiol 17-beta-dehydrogenase 12 [Homo sapiens] | 1 | 0 | 0 | 0 |
| gi\|34147498 | transmembrane protein 70, mitochondrial isoform a [Homo sapiens] | 1 | 0 | 0 | 0 |
| gi\|13376840 (+2) | WD repeat-containing protein 61 isoform a [Homo sapiens] | 1 | 0 | 0 | 0 |
| gi\|54292123 (+6) | lysosomal-trafficking regulator [Homo sapiens] | 1 | 0 | 0 | 0 |
| gi\|61743952 | 1-acyl-sn-glycerol-3-phosphate acyltransferase epsilon [Homo sapiens] | 1 | 0 | 0 | 0 |
| gi\|7019485 | programmed cell death protein 6 isoform 1 [Homo sapiens] | 1 | 0 | 0 | 0 |
| gi\|8922601 | ADP-ribosylation factor-like protein 8B [Homo sapiens] | 1 | 0 | 0 | 0 |
| gi\|222136639 | C-1-tetrahydrofolate synthase, cytoplasmic [Homo sapiens] | 1 | 0 | 0 | 0 |
| gi\|94536771 | cytochrome c oxidase assembly factor 3 homolog, mitochondrial [Homo sapiens] | 1 | 0 | 0 | 0 |
| gi\|38016957 | rho-related GTP-binding protein RhoF precursor [Homo sapiens] | 1 | 0 | 0 | 0 |
| gi\|530408425 (+1) | PREDICTED: putative RNA-binding protein Luc7-like 1 isoform X1 [Homo sapiens] | 1 | 0 | 0 | 1 |
| gi\|7706495 | dnaJ homolog subfamily B member 11 precursor [Homo sapiens] | 1 | 0 | 0 | 0 |
| gi\|51479156 | ATP synthase subunit g, mitochondrial [Homo sapiens] | 1 | 0 | 0 | 0 |
| gi\|29826282 | protein phosphatase 1G [Homo sapiens] | 1 | 0 | 0 | 0 |
| gi\|530370575 (+3) | PREDICTED: telomere-associated protein RIF1 isoform X1 [Homo sapiens] | 1 | 0 | 0 | 0 |
| gi\|197100773 | probable arginine--tRNA ligase, mitochondrial precursor [Homo sapiens] | 1 | 0 | 0 | 0 |
| gi\|82546830 | exocyst complex component 4 isoform a [Homo sapiens] | 1 | 0 | 0 | 0 |
| gi\|282396088 | cytochrome c oxidase assembly factor 7 [Homo sapiens] | 1 | 0 | 0 | 3 |
| gi\|19923264 (+9) | ras-related protein Rab-27A [Homo sapiens] | 1 | 0 | 0 | 0 |
| gi\|530421012 (+2) | PREDICTED: acyl-coenzyme A thioesterase 9, mitochondrial isoform X1 [Homo sapiens] | 1 | 0 | 0 | 0 |
| gi\|13489093 (+1) | elongation of very long chain fatty acids protein 1 isoform 1 [Homo sapiens] | 1 | 0 | 0 | 0 |
| gi\|296317337 (+4) | voltage-dependent anion-selective channel protein 2 isoform 1 [Homo sapiens] | 1 | 0 | 0 | 0 |
| gi\|27477134 | nuclear pore membrane glycoprotein 210 precursor [Homo sapiens] | 1 | 0 | 0 | 0 |
| gi\|767995545 | PREDICTED: HEAT repeat-containing protein 6 isoform X2 [Homo sapiens] | 1 | 0 | 0 | 0 |
| gi\|166295192 (+2) | serine/threonine-protein kinase Chk1 isoform 1 [Homo sapiens] | 1 | 0 | 0 | 0 |
| gi\|131412225 | keratin, type I cytoskeletal 13 isoform a [Homo sapiens] | 1 | 0 | 0 | 0 |
| gi\|251831110 | cytochrome c oxidase subunit II (mitochondrion) [Homo sapiens] | 1 | 0 | 0 | 1 |
| gi\|45827701 (+1) | protein dopey-2 [Homo sapiens] | 1 | 0 | 0 | 0 |
| gi\|33946291 (+4) | lysophosphatidylcholine acyltransferase 1 [Homo sapiens] | 1 | 0 | 0 | 0 |
| gi\|196049380 (+4) | 3-hydroxy-3-methylglutaryl-Coenzyme A reductase isoform 2 [Homo sapiens] | 1 | 0 | 0 | 0 |
| gi\|14249554 | SPRY domain-containing protein 3 [Homo sapiens] | 1 | 0 | 0 | 0 |
| gi\|14042927 (+4) | MLN64 N-terminal domain homolog [Homo sapiens] | 1 | 0 | 0 | 0 |
| gi\|109698595 (+2) | trafficking protein particle complex subunit 5 [Homo sapiens] | 1 | 0 | 0 | 0 |
| gi\|20149675 | EF-hand domain-containing protein D2 [Homo sapiens] | 1 | 0 | 0 | 0 |
| gi\|767974366 (+1) | PREDICTED: nucleosome assembly protein 1-like 1 isoform X4 [Homo sapiens] | 1 | 0 | 0 | 0 |
| gi\|221136993 (+1) | thioredoxin-related transmembrane protein 2 isoform 2 [Homo sapiens] | 1 | 0 | 0 | 0 |
| gi\|4502227 | ADP-ribosylation factor-like protein 1 isoform 1 [Homo sapiens] | 1 | 0 | 0 | 0 |
| gi\|46852178 | E3 ubiquitin-protein ligase KCMF1 [Homo sapiens] | 1 | 0 | 0 | 0 |
| gi\|767930274 | PREDICTED: sister chromatid cohesion protein PDS5 homolog A isoform X3 [Homo sapiens] | 1 | 0 | 0 | 1 |
| gi\|124028525 | phenylalanine--tRNA ligase beta subunit [Homo sapiens] | 1 | 0 | 0 | 0 |
| gi\|151101459 | serine/threonine-protein phosphatase 6 regulatory subunit 1 [Homo sapiens] | 1 | 0 | 0 | 0 |
| gi\|5729875 | membrane-associated progesterone receptor component 1 isoform 1 [Homo sapiens] | 1 | 0 | 0 | 1 |
| gi\|426214088 (+1) | reticulocalbin-2 isoform b precursor [Homo sapiens] | 1 | 0 | 0 | 1 |
| gi\|768003035 (+1) | PREDICTED: transmembrane protein 161A isoform X1 [Homo sapiens] | 1 | 0 | 0 | 0 |
| gi\|578840995 (+5) | PREDICTED: golgin A8 family, member N isoform X3 [Homo sapiens] | 1 | 0 | 0 | 0 |
| gi\|7657655 (+1) | translocating chain-associated membrane protein 1 [Homo sapiens] | 1 | 0 | 0 | 0 |
| gi\|4505689 | [Pyruvate dehydrogenase (acetyl-transferring)] kinase isozyme 1, mitochondrial isoform 2 precursor [Homo sapiens] | 1 | 0 | 0 | 0 |
| gi\|187281616 | NADH dehydrogenase [ubiquinone] iron-sulfur protein 7, mitochondrial [Homo sapiens] | 1 | 0 | 0 | 0 |
| gi\|557440899 (+15) | nuclear mitotic apparatus protein 1 isoform 2 [Homo sapiens] | 1 | 0 | 0 | 0 |
| gi\|58331268 | DNA excision repair protein ERCC-6-like [Homo sapiens] | 1 | 0 | 0 | 0 |
| gi\|23308722 (+3) | dual specificity protein kinase TTK isoform 1 [Homo sapiens] | 1 | 0 | 0 | 1 |
| gi\|300934762 (+2) | coronin-1A [Homo sapiens] | 1 | 0 | 0 | 0 |
| gi\|284448556 (+5) | 2-amino-3-ketobutyrate coenzyme A ligase, mitochondrial isoform 1 precursor [Homo sapiens] | 1 | 0 | 0 | 0 |
| gi\|22094987 | regulatory-associated protein of mTOR isoform 1 [Homo sapiens] | 1 | 0 | 0 | 1 |
| gi\|767934307 | PREDICTED: T-complex protein 1 subunit epsilon isoform X1 [Homo sapiens] | 1 | 0 | 0 | 1 |
| gi\|767995463 (+1) | PREDICTED: E3 ubiquitin-protein ligase RNF213 isoform X2 [Homo sapiens] | 1 | 0 | 0 | 0 |
| gi\|5453601 | cartilage-associated protein precursor [Homo sapiens] | 1 | 0 | 0 | 0 |
| gi\|578824490 (+2) | PREDICTED: kinetochore-associated protein 1 isoform X1 [Homo sapiens] | 1 | 0 | 0 | 0 |
| gi\|49355721 | protein FAM162A [Homo sapiens] | 1 | 0 | 0 | 0 |
| gi\|4506701 | 40S ribosomal protein S23 [Homo sapiens] | 1 | 0 | 0 | 1 |
| gi\|767947378 | PREDICTED: transducin beta-like protein 2 isoform X2 [Homo sapiens] | 1 | 0 | 0 | 0 |
| gi\|188497683 (+1) | FAST kinase domain-containing protein 1 isoform 1 [Homo sapiens] | 1 | 0 | 0 | 0 |
| gi\|31621303 | sideroflexin-3 [Homo sapiens] | 1 | 0 | 0 | 0 |
| gi\|47419909 (+1) | transcription intermediary factor 1-alpha isoform b [Homo sapiens] | 1 | 0 | 0 | 0 |
| gi\|118572599 (+4) | FH1/FH2 domain-containing protein 1 [Homo sapiens] | 1 | 0 | 0 | 0 |
| gi\|5729810 | 3-beta-hydroxysteroid-Delta(8),Delta(7)-isomerase [Homo sapiens] | 1 | 0 | 0 | 0 |
| gi\|520261712 (+2) | NF-X1-type zinc finger protein NFXL1 [Homo sapiens] | 1 | 0 | 0 | 0 |
| gi\|285002231 (+5) | glycerol-3-phosphate dehydrogenase, mitochondrial precursor [Homo sapiens] | 1 | 0 | 0 | 0 |
| gi\|530402054 (+2) | PREDICTED: heat shock protein 105 kDa isoform X2 [Homo sapiens] | 1 | 0 | 0 | 0 |
| gi\|31377644 (+2) | ATPase family AAA domain-containing protein 1 [Homo sapiens] | 1 | 0 | 0 | 0 |
| gi\|156564401 (+3) | vesicle-fusing ATPase [Homo sapiens] | 1 | 0 | 0 | 0 |
| gi\|17978489 (+3) | CD97 antigen isoform 2 preproprotein [Homo sapiens] | 1 | 0 | 0 | 0 |
| gi\|578832950 (+1) | PREDICTED: transmembrane emp24 domain-containing protein 1 isoform X1 [Homo sapiens] | 1 | 0 | 0 | 0 |
| gi\|30578418 (+1) | sideroflexin-2 [Homo sapiens] | 1 | 0 | 0 | 0 |
| gi\|38492356 (+1) | DNA polymerase delta subunit 3 [Homo sapiens] | 1 | 0 | 0 | 0 |
| gi\|24308211 | integrator complex subunit 2 [Homo sapiens] | 1 | 0 | 0 | 0 |
| gi\|7242140 (+1) | ATP-dependent Clp protease ATP-binding subunit clpX-like, mitochondrial precursor [Homo sapiens] | 1 | 0 | 0 | 0 |
| gi\|260763955 | NADH dehydrogenase [ubiquinone] 1 alpha subcomplex subunit 13 [Homo sapiens] | 1 | 0 | 0 | 0 |
| gi\|269847874 (+2) | probable ATP-dependent RNA helicase YTHDC2 [Homo sapiens] | 1 | 0 | 0 | 0 |
| gi\|125656165 (+10) | THO complex subunit 2 [Homo sapiens] | 1 | 0 | 0 | 0 |
| gi\|9910382 | mitochondrial import receptor subunit TOM22 homolog [Homo sapiens] | 1 | 0 | 0 | 0 |
| gi\|4504041 | guanine nucleotide-binding protein G(i) subunit alpha-2 isoform 1 [Homo sapiens] | 1 | 0 | 0 | 0 |
| gi\|55741709 (+2) | RNA-binding protein 25 [Homo sapiens] | 1 | 0 | 0 | 1 |
| gi\|6274550 | NADH dehydrogenase [ubiquinone] 1 beta subcomplex subunit 9 isoform 1 [Homo sapiens] | 1 | 0 | 0 | 0 |
| gi\|8922794 | U3 small nucleolar ribonucleoprotein protein IMP3 [Homo sapiens] | 1 | 0 | 0 | 0 |
| gi\|47132589 (+3) | serine/threonine-protein kinase N1 isoform 2 [Homo sapiens] | 1 | 0 | 0 | 1 |
| gi\|224177528 | small integral membrane protein 20 [Homo sapiens] | 1 | 0 | 0 | 0 |
| gi\|8922886 | ATP-dependent RNA helicase DDX19A [Homo sapiens] | 1 | 0 | 0 | 0 |
| gi\|28178838 (+1) | isocitrate dehydrogenase [NAD] subunit gamma, mitochondrial isoform b precursor [Homo sapiens] | 1 | 0 | 0 | 0 |
| gi\|12056468 (+11) | junction plakoglobin [Homo sapiens] | 1 | 0 | 0 | 0 |
| gi\|171906589 (+2) | [3-methyl-2-oxobutanoate dehydrogenase [lipoamide]] kinase, mitochondrial isoform a precursor [Homo sapiens] | 1 | 0 | 0 | 0 |
| gi\|767941789 (+3) | PREDICTED: activating signal cointegrator 1 complex subunit 3 isoform X1 [Homo sapiens] | 1 | 0 | 0 | 0 |
| gi\|55956766 (+2) | etoposide-induced protein 2.4 homolog isoform 1 [Homo sapiens] | 1 | 0 | 0 | 0 |
| gi\|544063439 | UDP-galactose translocator isoform h [Homo sapiens] | 1 | 0 | 0 | 0 |
| gi\|4503253 | dolichyl-diphosphooligosaccharide--protein glycosyltransferase subunit DAD1 [Homo sapiens] | 1 | 0 | 0 | 0 |
| gi\|6041665 | 1-acyl-sn-glycerol-3-phosphate acyltransferase beta isoform a precursor [Homo sapiens] | 1 | 0 | 0 | 0 |
| gi\|221136939 (+10) | U4/U6 small nuclear ribonucleoprotein Prp31 [Homo sapiens] | 1 | 0 | 0 | 0 |
| gi\|4758988 (+1) | ras-related protein Rab-1A isoform 1 [Homo sapiens] | 1 | 0 | 0 | 0 |
| gi\|767942397 (+5) | PREDICTED: PHD finger protein 3 isoform X3 [Homo sapiens] | 1 | 0 | 0 | 0 |
| gi\|4507831 (+2) | serine/threonine-protein kinase ULK1 [Homo sapiens] | 1 | 0 | 0 | 0 |
| gi\|530361606 | PREDICTED: leucine-rich repeat-containing protein 41 isoform X1 [Homo sapiens] | 1 | 0 | 0 | 1 |
| gi\|18765700 (+5) | engulfment and cell motility protein 1 isoform 1 [Homo sapiens] | 1 | 0 | 0 | 0 |
| gi\|190014578 (+2) | phosphatidylinositide phosphatase SAC1 [Homo sapiens] | 1 | 0 | 0 | 0 |
| gi\|33300633 | integrator complex subunit 11 isoform 2 [Homo sapiens] | 1 | 0 | 0 | 0 |
| gi\|13376162 | protein FAM57A [Homo sapiens] | 1 | 0 | 0 | 0 |
| gi\|767990533 | PREDICTED: transport and Golgi organization protein 6 homolog isoform X1 [Homo sapiens] | 1 | 0 | 0 | 0 |
| gi\|45505139 | phosphoinositide 3-kinase adapter protein 1 [Homo sapiens] | 1 | 0 | 0 | 0 |
| gi\|21686999 | metalloendopeptidase OMA1, mitochondrial precursor [Homo sapiens] | 1 | 0 | 0 | 0 |
| gi\|23397653 (+2) | GPI transamidase component PIG-T isoform 1 precursor [Homo sapiens] | 1 | 0 | 0 | 0 |
| gi\|254939537 (+12) | unconventional myosin-XIX isoform 2 [Homo sapiens] | 1 | 0 | 0 | 0 |
| gi\|767993476 (+1) | PREDICTED: putative sodium-coupled neutral amino acid transporter 10 isoform X3 [Homo sapiens] | 1 | 0 | 0 | 0 |
| gi\|5031789 | inositol monophosphatase 1 isoform 1 [Homo sapiens] | 1 | 0 | 0 | 0 |
| gi\|157388904 | dynein assembly factor 5, axonemal [Homo sapiens] | 1 | 0 | 0 | 0 |
| gi\|316983124 (+1) | 60S ribosomal protein L36a isoform a [Homo sapiens] | 1 | 0 | 0 | 0 |
| gi\|18644728 | nucleolar protein 6 alpha isoform [Homo sapiens] | 1 | 0 | 0 | 0 |
| gi\|56549145 (+5) | metalloreductase STEAP3 isoform a [Homo sapiens] | 1 | 0 | 0 | 0 |
| gi\|4757988 | probable cytosolic iron-sulfur protein assembly protein CIAO1 [Homo sapiens] | 1 | 0 | 0 | 0 |
| gi\|15011880 | serine/threonine-protein kinase 26 isoform 1 [Homo sapiens] | 1 | 0 | 0 | 0 |
| gi\|372266129 (+2) | protein flightless-1 homolog isoform 3 [Homo sapiens] | 1 | 0 | 0 | 0 |
| gi\|48762932 (+2) | T-complex protein 1 subunit theta isoform 1 [Homo sapiens] | 1 | 0 | 0 | 0 |
| gi\|31077094 (+4) | ceramide synthase 2 [Homo sapiens] | 1 | 0 | 0 | 0 |
| gi\|14149740 (+3) | oxysterol-binding protein-related protein 5 isoform a [Homo sapiens] | 1 | 0 | 0 | 2 |
| gi\|566006200 | G1/S-specific cyclin-D3 isoform 5 [Homo sapiens] | 1 | 0 | 0 | 0 |
| gi\|38683855 | pentatricopeptide repeat domain-containing protein 3, mitochondrial precursor [Homo sapiens] | 1 | 0 | 0 | 0 |
| gi\|578830685 (+7) | PREDICTED: protein TANC2 isoform X3 [Homo sapiens] | 1 | 0 | 0 | 0 |
| gi\|56121818 (+1) | probable dolichyl pyrophosphate Glc1Man9GlcNAc2 alpha-1,3-glucosyltransferase isoform a [Homo sapiens] | 1 | 0 | 0 | 0 |
| gi\|530400597 (+1) | PREDICTED: nuclear pore complex protein Nup107 isoform X1 [Homo sapiens] | 1 | 0 | 0 | 0 |
| gi\|296317239 (+1) | peroxisomal membrane protein 11B isoform 2 [Homo sapiens] | 1 | 0 | 0 | 0 |
| gi\|49574529 | U3 small nucleolar RNA-associated protein 6 homolog [Homo sapiens] | 1 | 0 | 0 | 0 |
| gi\|153791300 (+1) | protein SDA1 homolog isoform 1 [Homo sapiens] | 1 | 0 | 0 | 0 |
| gi\|4758032 | coatomer subunit beta' [Homo sapiens] | 1 | 0 | 0 | 0 |
| gi\|6912398 | general transcription factor 3C polypeptide 3 isoform 1 [Homo sapiens] | 1 | 0 | 0 | 0 |
| gi\|4557403 | mitochondrial carnitine/acylcarnitine carrier protein [Homo sapiens] | 1 | 0 | 0 | 0 |
| gi\|13124891 (+1) | polypeptide N-acetylgalactosaminyltransferase 1 [Homo sapiens] | 1 | 0 | 0 | 0 |
| gi\|635372911 | TNFAIP3-interacting protein 2 isoform 3 [Homo sapiens] | 1 | 0 | 0 | 0 |
| gi\|5729802 | thioredoxin-like protein 4A isoform 1 [Homo sapiens] | 1 | 0 | 0 | 0 |
| gi\|530361577 (+2) | PREDICTED: peptidyl-prolyl cis-trans isomerase H isoform X1 [Homo sapiens] | 1 | 0 | 0 | 0 |
| gi\|94721250 (+1) | vesicle-associated membrane protein-associated protein A isoform 1 [Homo sapiens] | 1 | 0 | 0 | 0 |
| gi\|20127553 (+2) | anaphase-promoting complex subunit 5 isoform a [Homo sapiens] | 1 | 0 | 0 | 0 |
| gi\|239788847 (+2) | jmjC domain-containing protein 4 isoform 1 [Homo sapiens] | 1 | 0 | 0 | 0 |
| gi\|5729770 | tripeptidyl-peptidase 1 preproprotein [Homo sapiens] | 1 | 0 | 0 | 0 |
| gi\|57222565 | serine/threonine-protein phosphatase 2A catalytic subunit beta isoform [Homo sapiens] | 1 | 0 | 0 | 0 |
| gi\|227498241 (+8) | actin-like protein 8 [Homo sapiens] | 1 | 0 | 0 | 0 |
| gi\|578825386 | PREDICTED: cell division cycle protein 16 homolog isoform X5 [Homo sapiens] | 1 | 0 | 0 | 0 |
| gi\|10880989 (+3) | ras-related protein Rab-18 isoform 1 [Homo sapiens] | 1 | 0 | 0 | 0 |
| gi\|11125768 (+1) | eukaryotic translation initiation factor 2-alpha kinase 1 isoform a [Homo sapiens] | 1 | 0 | 0 | 0 |
| gi\|38045913 (+1) | nucleoside diphosphate kinase A isoform a [Homo sapiens] | 1 | 0 | 0 | 0 |
| gi\|4506773 | protein S100-A9 [Homo sapiens] | 1 | 0 | 0 | 0 |
| gi\|301172750 | mucin-5B precursor [Homo sapiens] | 1 | 0 | 0 | 0 |
| gi\|530424054 (+3) | PREDICTED: E3 ubiquitin-protein ligase RFWD3 isoform X1 [Homo sapiens] | 1 | 0 | 0 | 0 |
| gi\|530423538 (+1) | PREDICTED: transcriptional repressor CTCF isoform X1 [Homo sapiens] | 1 | 0 | 0 | 0 |
| gi\|23308579 (+8) | prostaglandin E synthase 3 isoform a [Homo sapiens] | 1 | 0 | 0 | 0 |
| gi\|21361437 (+2) | HIV Tat-specific factor 1 [Homo sapiens] | 1 | 0 | 0 | 0 |
| gi\|119943100 (+1) | propionyl-CoA carboxylase beta chain, mitochondrial isoform 1 precursor [Homo sapiens] | 1 | 0 | 0 | 0 |
| gi\|767881831 (+3) | PREDICTED: zinc finger protein Rlf-like isoform X1 [Homo sapiens] | 1 | 0 | 0 | 1 |
| gi\|4826649 | 39S ribosomal protein L49, mitochondrial [Homo sapiens] | 1 | 0 | 0 | 0 |
| gi\|257153459 (+5) | disrupted in schizophrenia 1 protein isoform a [Homo sapiens] | 1 | 0 | 0 | 0 |
| gi\|116089325 (+6) | splicing regulatory glutamine/lysine-rich protein 1 isoform a [Homo sapiens] | 1 | 0 | 0 | 0 |
| gi\|251831116 | NADH dehydrogenase subunit 4 (mitochondrion) [Homo sapiens] | 1 | 0 | 0 | 0 |
| gi\|15011936 (+1) | 40S ribosomal protein S26 [Homo sapiens] | 1 | 1 | 3 | 1 |
| gi\|347446678 | putative RNA-binding protein Luc7-like 2 isoform 3 [Homo sapiens] | 1 | 1 | 2 | 3 |
| gi\|54112117 | splicing factor 3B subunit 1 isoform 1 [Homo sapiens] | 1 | 1 | 2 | 2 |
| gi\|283135173 (+3) | RNA-binding protein FUS isoform 3 [Homo sapiens] | 1 | 1 | 2 | 2 |
| gi\|10947135 (+1) | ATP-binding cassette sub-family F member 1 isoform b [Homo sapiens] | 1 | 1 | 2 | 1 |
| gi\|56243583 | WD repeat-containing protein 18 [Homo sapiens] | 1 | 1 | 2 | 3 |
| gi\|20070125 | protein disulfide-isomerase precursor [Homo sapiens] | 1 | 1 | 1 | 2 |
| gi\|530370697 (+1) | PREDICTED: pre-mRNA-splicing factor CWC22 homolog isoform X1 [Homo sapiens] | 1 | 1 | 1 | 5 |
| gi\|71979932 | large neutral amino acids transporter small subunit 1 [Homo sapiens] | 1 | 1 | 1 | 1 |
| gi\|7657015 | tRNA-splicing ligase RtcB homolog [Homo sapiens] | 1 | 1 | 1 | 2 |
| gi\|5032051 (+2) | 40S ribosomal protein S14 [Homo sapiens] | 1 | 1 | 1 | 3 |
| gi\|5803165 | protein transport protein Sec61 subunit beta [Homo sapiens] | 1 | 1 | 1 | 1 |
| gi\|767952602 | PREDICTED: 2,4-dienoyl-CoA reductase, mitochondrial isoform X1 [Homo sapiens] | 1 | 1 | 0 | 0 |
| gi\|392306969 (+5) | activating molecule in BECN1-regulated autophagy protein 1 isoform 1 [Homo sapiens] | 1 | 1 | 0 | 2 |
| gi\|222352151 | poly(rC)-binding protein 1 [Homo sapiens] | 1 | 1 | 0 | 0 |
| gi\|349732256 (+1) | thioredoxin isoform 2 [Homo sapiens] | 1 | 1 | 0 | 1 |
| gi\|21314632 | neutral amino acid transporter A isoform 1 [Homo sapiens] | 1 | 1 | 0 | 0 |
| gi\|4504261 | histone H2B type 1-N [Homo sapiens] | 1 | 1 | 0 | 1 |
| gi\|21361368 (+2) | delta-1-pyrroline-5-carboxylate synthase isoform 1 [Homo sapiens] | 1 | 1 | 0 | 0 |
| gi\|108796056 (+8) | 60 kDa SS-A/Ro ribonucleoprotein isoform 1 [Homo sapiens] | 1 | 1 | 0 | 0 |
| gi\|767925767 | PREDICTED: tetratricopeptide repeat protein 14 isoform X1 [Homo sapiens] | 1 | 1 | 0 | 0 |
| gi\|74136883 | heterogeneous nuclear ribonucleoprotein U isoform a [Homo sapiens] | 1 | 2 | 10 | 6 |
| gi\|5803225 | 14-3-3 protein epsilon [Homo sapiens] | 1 | 2 | 2 | 1 |
| gi\|5730009 | zinc finger protein RFP [Homo sapiens] | 1 | 2 | 2 | 4 |
| gi\|10835063 | nucleophosmin isoform 1 [Homo sapiens] | 1 | 2 | 1 | 2 |
| gi\|767963460 (+4) | PREDICTED: 40S ribosomal protein S24 isoform X1 [Homo sapiens] | 1 | 2 | 1 | 1 |
| gi\|4506707 | 40S ribosomal protein S25 [Homo sapiens] | 1 | 2 | 1 | 2 |
| gi\|530427501 (+1) | PREDICTED: heterogeneous nuclear ribonucleoprotein M isoform X1 [Homo sapiens] | 1 | 2 | 0 | 1 |
| gi\|6912634 | 60S ribosomal protein L13a isoform 1 [Homo sapiens] | 1 | 2 | 0 | 0 |
| gi\|15431306 (+2) | 60S ribosomal protein L8 [Homo sapiens] | 1 | 2 | 0 | 1 |
| gi\|4506623 | 60S ribosomal protein L27 [Homo sapiens] | 1 | 2 | 0 | 0 |
| gi\|130502162 (+1) | RAS protein activator like-3 [Homo sapiens] | 1 | 3 | 7 | 8 |
| gi\|24308295 | grpE protein homolog 1, mitochondrial precursor [Homo sapiens] | 1 | 3 | 4 | 0 |
| gi\|15055539 | 40S ribosomal protein S2 [Homo sapiens] | 1 | 3 | 2 | 3 |
| gi\|78000181 (+1) | 60S ribosomal protein L14 [Homo sapiens] | 1 | 3 | 1 | 2 |
| gi\|15431290 | 60S ribosomal protein L11 isoform 1 [Homo sapiens] | 1 | 3 | 1 | 2 |
| gi\|94721239 (+1) | isoleucine--tRNA ligase, cytoplasmic [Homo sapiens] | 1 | 3 | 0 | 0 |
| gi\|100913206 | ATP-dependent RNA helicase A [Homo sapiens] | 1 | 4 | 2 | 1 |
| gi\|4503305 | defensin-6 preproprotein [Homo sapiens] | 1 | 4 | 2 | 0 |
| gi\|221307584 | prohibitin-2 isoform 1 [Homo sapiens] | 1 | 4 | 0 | 1 |
| gi\|4506743 | 40S ribosomal protein S8 [Homo sapiens] | 1 | 5 | 0 | 3 |
| gi\|11321640 | beta-1-syntrophin [Homo sapiens] | 1 | 5 | 0 | 0 |
| gi\|15431288 | 60S ribosomal protein L10a [Homo sapiens] | 1 | 5 | 0 | 0 |
| gi\|89179321 | protein unc-45 homolog A isoform 3 [Homo sapiens] | 1 | 6 | 0 | 0 |
| gi\|15149476 | arginine--tRNA ligase, cytoplasmic [Homo sapiens] | 1 | 7 | 0 | 0 |
| gi\|38679967 (+4) | acetyl-CoA carboxylase 1 isoform 2 [Homo sapiens] | 1 | 11 | 0 | 0 |
| gi\|57863271 | von Willebrand factor A domain-containing protein 8 isoform a precursor [Homo sapiens] | 1 | 24 | 0 | 0 |
| gi\|126032348 (+2) | E3 ubiquitin-protein ligase HERC2 [Homo sapiens] | 2 | 0 | 20 | 39 |
| gi\|21361794 | cullin-associated NEDD8-dissociated protein 1 [Homo sapiens] | 2 | 0 | 6 | 5 |
| gi\|73760405 | thymopoietin isoform beta [Homo sapiens] | 2 | 0 | 3 | 2 |
| gi\|209976986 (+5) | PCI domain-containing protein 2 isoform 1 [Homo sapiens] | 2 | 0 | 3 | 1 |
| gi\|6681764 | NADH dehydrogenase [ubiquinone] 1 alpha subcomplex subunit 9, mitochondrial precursor [Homo sapiens] | 2 | 0 | 2 | 1 |
| gi\|65506891 | 4F2 cell-surface antigen heavy chain isoform c [Homo sapiens] | 2 | 0 | 2 | 0 |
| gi\|167466198 | intercellular adhesion molecule 1 precursor [Homo sapiens] | 2 | 0 | 2 | 1 |
| gi\|22202629 (+1) | apoptosis-inducing factor 1, mitochondrial isoform 2 precursor [Homo sapiens] | 2 | 0 | 2 | 1 |
| gi\|371872752 (+2) | transmembrane and coiled-coil domain-containing protein 1 isoform a [Homo sapiens] | 2 | 0 | 2 | 0 |
| gi\|10716563 (+3) | calnexin precursor [Homo sapiens] | 2 | 0 | 2 | 0 |
| gi\|224589127 (+2) | transmembrane protein 33 [Homo sapiens] | 2 | 0 | 1 | 1 |
| gi\|148224884 | phosphatidylglycerophosphatase and protein-tyrosine phosphatase 1 isoform 1 [Homo sapiens] | 2 | 0 | 1 | 0 |
| gi\|4503607 | electron transfer flavoprotein subunit alpha, mitochondrial isoform a [Homo sapiens] | 2 | 0 | 1 | 0 |
| gi\|24308201 | adipocyte plasma membrane-associated protein [Homo sapiens] | 2 | 0 | 1 | 0 |
| gi\|223718097 | mitochondrial inner membrane protein OXA1L [Homo sapiens] | 2 | 0 | 1 | 0 |
| gi\|38505222 | protein disulfide-isomerase TMX3 precursor [Homo sapiens] | 2 | 0 | 1 | 1 |
| gi\|767905372 | PREDICTED: ATP-binding cassette sub-family D member 3 isoform X1 [Homo sapiens] | 2 | 0 | 1 | 0 |
| gi\|4506381 | ras-related C3 botulinum toxin substrate 2 [Homo sapiens] | 2 | 0 | 1 | 0 |
| gi\|7019569 | vacuolar protein sorting-associated protein 4A [Homo sapiens] | 2 | 0 | 1 | 0 |
| gi\|56676371 (+1) | cleavage and polyadenylation specificity factor subunit 1 [Homo sapiens] | 2 | 0 | 1 | 3 |
| gi\|18699732 (+1) | 45 kDa calcium-binding protein isoform 2 precursor [Homo sapiens] | 2 | 0 | 1 | 1 |
| gi\|14042968 (+3) | protein spinster homolog 1 isoform 1 [Homo sapiens] | 2 | 0 | 1 | 2 |
| gi\|5032027 | histone-binding protein RBBP4 isoform a [Homo sapiens] | 2 | 0 | 1 | 1 |
| gi\|4502297 (+1) | ATP synthase subunit delta, mitochondrial precursor [Homo sapiens] | 2 | 0 | 1 | 0 |
| gi\|8922720 | cell cycle control protein 50A isoform 1 [Homo sapiens] | 2 | 0 | 1 | 0 |
| gi\|767951299 | PREDICTED: disintegrin and metalloproteinase domain-containing protein 9 isoform X1 [Homo sapiens] | 2 | 0 | 1 | 0 |
| gi\|45593130 (+2) | guanine nucleotide-binding protein-like 3 isoform 1 [Homo sapiens] | 2 | 0 | 0 | 1 |
| gi\|20070197 | dolichyl-diphosphooligosaccharide--protein glycosyltransferase 48 kDa subunit precursor [Homo sapiens] | 2 | 0 | 0 | 0 |
| gi\|4503571 | alpha-enolase isoform 1 [Homo sapiens] | 2 | 0 | 0 | 0 |
| gi\|22749415 (+2) | dolichyl-diphosphooligosaccharide--protein glycosyltransferase subunit STT3A isoform a [Homo sapiens] | 2 | 0 | 0 | 0 |
| gi\|5803133 | ras-related protein Rab-32 [Homo sapiens] | 2 | 0 | 0 | 0 |
| gi\|10947137 (+3) | ATP-binding cassette sub-family F member 2 isoform b [Homo sapiens] | 2 | 0 | 0 | 0 |
| gi\|4507943 (+3) | exportin-1 [Homo sapiens] | 2 | 0 | 0 | 0 |
| gi\|11641289 | dimethyladenosine transferase 2, mitochondrial [Homo sapiens] | 2 | 0 | 0 | 1 |
| gi\|6041669 | NADH dehydrogenase [ubiquinone] 1 beta subcomplex subunit 4 isoform 1 [Homo sapiens] | 2 | 0 | 0 | 1 |
| gi\|4504191 (+5) | DNA mismatch repair protein Msh6 isoform 1 [Homo sapiens] | 2 | 0 | 0 | 0 |
| gi\|58530840 | desmoplakin isoform I [Homo sapiens] | 2 | 0 | 0 | 0 |
| gi\|262399371 | transmembrane protein 201 isoform 1 [Homo sapiens] | 2 | 0 | 0 | 2 |
| gi\|300863094 (+1) | fMet-Leu-Phe receptor [Homo sapiens] | 2 | 0 | 0 | 2 |
| gi\|767979244 | PREDICTED: lysosome-associated membrane glycoprotein 1 isoform X1 [Homo sapiens] | 2 | 0 | 0 | 0 |
| gi\|119120894 (+3) | dmX-like protein 2 isoform 2 [Homo sapiens] | 2 | 0 | 0 | 0 |
| gi\|37620210 | cytochrome c oxidase protein 20 homolog [Homo sapiens] | 2 | 0 | 0 | 1 |
| gi\|4557553 | emerin [Homo sapiens] | 2 | 0 | 0 | 1 |
| gi\|134142062 (+8) | acetyl-CoA carboxylase 2 precursor [Homo sapiens] | 2 | 0 | 0 | 0 |
| gi\|4507835 | uridine 5'-monophosphate synthase [Homo sapiens] | 2 | 0 | 0 | 0 |
| gi\|145864459 | diacylglycerol O-acyltransferase 1 [Homo sapiens] | 2 | 0 | 0 | 0 |
| gi\|21389315 (+1) | tricarboxylate transport protein, mitochondrial isoform a precursor [Homo sapiens] | 2 | 0 | 0 | 0 |
| gi\|116642885 | probable hydrolase PNKD isoform 3 precursor [Homo sapiens] | 2 | 0 | 0 | 1 |
| gi\|190194386 (+1) | transmembrane 9 superfamily member 3 precursor [Homo sapiens] | 2 | 0 | 0 | 0 |
| gi\|530388425 | PREDICTED: DNA replication licensing factor MCM4 isoform X1 [Homo sapiens] | 2 | 0 | 0 | 2 |
| gi\|4759226 (+3) | TGF-beta receptor type-1 isoform 1 precursor [Homo sapiens] | 2 | 0 | 0 | 0 |
| gi\|47717100 (+3) | V-type proton ATPase subunit H isoform 2 [Homo sapiens] | 2 | 0 | 0 | 0 |
| gi\|61097912 | nucleolar MIF4G domain-containing protein 1 [Homo sapiens] | 2 | 0 | 0 | 1 |
| gi\|7661948 | malectin isoform1 precursor [Homo sapiens] | 2 | 0 | 0 | 0 |
| gi\|117190519 | sialidase-3 [Homo sapiens] | 2 | 0 | 0 | 0 |
| gi\|110624774 | C-type mannose receptor 2 precursor [Homo sapiens] | 2 | 0 | 0 | 0 |
| gi\|51242945 (+2) | striatin [Homo sapiens] | 2 | 0 | 0 | 0 |
| gi\|164607128 | solute carrier family 35 member E1 [Homo sapiens] | 2 | 0 | 0 | 1 |
| gi\|530387374 (+6) | PREDICTED: kelch repeat and BTB domain-containing protein 11 isoform X1 [Homo sapiens] | 2 | 0 | 0 | 1 |
| gi\|13376259 (+1) | nuclear pore complex protein Nup85 isoform 1 [Homo sapiens] | 2 | 0 | 0 | 0 |
| gi\|735367775 (+1) | integrin beta-2 isoform 1 precursor [Homo sapiens] | 2 | 0 | 0 | 0 |
| gi\|10880134 (+5) | beta-arrestin-1 isoform B [Homo sapiens] | 2 | 0 | 0 | 0 |
| gi\|110347418 (+10) | structural maintenance of chromosomes protein 2 [Homo sapiens] | 2 | 0 | 0 | 0 |
| gi\|21361565 | ATP synthase F(0) complex subunit B1, mitochondrial precursor [Homo sapiens] | 2 | 0 | 0 | 0 |
| gi\|767918774 | PREDICTED: sphingomyelin phosphodiesterase 4 isoform X2 [Homo sapiens] | 2 | 0 | 0 | 0 |
| gi\|6912482 | LETM1 and EF-hand domain-containing protein 1, mitochondrial precursor [Homo sapiens] | 2 | 0 | 0 | 0 |
| gi\|578825502 | PREDICTED: mitochondrial basic amino acids transporter isoform X4 [Homo sapiens] | 2 | 0 | 0 | 0 |
| gi\|544346307 (+1) | peroxisomal membrane protein PMP34 isoform 2 [Homo sapiens] | 2 | 0 | 0 | 0 |
| gi\|5453998 | importin-7 [Homo sapiens] | 2 | 0 | 0 | 0 |
| gi\|530362357 (+3) | PREDICTED: tyrosine-protein phosphatase non-receptor type 22 isoform X1 [Homo sapiens] | 2 | 0 | 0 | 0 |
| gi\|12669909 (+7) | long-chain-fatty-acid--CoA ligase 4 isoform 2 [Homo sapiens] | 2 | 0 | 0 | 0 |
| gi\|188035908 (+2) | chromobox protein homolog 5 [Homo sapiens] | 2 | 0 | 0 | 0 |
| gi\|19718764 (+5) | ubiquitin-protein ligase E3A isoform 3 [Homo sapiens] | 2 | 0 | 0 | 0 |
| gi\|44771201 | integrator complex subunit 5 [Homo sapiens] | 2 | 0 | 0 | 0 |
| gi\|120952851 (+2) | WASH complex subunit strumpellin [Homo sapiens] | 2 | 0 | 0 | 0 |
| gi\|4506019 (+1) | serine/threonine-protein phosphatase 2A 55 kDa regulatory subunit B alpha isoform isoform 1 [Homo sapiens] | 2 | 0 | 0 | 0 |
| gi\|255652953 (+3) | zinc finger CCCH domain-containing protein 18 isoform 2 [Homo sapiens] | 2 | 0 | 0 | 0 |
| gi\|4758768 | NADH dehydrogenase [ubiquinone] 1 alpha subcomplex subunit 10, mitochondrial precursor [Homo sapiens] | 2 | 0 | 0 | 0 |
| gi\|10190704 | peroxisomal 2,4-dienoyl-CoA reductase [Homo sapiens] | 2 | 0 | 0 | 0 |
| gi\|24497447 (+7) | nuclear pore complex protein Nup50 isoform a [Homo sapiens] | 2 | 0 | 0 | 0 |
| gi\|13236587 | transmembrane protein 43 [Homo sapiens] | 2 | 0 | 0 | 0 |
| gi\|194018488 (+1) | peroxisome biogenesis factor 6 [Homo sapiens] | 2 | 0 | 0 | 1 |
| gi\|6679189 | transmembrane emp24 domain-containing protein 3 isoform a precursor [Homo sapiens] | 2 | 0 | 0 | 0 |
| gi\|4506217 | 26S proteasome non-ATPase regulatory subunit 10 isoform 1 [Homo sapiens] | 2 | 0 | 0 | 0 |
| gi\|21361399 | serine/threonine-protein phosphatase 2A 65 kDa regulatory subunit A alpha isoform [Homo sapiens] | 2 | 0 | 0 | 0 |
| gi\|98986331 | pentatricopeptide repeat-containing protein 2, mitochondrial isoform 1 [Homo sapiens] | 2 | 0 | 0 | 0 |
| gi\|304555614 (+2) | BRCA1-associated ATM activator 1 [Homo sapiens] | 2 | 0 | 0 | 0 |
| gi\|21359965 (+1) | cleft lip and palate transmembrane protein 1-like protein [Homo sapiens] | 2 | 0 | 0 | 0 |
| gi\|46195765 (+3) | protein unc-13 homolog D [Homo sapiens] | 2 | 0 | 0 | 4 |
| gi\|767940022 (+7) | PREDICTED: ras-related protein Rab-44 isoform X6 [Homo sapiens] | 2 | 0 | 0 | 0 |
| gi\|8923380 | probable tRNA N6-adenosine threonylcarbamoyltransferase [Homo sapiens] | 2 | 0 | 0 | 0 |
| gi\|530381698 (+2) | PREDICTED: E3 ubiquitin-protein ligase UBR2 isoform X1 [Homo sapiens] | 2 | 0 | 0 | 0 |
| gi\|12056971 (+1) | anaphase-promoting complex subunit 1 [Homo sapiens] | 2 | 0 | 0 | 0 |
| gi\|110825988 (+2) | probable methyltransferase TARBP1 [Homo sapiens] | 2 | 0 | 0 | 0 |
| gi\|42542394 | lysophospholipid acyltransferase 5 [Homo sapiens] | 2 | 0 | 0 | 0 |
| gi\|767998859 (+3) | PREDICTED: dymeclin isoform X1 [Homo sapiens] | 2 | 0 | 0 | 0 |
| gi\|118402596 | cell division cycle protein 23 homolog [Homo sapiens] | 2 | 0 | 0 | 0 |
| gi\|266453278 (+1) | ataxin-10 isoform 2 [Homo sapiens] | 2 | 0 | 0 | 0 |
| gi\|212276104 (+3) | leucine-rich repeat flightless-interacting protein 1 isoform 5 [Homo sapiens] | 2 | 0 | 0 | 1 |
| gi\|55741641 | kinase D-interacting substrate of 220 kDa [Homo sapiens] | 2 | 0 | 0 | 0 |
| gi\|387849014 (+7) | disintegrin and metalloproteinase domain-containing protein 15 isoform 7 preproprotein [Homo sapiens] | 2 | 0 | 0 | 0 |
| gi\|578822203 (+8) | PREDICTED: serine-protein kinase ATM isoform X1 [Homo sapiens] | 2 | 0 | 0 | 0 |
| gi\|27544941 (+3) | unconventional myosin-If [Homo sapiens] | 2 | 0 | 0 | 0 |
| gi\|578811862 (+8) | PREDICTED: helicase SKI2W isoform X1 [Homo sapiens] | 2 | 0 | 0 | 0 |
| gi\|4504505 | peroxisomal multifunctional enzyme type 2 isoform 2 [Homo sapiens] | 2 | 0 | 0 | 0 |
| gi\|22027642 (+4) | kelch-like ECH-associated protein 1 [Homo sapiens] | 2 | 0 | 0 | 0 |
| gi\|19913369 | transducin beta-like protein 3 [Homo sapiens] | 2 | 0 | 0 | 0 |
| gi\|767998516 | PREDICTED: rotatin isoform X6 [Homo sapiens] | 2 | 0 | 0 | 0 |
| gi\|576583519 (+2) | glyceraldehyde-3-phosphate dehydrogenase isoform 1 [Homo sapiens] | 2 | 0 | 0 | 0 |
| gi\|29540531 (+3) | sulfotransferase 1A1 isoform a [Homo sapiens] | 2 | 0 | 0 | 0 |
| gi\|42794756 (+2) | long-chain-fatty-acid--CoA ligase 5 isoform a [Homo sapiens] | 2 | 0 | 0 | 1 |
| gi\|379056374 (+11) | KN motif and ankyrin repeat domain-containing protein 1 isoform a [Homo sapiens] | 2 | 0 | 0 | 0 |
| gi\|224831257 | importin-13 [Homo sapiens] | 2 | 0 | 0 | 0 |
| gi\|205277386 (+1) | glutamine--fructose-6-phosphate aminotransferase [isomerizing] 1 isoform 2 [Homo sapiens] | 2 | 0 | 0 | 0 |
| gi\|21361870 (+1) | A-kinase-interacting protein 1 isoform a [Homo sapiens] | 2 | 0 | 0 | 0 |
| gi\|114326552 (+5) | protein MON2 homolog isoform 1 [Homo sapiens] | 2 | 0 | 0 | 0 |
| gi\|157743267 | ankyrin repeat domain-containing protein SOWAHD [Homo sapiens] | 2 | 0 | 0 | 0 |
| gi\|157389005 | calpain-2 catalytic subunit isoform 1 [Homo sapiens] | 2 | 0 | 0 | 0 |
| gi\|7661788 | mediator of RNA polymerase II transcription subunit 4 isoform 1 [Homo sapiens] | 2 | 0 | 0 | 0 |
| gi\|597955330 (+1) | metaxin-1 isoform 1 [Homo sapiens] | 2 | 0 | 0 | 0 |
| gi\|41327715 | TP53-regulating kinase [Homo sapiens] | 2 | 0 | 0 | 0 |
| gi\|13540555 (+1) | phosphatidylserine synthase 2 [Homo sapiens] | 2 | 0 | 0 | 0 |
| gi\|5729812 | RNA polymerase II elongation factor ELL [Homo sapiens] | 2 | 0 | 0 | 0 |
| gi\|157671949 | zinc finger protein Rlf [Homo sapiens] | 2 | 0 | 0 | 0 |
| gi\|9910346 | ER membrane protein complex subunit 7 precursor [Homo sapiens] | 2 | 0 | 0 | 0 |
| gi\|12597631 (+1) | uncharacterized protein C12orf43 isoform c [Homo sapiens] | 2 | 0 | 0 | 0 |
| gi\|16757970 (+1) | protein Niban [Homo sapiens] | 2 | 0 | 0 | 0 |
| gi\|21361380 | vacuolar-sorting protein SNF8 [Homo sapiens] | 2 | 0 | 0 | 0 |
| gi\|260064013 | ribonucleoside-diphosphate reductase subunit M2 isoform 1 [Homo sapiens] | 2 | 0 | 0 | 0 |
| gi\|45827771 | enhancer of mRNA-decapping protein 4 [Homo sapiens] | 2 | 0 | 0 | 1 |
| gi\|31543630 | sodium-dependent phosphate transporter 1 [Homo sapiens] | 2 | 0 | 0 | 0 |
| gi\|21361851 (+5) | integrator complex subunit 12 [Homo sapiens] | 2 | 0 | 0 | 0 |
| gi\|291575128 (+2) | L-lactate dehydrogenase B chain [Homo sapiens] | 2 | 0 | 0 | 0 |
| gi\|148222745 (+6) | ribonuclease P protein subunit p38 [Homo sapiens] | 2 | 0 | 0 | 0 |
| gi\|768021358 (+1) | PREDICTED: PR domain zinc finger protein 15 isoform X8 [Homo sapiens] | 2 | 0 | 0 | 0 |
| gi\|148277065 (+6) | thioredoxin reductase 1, cytoplasmic isoform 1 [Homo sapiens] | 2 | 0 | 0 | 0 |
| gi\|40807485 | pre-mRNA-processing factor 6 [Homo sapiens] | 2 | 1 | 5 | 15 |
| gi\|17388799 | dnaJ homolog subfamily B member 6 isoform a [Homo sapiens] | 2 | 1 | 3 | 3 |
| gi\|767939343 | PREDICTED: leukocyte elastase inhibitor isoform X2 [Homo sapiens] | 2 | 1 | 2 | 2 |
| gi\|4506635 (+2) | 60S ribosomal protein L32 [Homo sapiens] | 2 | 1 | 0 | 0 |
| gi\|262205273 (+3) | translation initiation factor eIF-2B subunit gamma isoform 2 [Homo sapiens] | 2 | 1 | 0 | 0 |
| gi\|70166599 (+2) | tRNA pseudouridine synthase A, mitochondrial isoform 2 [Homo sapiens] | 2 | 1 | 0 | 0 |
| gi\|13654270 (+1) | ribosomal biogenesis protein LAS1L isoform 1 [Homo sapiens] | 2 | 2 | 4 | 11 |
| gi\|767983063 (+2) | PREDICTED: cytoplasmic FMR1-interacting protein 1 isoform X1 [Homo sapiens] | 2 | 2 | 4 | 4 |
| gi\|13904870 | 40S ribosomal protein S5 [Homo sapiens] | 2 | 2 | 1 | 2 |
| gi\|117168248 | very-long-chain (3R)-3-hydroxyacyl-CoA dehydratase 3 [Homo sapiens] | 2 | 2 | 1 | 0 |
| gi\|4502027 | serum albumin preproprotein [Homo sapiens] | 2 | 2 | 0 | 2 |
| gi\|11415030 (+13) | histone H4 [Homo sapiens] | 2 | 2 | 0 | 0 |
| gi\|574275033 | lymphoid-specific helicase isoform 2 [Homo sapiens] | 2 | 2 | 0 | 0 |
| gi\|25777713 | S-phase kinase-associated protein 1 isoform b [Homo sapiens] | 2 | 3 | 2 | 2 |
| gi\|5803227 | 14-3-3 protein theta [Homo sapiens] | 2 | 3 | 2 | 1 |
| gi\|106049292 (+8) | pyruvate carboxylase, mitochondrial precursor [Homo sapiens] | 2 | 3 | 1 | 1 |
| gi\|14165469 (+1) | 40S ribosomal protein S15a [Homo sapiens] | 2 | 3 | 0 | 0 |
| gi\|15431303 (+2) | 60S ribosomal protein L9 [Homo sapiens] | 2 | 3 | 0 | 2 |
| gi\|5730023 | ruvB-like 2 [Homo sapiens] | 2 | 5 | 2 | 1 |
| gi\|45439306 | aspartate--tRNA ligase, cytoplasmic isoform 1 [Homo sapiens] | 2 | 5 | 1 | 1 |
| gi\|34098946 | nuclease-sensitive element-binding protein 1 [Homo sapiens] | 2 | 6 | 5 | 8 |
| gi\|767994115 (+3) | PREDICTED: centrosomal protein of 131 kDa isoform X1 [Homo sapiens] | 2 | 6 | 4 | 6 |
| gi\|4506649 | 60S ribosomal protein L3 isoform a [Homo sapiens] | 2 | 6 | 2 | 3 |
| gi\|4506597 | 60S ribosomal protein L12 [Homo sapiens] | 2 | 6 | 1 | 2 |
| gi\|15431310 | keratin, type I cytoskeletal 14 [Homo sapiens] | 2 | 8 | 0 | 0 |
| gi\|118572613 (+1) | serine/arginine repetitive matrix protein 2 [Homo sapiens] | 2 | 10 | 1 | 4 |
| gi\|11545863 | methylcrotonoyl-CoA carboxylase beta chain, mitochondrial [Homo sapiens] | 2 | 12 | 2 | 4 |
| gi\|57165424 | cullin-4A isoform 1 [Homo sapiens] | 3 | 0 | 8 | 7 |
| gi\|768024482 (+4) | PREDICTED: SUN domain-containing protein 2 isoform X2 [Homo sapiens] | 3 | 0 | 4 | 3 |
| gi\|37577107 | C-type lectin domain family 2 member B [Homo sapiens] | 3 | 0 | 3 | 4 |
| gi\|4506803 | C-type lectin domain family 11 member A precursor [Homo sapiens] | 3 | 0 | 3 | 4 |
| gi\|209969812 | KN motif and ankyrin repeat domain-containing protein 2 isoform 2 [Homo sapiens] | 3 | 0 | 3 | 3 |
| gi\|530372928 (+2) | PREDICTED: RAF proto-oncogene serine/threonine-protein kinase isoform X3 [Homo sapiens] | 3 | 0 | 3 | 4 |
| gi\|5453559 | ATP synthase subunit d, mitochondrial isoform a [Homo sapiens] | 3 | 0 | 2 | 2 |
| gi\|7019503 (+1) | prolactin regulatory element-binding protein [Homo sapiens] | 3 | 0 | 2 | 0 |
| gi\|29029601 (+1) | probable ATP-dependent RNA helicase DHX37 [Homo sapiens] | 3 | 0 | 2 | 1 |
| gi\|7657236 (+1) | inositol monophosphatase 2 [Homo sapiens] | 3 | 0 | 2 | 1 |
| gi\|289063422 (+1) | phosphorylase b kinase gamma catalytic chain, liver/testis isoform isoform 2 [Homo sapiens] | 3 | 0 | 2 | 0 |
| gi\|567757574 (+2) | squalene synthase isoform 1 [Homo sapiens] | 3 | 0 | 1 | 1 |
| gi\|4503363 (+1) | dolichol-phosphate mannosyltransferase subunit 1 [Homo sapiens] | 3 | 0 | 1 | 1 |
| gi\|33239451 (+1) | proliferating cell nuclear antigen [Homo sapiens] | 3 | 0 | 1 | 0 |
| gi\|578802190 | PREDICTED: protein ELYS isoform X1 [Homo sapiens] | 3 | 0 | 1 | 3 |
| gi\|557878738 (+1) | long-chain-fatty-acid--CoA ligase 1 isoform b [Homo sapiens] | 3 | 0 | 1 | 1 |
| gi\|59710109 | complex I assembly factor TIMMDC1, mitochondrial precursor [Homo sapiens] | 3 | 0 | 1 | 2 |
| gi\|21450775 (+2) | torsin-1A-interacting protein 2 isoform b [Homo sapiens] | 3 | 0 | 1 | 0 |
| gi\|11641243 | peptide deformylase, mitochondrial precursor [Homo sapiens] | 3 | 0 | 1 | 1 |
| gi\|14150017 | transmembrane protein 126A isoform 1 [Homo sapiens] | 3 | 0 | 1 | 0 |
| gi\|530405298 | PREDICTED: disintegrin and metalloproteinase domain-containing protein 10 isoform X1 [Homo sapiens] | 3 | 0 | 1 | 1 |
| gi\|767969893 (+2) | PREDICTED: condensin-2 complex subunit D3 isoform X2 [Homo sapiens] | 3 | 0 | 1 | 2 |
| gi\|4557663 | immunoglobulin-binding protein 1 [Homo sapiens] | 3 | 0 | 1 | 2 |
| gi\|21361796 | ELL-associated factor 2 [Homo sapiens] | 3 | 0 | 1 | 2 |
| gi\|39753961 (+5) | ras GTPase-activating-like protein IQGAP3 [Homo sapiens] | 3 | 0 | 1 | 0 |
| gi\|63162572 | T-complex protein 1 subunit gamma isoform a [Homo sapiens] | 3 | 0 | 0 | 1 |
| gi\|45387945 | extended synaptotagmin-2 [Homo sapiens] | 3 | 0 | 0 | 0 |
| gi\|583966148 | isocitrate dehydrogenase [NADP], mitochondrial isoform 2 [Homo sapiens] | 3 | 0 | 0 | 0 |
| gi\|4503483 | elongation factor 2 [Homo sapiens] | 3 | 0 | 0 | 1 |
| gi\|38016911 | erythrocyte band 7 integral membrane protein isoform a [Homo sapiens] | 3 | 0 | 0 | 0 |
| gi\|4557761 | DNA mismatch repair protein Msh2 isoform 1 [Homo sapiens] | 3 | 0 | 0 | 0 |
| gi\|116642887 | probable hydrolase PNKD isoform 1 precursor [Homo sapiens] | 3 | 0 | 0 | 1 |
| gi\|767953422 (+3) | PREDICTED: maestro heat-like repeat-containing protein family member 6 isoform X6 [Homo sapiens] | 3 | 0 | 0 | 1 |
| gi\|17738292 (+4) | D-beta-hydroxybutyrate dehydrogenase, mitochondrial precursor [Homo sapiens] | 3 | 0 | 0 | 0 |
| gi\|530367753 | PREDICTED: ancient ubiquitous protein 1 isoform X1 [Homo sapiens] | 3 | 0 | 0 | 0 |
| gi\|768036769 (+1) | PREDICTED: E3 ubiquitin-protein ligase HUWE1 isoform X7 [Homo sapiens] | 3 | 0 | 0 | 1 |
| gi\|22748937 | exportin-5 [Homo sapiens] | 3 | 0 | 0 | 0 |
| gi\|11863154 | coatomer subunit delta isoform 1 [Homo sapiens] | 3 | 0 | 0 | 1 |
| gi\|48255945 (+2) | plasma membrane calcium-transporting ATPase 1 isoform 1b [Homo sapiens] | 3 | 0 | 0 | 0 |
| gi\|4758774 | NADH dehydrogenase [ubiquinone] 1 beta subcomplex subunit 10 [Homo sapiens] | 3 | 0 | 0 | 0 |
| gi\|767992145 (+1) | PREDICTED: beta-arrestin-2 isoform X1 [Homo sapiens] | 3 | 0 | 0 | 2 |
| gi\|24308324 (+1) | fatty acyl-CoA reductase 1 [Homo sapiens] | 3 | 0 | 0 | 1 |
| gi\|66363694 | HCLS1-associated protein X-1 isoform b [Homo sapiens] | 3 | 0 | 0 | 0 |
| gi\|557357725 (+1) | adenosine 3'-phospho 5'-phosphosulfate transporter 1 isoform b [Homo sapiens] | 3 | 0 | 0 | 1 |
| gi\|53759151 | acyl-CoA desaturase [Homo sapiens] | 3 | 0 | 0 | 0 |
| gi\|51873031 (+1) | nicalin precursor [Homo sapiens] | 3 | 0 | 0 | 0 |
| gi\|22538431 (+7) | cathepsin B preproprotein [Homo sapiens] | 3 | 0 | 0 | 1 |
| gi\|4502981 (+2) | cytochrome c oxidase subunit 4 isoform 1, mitochondrial precursor [Homo sapiens] | 3 | 0 | 0 | 0 |
| gi\|222537719 | cytoplasmic tRNA 2-thiolation protein 1 [Homo sapiens] | 3 | 0 | 0 | 1 |
| gi\|7108367 | proto-oncogene vav isoform 1 [Homo sapiens] | 3 | 0 | 0 | 0 |
| gi\|4507013 (+1) | solute carrier family 2, facilitated glucose transporter member 5 isoform 1 [Homo sapiens] | 3 | 0 | 0 | 0 |
| gi\|68989256 (+2) | ras and Rab interactor 1 [Homo sapiens] | 3 | 0 | 0 | 0 |
| gi\|578827003 (+2) | PREDICTED: unconventional myosin-IXa isoform X4 [Homo sapiens] | 3 | 0 | 0 | 0 |
| gi\|126722884 (+1) | COBW domain-containing protein 1 isoform 1 [Homo sapiens] | 3 | 0 | 0 | 0 |
| gi\|767939897 | PREDICTED: major histocompatibility complex, class I, B isoform X1 [Homo sapiens] | 3 | 0 | 0 | 0 |
| gi\|11024700 | mitochondrial import inner membrane translocase subunit Tim13 [Homo sapiens] | 3 | 0 | 0 | 2 |
| gi\|7662647 | phosphatidylserine synthase 1 isoform 1 [Homo sapiens] | 3 | 0 | 0 | 0 |
| gi\|7669503 | lysosome-associated membrane glycoprotein 2 isoform B precursor [Homo sapiens] | 3 | 0 | 0 | 0 |
| gi\|578811175 (+2) | PREDICTED: endoplasmic reticulum-Golgi intermediate compartment protein 1 isoform X5 [Homo sapiens] | 3 | 0 | 0 | 0 |
| gi\|160948599 (+1) | integrator complex subunit 1 [Homo sapiens] | 3 | 0 | 0 | 0 |
| gi\|41872631 (+1) | fatty acid synthase [Homo sapiens] | 3 | 0 | 0 | 0 |
| gi\|21618340 (+4) | signal transducer and activator of transcription 3 isoform 1 [Homo sapiens] | 3 | 0 | 0 | 0 |
| gi\|544161315 | GTPase-activating protein and VPS9 domain-containing protein 1 isoform 1 [Homo sapiens] | 3 | 0 | 0 | 3 |
| gi\|767914424 | PREDICTED: hexokinase-2 isoform X2 [Homo sapiens] | 3 | 0 | 0 | 0 |
| gi\|321117185 (+2) | ubiquitin-conjugating enzyme E2 E1 isoform 3 [Homo sapiens] | 3 | 0 | 0 | 0 |
| gi\|530401025 (+1) | PREDICTED: squamous cell carcinoma antigen recognized by T-cells 3 isoform X1 [Homo sapiens] | 3 | 0 | 0 | 0 |
| gi\|20127652 (+1) | presenilins-associated rhomboid-like protein, mitochondrial isoform 1 preproprotein [Homo sapiens] | 3 | 0 | 0 | 0 |
| gi\|198041777 (+1) | importin-11 isoform 1 [Homo sapiens] | 3 | 0 | 0 | 0 |
| gi\|21450665 (+2) | coiled-coil domain-containing protein 138 isoform 1 [Homo sapiens] | 3 | 0 | 0 | 1 |
| gi\|37577122 (+1) | ubiquitin-conjugating enzyme E2 J1 [Homo sapiens] | 3 | 0 | 0 | 0 |
| gi\|768008670 (+1) | PREDICTED: striatin-4 isoform X3 [Homo sapiens] | 3 | 0 | 0 | 0 |
| gi\|53759103 | importin-8 isoform 1 [Homo sapiens] | 3 | 0 | 0 | 0 |
| gi\|530432783 (+1) | PREDICTED: dixin isoform X1 [Homo sapiens] | 3 | 0 | 0 | 0 |
| gi\|40068497 (+1) | FAST kinase domain-containing protein 3 [Homo sapiens] | 3 | 0 | 0 | 0 |
| gi\|108773787 (+1) | retinoblastoma-associated protein [Homo sapiens] | 3 | 0 | 0 | 0 |
| gi\|218931253 | sn1-specific diacylglycerol lipase beta isoform 2 [Homo sapiens] | 3 | 0 | 0 | 0 |
| gi\|41152072 (+3) | palmitoyltransferase ZDHHC5 [Homo sapiens] | 3 | 0 | 0 | 1 |
| gi\|25777677 (+5) | ras association domain-containing protein 2 [Homo sapiens] | 3 | 0 | 0 | 0 |
| gi\|332000015 (+7) | 5'-AMP-activated protein kinase subunit gamma-1 isoform 3 [Homo sapiens] | 3 | 0 | 0 | 0 |
| gi\|7262393 | ATP-binding cassette sub-family D member 1 [Homo sapiens] | 3 | 1 | 3 | 0 |
| gi\|17158044 | 40S ribosomal protein S6 [Homo sapiens] | 3 | 1 | 2 | 2 |
| gi\|48526509 | mitochondrial import inner membrane translocase subunit TIM50 [Homo sapiens] | 3 | 1 | 1 | 3 |
| gi\|4507131 | small nuclear ribonucleoprotein F [Homo sapiens] | 3 | 1 | 1 | 1 |
| gi\|578836126 (+1) | PREDICTED: dolichyl-diphosphooligosaccharide--protein glycosyltransferase subunit 2 isoform X1 [Homo sapiens] | 3 | 1 | 0 | 0 |
| gi\|4502303 | ATP synthase subunit O, mitochondrial precursor [Homo sapiens] | 3 | 1 | 0 | 0 |
| gi\|14277700 | 40S ribosomal protein S12 [Homo sapiens] | 3 | 1 | 0 | 0 |
| gi\|31881785 | Hermansky-Pudlak syndrome 6 protein [Homo sapiens] | 3 | 1 | 0 | 0 |
| gi\|226246671 (+1) | 40S ribosomal protein S20 isoform 1 [Homo sapiens] | 3 | 2 | 3 | 4 |
| gi\|422398883 (+5) | cytoplasmic dynein 1 intermediate chain 2 isoform 2 [Homo sapiens] | 3 | 2 | 1 | 2 |
| gi\|313569768 (+7) | 60S ribosomal protein L17 isoform a [Homo sapiens] | 3 | 2 | 1 | 2 |
| gi\|14043022 | methionine--tRNA ligase, cytoplasmic [Homo sapiens] | 3 | 2 | 0 | 0 |
| gi\|66932947 | alpha-2-macroglobulin precursor [Homo sapiens] | 3 | 3 | 4 | 2 |
| gi\|4503149 (+1) | cathepsin G preproprotein [Homo sapiens] | 3 | 4 | 3 | 3 |
| gi\|4506723 | 40S ribosomal protein S3a isoform 1 [Homo sapiens] | 3 | 4 | 2 | 6 |
| gi\|4506607 | 60S ribosomal protein L18 isoform 1 [Homo sapiens] | 3 | 5 | 1 | 3 |
| gi\|116805327 (+1) | methylcrotonoyl-CoA carboxylase subunit alpha, mitochondrial isoform 1 precursor [Homo sapiens] | 3 | 6 | 1 | 1 |
| gi\|55956788 | nucleolin [Homo sapiens] | 3 | 9 | 0 | 3 |
| gi\|530412176 | PREDICTED: keratin, type I cytoskeletal 10 isoform X1 [Homo sapiens] | 3 | 11 | 7 | 15 |
| gi\|55956899 | keratin, type I cytoskeletal 9 [Homo sapiens] | 3 | 19 | 3 | 6 |
| gi\|119395750 | keratin, type II cytoskeletal 1 [Homo sapiens] | 3 | 35 | 7 | 19 |
| gi\|115583685 (+5) | monocarboxylate transporter 1 [Homo sapiens] | 4 | 0 | 4 | 3 |
| gi\|530414178 | PREDICTED: mitochondrial import inner membrane translocase subunit Tim21 isoform X1 [Homo sapiens] | 4 | 0 | 4 | 2 |
| gi\|13375983 (+5) | mitochondrial glutamate carrier 1 [Homo sapiens] | 4 | 0 | 3 | 6 |
| gi\|119943112 (+1) | 7-dehydrocholesterol reductase [Homo sapiens] | 4 | 0 | 2 | 1 |
| gi\|21359867 | cytochrome c1, heme protein, mitochondrial precursor [Homo sapiens] | 4 | 0 | 2 | 2 |
| gi\|207113160 (+1) | treacle protein isoform d [Homo sapiens] | 4 | 0 | 2 | 9 |
| gi\|148491070 (+4) | CTP synthase 1 isoform a [Homo sapiens] | 4 | 0 | 1 | 2 |
| gi\|284795266 | signal recognition particle receptor subunit beta [Homo sapiens] | 4 | 0 | 1 | 4 |
| gi\|359718987 (+1) | ATP-dependent zinc metalloprotease YME1L1 isoform 4 [Homo sapiens] | 4 | 0 | 1 | 2 |
| gi\|325301072 (+3) | translocon-associated protein subunit delta isoform 1 precursor [Homo sapiens] | 4 | 0 | 1 | 0 |
| gi\|223278379 | RRP12-like protein isoform 1 [Homo sapiens] | 4 | 0 | 1 | 6 |
| gi\|32189371 (+1) | transmembrane protein 165 precursor [Homo sapiens] | 4 | 0 | 1 | 2 |
| gi\|530387549 (+1) | PREDICTED: erlin-2 isoform X1 [Homo sapiens] | 4 | 0 | 1 | 1 |
| gi\|21327667 | ribosome biogenesis protein BOP1 [Homo sapiens] | 4 | 0 | 1 | 0 |
| gi\|154354966 | MICOS complex subunit MIC60 isoform 3 [Homo sapiens] | 4 | 0 | 0 | 0 |
| gi\|13399298 (+1) | immunoglobulin lambda-like polypeptide 1 isoform a precursor [Homo sapiens] | 4 | 0 | 0 | 1 |
| gi\|68800243 | interleukin-1 receptor-associated kinase 1 isoform 1 [Homo sapiens] | 4 | 0 | 0 | 0 |
| gi\|166795297 | cytoplasmic dynein 1 light intermediate chain 1 [Homo sapiens] | 4 | 0 | 0 | 2 |
| gi\|5174447 | guanine nucleotide-binding protein subunit beta-2-like 1 [Homo sapiens] | 4 | 0 | 0 | 0 |
| gi\|411147363 (+2) | ATP-binding cassette sub-family B member 7, mitochondrial isoform 2 [Homo sapiens] | 4 | 0 | 0 | 0 |
| gi\|578831151 | PREDICTED: 26S protease regulatory subunit 8 isoform X1 [Homo sapiens] | 4 | 0 | 0 | 0 |
| gi\|62955803 | nucleoporin NUP188 homolog [Homo sapiens] | 4 | 0 | 0 | 1 |
| gi\|4507879 (+1) | voltage-dependent anion-selective channel protein 1 [Homo sapiens] | 4 | 0 | 0 | 0 |
| gi\|166795299 | solute carrier family 2, facilitated glucose transporter member 1 [Homo sapiens] | 4 | 0 | 0 | 0 |
| gi\|315259086 (+3) | integrator complex subunit 7 isoform 2 [Homo sapiens] | 4 | 0 | 0 | 0 |
| gi\|301129187 | NADH dehydrogenase [ubiquinone] 1 alpha subcomplex subunit 11 isoform 2 [Homo sapiens] | 4 | 0 | 0 | 0 |
| gi\|195972896 (+3) | thromboxane-A synthase isoform 1 [Homo sapiens] | 4 | 0 | 0 | 0 |
| gi\|5453660 (+1) | gamma-tubulin complex component 3 isoform 1 [Homo sapiens] | 4 | 0 | 0 | 0 |
| gi\|767980369 | PREDICTED: striatin-3 isoform X4 [Homo sapiens] | 4 | 0 | 0 | 0 |
| gi\|48255957 | plasma membrane calcium-transporting ATPase 4 isoform 4b [Homo sapiens] | 4 | 0 | 0 | 0 |
| gi\|530339581 (+2) | condensin-2 complex subunit G2 isoform b [Homo sapiens] | 4 | 0 | 0 | 0 |
| gi\|38327039 | heat shock 70 kDa protein 4 [Homo sapiens] | 4 | 0 | 0 | 0 |
| gi\|359751462 (+3) | UDP-glucuronic acid decarboxylase 1 isoform 1 [Homo sapiens] | 4 | 0 | 0 | 0 |
| gi\|23308607 | minor histocompatibility antigen H13 isoform 1 [Homo sapiens] | 4 | 0 | 0 | 0 |
| gi\|19913371 (+10) | F-box-like/WD repeat-containing protein TBL1XR1 [Homo sapiens] | 4 | 0 | 0 | 0 |
| gi\|21361659 | importin-9 [Homo sapiens] | 4 | 0 | 0 | 0 |
| gi\|4506711 | 40S ribosomal protein S27 [Homo sapiens] | 4 | 1 | 3 | 4 |
| gi\|371875793 (+5) | serine/threonine-protein kinase A-Raf isoform 2 [Homo sapiens] | 4 | 1 | 1 | 3 |
| gi\|12056465 (+2) | rRNA 2'-O-methyltransferase fibrillarin [Homo sapiens] | 4 | 1 | 0 | 4 |
| gi\|19387846 (+2) | melanoma-associated antigen D2 [Homo sapiens] | 4 | 1 | 0 | 0 |
| gi\|15451765 | protein PML isoform 9 [Homo sapiens] | 4 | 2 | 9 | 7 |
| gi\|109288010 (+6) | monocarboxylate transporter 4 [Homo sapiens] | 4 | 2 | 4 | 4 |
| gi\|4758504 | 3-hydroxyacyl-CoA dehydrogenase type-2 isoform 1 [Homo sapiens] | 4 | 2 | 1 | 1 |
| gi\|14141193 (+19) | 40S ribosomal protein S9 [Homo sapiens] | 4 | 2 | 0 | 2 |
| gi\|194733742 (+2) | negative elongation factor A [Homo sapiens] | 4 | 2 | 0 | 0 |
| gi\|5453621 | CMP-sialic acid transporter isoform a [Homo sapiens] | 4 | 2 | 0 | 0 |
| gi\|316659409 (+1) | actin, cytoplasmic 2 [Homo sapiens] | 4 | 3 | 6 | 6 |
| gi\|4505775 (+1) | phosphate carrier protein, mitochondrial isoform b precursor [Homo sapiens] | 4 | 4 | 4 | 3 |
| gi\|394582093 | DNA replication licensing factor MCM3 isoform 1 [Homo sapiens] | 4 | 9 | 2 | 10 |
| gi\|7706254 | nucleolar protein 58 [Homo sapiens] | 5 | 0 | 5 | 3 |
| gi\|24430146 (+3) | nuclear pore complex protein Nup153 isoform 2 [Homo sapiens] | 5 | 0 | 4 | 7 |
| gi\|122939157 (+7) | gem-associated protein 4 [Homo sapiens] | 5 | 0 | 3 | 2 |
| gi\|4758340 | phenylalanine--tRNA ligase alpha subunit [Homo sapiens] | 5 | 0 | 2 | 1 |
| gi\|154800487 (+2) | erlin-1 [Homo sapiens] | 5 | 0 | 2 | 2 |
| gi\|23308577 | D-3-phosphoglycerate dehydrogenase [Homo sapiens] | 5 | 0 | 1 | 0 |
| gi\|19923315 (+3) | serine hydroxymethyltransferase, mitochondrial isoform 1 precursor [Homo sapiens] | 5 | 0 | 1 | 1 |
| gi\|33636719 | mitochondrial import inner membrane translocase subunit TIM44 [Homo sapiens] | 5 | 0 | 1 | 4 |
| gi\|205360838 | dnaJ homolog subfamily A member 3, mitochondrial isoform 1 [Homo sapiens] | 5 | 0 | 0 | 2 |
| gi\|42476022 (+1) | tetratricopeptide repeat protein 27 isoform 1 [Homo sapiens] | 5 | 0 | 0 | 0 |
| gi\|39652628 (+3) | constitutive coactivator of PPAR-gamma-like protein 1 isoform a [Homo sapiens] | 5 | 0 | 0 | 1 |
| gi\|41350216 | chitobiosyldiphosphodolichol beta-mannosyltransferase [Homo sapiens] | 5 | 0 | 0 | 0 |
| gi\|190194355 (+1) | protein CIP2A [Homo sapiens] | 5 | 0 | 0 | 0 |
| gi\|22095331 (+3) | ER membrane protein complex subunit 1 isoform 1 precursor [Homo sapiens] | 5 | 0 | 0 | 0 |
| gi\|225545550 (+4) | telomere length regulation protein TEL2 homolog [Homo sapiens] | 5 | 0 | 0 | 0 |
| gi\|30410794 (+1) | proteasome activator complex subunit 3 isoform 1 [Homo sapiens] | 5 | 0 | 0 | 0 |
| gi\|530383409 | PREDICTED: absent in melanoma 1 protein isoform X1 [Homo sapiens] | 5 | 0 | 0 | 0 |
| gi\|155030234 (+3) | ribonuclease 3 isoform 1 [Homo sapiens] | 5 | 0 | 0 | 1 |
| gi\|52632377 (+3) | melanoma-associated antigen D1 isoform b [Homo sapiens] | 5 | 0 | 0 | 1 |
| gi\|4505725 (+1) | peroxisome biogenesis factor 1 isoform 1 [Homo sapiens] | 5 | 0 | 0 | 0 |
| gi\|21361315 | transmembrane 9 superfamily member 1 isoform a precursor [Homo sapiens] | 5 | 0 | 0 | 0 |
| gi\|256818821 | E3 ubiquitin-protein ligase RAD18 [Homo sapiens] | 5 | 0 | 0 | 0 |
| gi\|148536853 | coatomer subunit alpha isoform 2 [Homo sapiens] | 5 | 1 | 3 | 0 |
| gi\|170763479 (+2) | MMS19 nucleotide excision repair protein homolog isoform 1 [Homo sapiens] | 5 | 1 | 0 | 1 |
| gi\|4506127 | ribose-phosphate pyrophosphokinase 1 isoform 1 [Homo sapiens] | 5 | 1 | 0 | 2 |
| gi\|281604136 (+1) | serine/threonine-protein phosphatase PGAM5, mitochondrial isoform 1 [Homo sapiens] | 5 | 2 | 2 | 4 |
| gi\|528281413 (+1) | trifunctional enzyme subunit beta, mitochondrial isoform 3 [Homo sapiens] | 5 | 2 | 1 | 1 |
| gi\|4506691 | 40S ribosomal protein S16 [Homo sapiens] | 5 | 4 | 3 | 4 |
| gi\|530421596 | PREDICTED: probable ubiquitin carboxyl-terminal hydrolase FAF-X isoform X2 [Homo sapiens] | 5 | 6 | 3 | 1 |
| gi\|62241042 | bifunctional glutamate/proline--tRNA ligase [Homo sapiens] | 5 | 13 | 2 | 4 |
| gi\|768002555 (+3) | unconventional myosin-IXb isoform 1 [Homo sapiens] | 6 | 0 | 12 | 17 |
| gi\|217272892 (+2) | 116 kDa U5 small nuclear ribonucleoprotein component isoform a [Homo sapiens] | 6 | 0 | 4 | 7 |
| gi\|60499021 (+1) | ferrochelatase, mitochondrial isoform b precursor [Homo sapiens] | 6 | 0 | 3 | 4 |
| gi\|21361103 | calcium-binding mitochondrial carrier protein Aralar1 [Homo sapiens] | 6 | 0 | 2 | 2 |
| gi\|33469968 | DNA replication licensing factor MCM7 isoform 1 [Homo sapiens] | 6 | 0 | 1 | 4 |
| gi\|32189394 | ATP synthase subunit beta, mitochondrial precursor [Homo sapiens] | 6 | 0 | 1 | 2 |
| gi\|38372921 | basigin isoform 3 [Homo sapiens] | 6 | 0 | 1 | 1 |
| gi\|194306640 (+2) | dnaJ homolog subfamily B member 12 [Homo sapiens] | 6 | 0 | 1 | 4 |
| gi\|4758334 | fatty acid desaturase 2 isoform 1 [Homo sapiens] | 6 | 0 | 0 | 0 |
| gi\|4506031 | palmitoyl-protein thioesterase 1 isoform 1 precursor [Homo sapiens] | 6 | 0 | 0 | 2 |
| gi\|157671927 (+1) | spermatogenesis-associated protein 5 [Homo sapiens] | 6 | 0 | 0 | 2 |
| gi\|767951589 | PREDICTED: brefeldin A-inhibited guanine nucleotide-exchange protein 1 isoform X4 [Homo sapiens] | 6 | 0 | 0 | 0 |
| gi\|109150419 (+3) | WD repeat-containing protein mio [Homo sapiens] | 6 | 0 | 0 | 0 |
| gi\|30089954 | DDB1- and CUL4-associated factor 8 [Homo sapiens] | 6 | 0 | 0 | 0 |
| gi\|187608615 (+1) | non-receptor tyrosine-protein kinase TYK2 [Homo sapiens] | 6 | 0 | 0 | 0 |
| gi\|7019415 | protein transport protein Sec61 subunit alpha isoform 1 [Homo sapiens] | 6 | 1 | 4 | 3 |
| gi\|5031741 | dnaJ homolog subfamily A member 2 [Homo sapiens] | 6 | 1 | 3 | 7 |
| gi\|50592988 | cytochrome b-c1 complex subunit 2, mitochondrial precursor [Homo sapiens] | 6 | 1 | 2 | 2 |
| gi\|4504151 (+1) | granulins precursor [Homo sapiens] | 6 | 1 | 1 | 1 |
| gi\|530396298 (+1) | PREDICTED: carnitine O-palmitoyltransferase 1, liver isoform isoform X1 [Homo sapiens] | 6 | 2 | 2 | 2 |
| gi\|15147337 (+1) | E3 ubiquitin-protein ligase UBR5 isoform 1 [Homo sapiens] | 6 | 3 | 24 | 52 |
| gi\|4504517 | heat shock protein beta-1 [Homo sapiens] | 6 | 5 | 1 | 3 |
| gi\|31542947 (+2) | 60 kDa heat shock protein, mitochondrial [Homo sapiens] | 6 | 87 | 6 | 7 |
| gi\|34147630 | elongation factor Tu, mitochondrial precursor [Homo sapiens] | 7 | 0 | 7 | 7 |
| gi\|119964726 | cation-independent mannose-6-phosphate receptor precursor [Homo sapiens] | 7 | 0 | 5 | 7 |
| gi\|47458811 | sideroflexin-4 [Homo sapiens] | 7 | 0 | 2 | 0 |
| gi\|4503895 | galactokinase [Homo sapiens] | 7 | 0 | 1 | 4 |
| gi\|12408656 (+4) | calpain-1 catalytic subunit [Homo sapiens] | 7 | 0 | 1 | 2 |
| gi\|31880783 | protein pelota homolog [Homo sapiens] | 7 | 0 | 1 | 2 |
| gi\|11321607 (+1) | pachytene checkpoint protein 2 homolog isoform 1 [Homo sapiens] | 7 | 0 | 0 | 0 |
| gi\|48762920 | ATP-dependent 6-phosphofructokinase, liver type isoform b [Homo sapiens] | 7 | 1 | 5 | 6 |
| gi\|24475816 | very-long-chain enoyl-CoA reductase [Homo sapiens] | 7 | 1 | 4 | 3 |
| gi\|4504511 | dnaJ homolog subfamily A member 1 [Homo sapiens] | 7 | 1 | 4 | 6 |
| gi\|9966805 | ATP-dependent RNA helicase DDX24 [Homo sapiens] | 7 | 1 | 2 | 2 |
| gi\|530415148 (+3) | PREDICTED: ubiquitin-60S ribosomal protein L40 isoform X1 [Homo sapiens] | 7 | 3 | 2 | 0 |
| gi\|20127408 | trifunctional enzyme subunit alpha, mitochondrial precursor [Homo sapiens] | 7 | 4 | 4 | 6 |
| gi\|11968182 | 40S ribosomal protein S18 [Homo sapiens] | 7 | 4 | 4 | 8 |
| gi\|10864011 (+1) | sulfide:quinone oxidoreductase, mitochondrial [Homo sapiens] | 7 | 5 | 5 | 7 |
| gi\|767992199 (+1) | PREDICTED: sarcoplasmic/endoplasmic reticulum calcium ATPase 3 isoform X5 [Homo sapiens] | 8 | 0 | 3 | 1 |
| gi\|21361647 (+1) | putative adenosylhomocysteinase 2 isoform a [Homo sapiens] | 8 | 0 | 1 | 0 |
| gi\|221316630 (+2) | coatomer subunit beta [Homo sapiens] | 8 | 0 | 0 | 0 |
| gi\|45597175 (+1) | TBC1 domain family member 9B isoform a [Homo sapiens] | 8 | 0 | 0 | 0 |
| gi\|4506675 | dolichyl-diphosphooligosaccharide--protein glycosyltransferase subunit 1 precursor [Homo sapiens] | 8 | 1 | 3 | 2 |
| gi\|157694492 (+1) | myb-binding protein 1A isoform 2 [Homo sapiens] | 8 | 2 | 0 | 0 |
| gi\|156071462 | ADP/ATP translocase 3 [Homo sapiens] | 8 | 3 | 0 | 7 |
| gi\|4506695 | 40S ribosomal protein S19 [Homo sapiens] | 8 | 4 | 0 | 2 |
| gi\|283436224 | ATPase family AAA domain-containing protein 3A isoform 3 [Homo sapiens] | 8 | 10 | 0 | 0 |
| gi\|767949111 | PREDICTED: ubiquitin-protein ligase E3C isoform X1 [Homo sapiens] | 9 | 0 | 2 | 3 |
| gi\|73695475 (+1) | HEAT repeat-containing protein 1 [Homo sapiens] | 9 | 0 | 0 | 0 |
| gi\|4885399 | structural maintenance of chromosomes protein 3 [Homo sapiens] | 9 | 0 | 0 | 1 |
| gi\|613410229 | estradiol 17-beta-dehydrogenase 11 precursor [Homo sapiens] | 9 | 0 | 0 | 1 |
| gi\|164607124 (+7) | Fanconi anemia group I protein isoform 1 [Homo sapiens] | 9 | 0 | 0 | 0 |
| gi\|4506725 | 40S ribosomal protein S4, X isoform X isoform [Homo sapiens] | 9 | 4 | 12 | 12 |
| gi\|4757810 (+1) | ATP synthase subunit alpha, mitochondrial isoform a precursor [Homo sapiens] | 9 | 5 | 9 | 9 |
| gi\|42542392 | CCAAT/enhancer-binding protein zeta [Homo sapiens] | 10 | 0 | 4 | 2 |
| gi\|237649019 (+2) | calcium-binding mitochondrial carrier protein Aralar2 isoform 1 [Homo sapiens] | 10 | 0 | 3 | 1 |
| gi\|50345988 (+1) | ATP synthase subunit gamma, mitochondrial isoform L (liver) precursor [Homo sapiens] | 10 | 1 | 3 | 5 |
| gi\|4503471 (+1) | elongation factor 1-alpha 1 [Homo sapiens] | 10 | 2 | 15 | 15 |
| gi\|24234688 | stress-70 protein, mitochondrial precursor [Homo sapiens] | 10 | 8 | 4 | 5 |
| gi\|148529014 | DNA damage-binding protein 1 [Homo sapiens] | 10 | 8 | 2 | 1 |
| gi\|8051636 | exportin-T [Homo sapiens] | 11 | 0 | 0 | 0 |
| gi\|14150070 | coiled-coil-helix-coiled-coil-helix domain-containing protein 5 isoform a [Homo sapiens] | 11 | 0 | 0 | 0 |
| gi\|63252886 | prolyl 4-hydroxylase subunit alpha-1 isoform 1 precursor [Homo sapiens] | 11 | 4 | 18 | 16 |
| gi\|31621305 | leucine-rich PPR motif-containing protein, mitochondrial precursor [Homo sapiens] | 11 | 9 | 4 | 5 |
| gi\|25777612 | 26S proteasome non-ATPase regulatory subunit 3 [Homo sapiens] | 12 | 0 | 4 | 3 |
| gi\|208609990 (+1) | nuclear pore complex protein Nup93 isoform 1 [Homo sapiens] | 12 | 0 | 0 | 3 |
| gi\|14210536 (+1) | tubulin beta-6 chain isoform 1 [Homo sapiens] | 12 | 4 | 7 | 0 |
| gi\|156071459 | ADP/ATP translocase 2 [Homo sapiens] | 12 | 5 | 10 | 10 |
| gi\|15718687 (+1) | 40S ribosomal protein S3 isoform 1 [Homo sapiens] | 12 | 12 | 17 | 16 |
| gi\|57863257 | T-complex protein 1 subunit alpha isoform a [Homo sapiens] | 13 | 0 | 5 | 5 |
| gi\|157694511 | nucleolar complex protein 2 homolog [Homo sapiens] | 14 | 0 | 1 | 4 |
| gi\|30581135 | structural maintenance of chromosomes protein 1A isoform 1 [Homo sapiens] | 14 | 1 | 0 | 0 |
| gi\|16507237 | 78 kDa glucose-regulated protein precursor [Homo sapiens] | 14 | 16 | 11 | 18 |
| gi\|21361181 (+1) | sodium/potassium-transporting ATPase subunit alpha-1 isoform a [Homo sapiens] | 15 | 1 | 11 | 15 |
| gi\|283436222 | ATPase family AAA domain-containing protein 3A isoform 2 [Homo sapiens] | 15 | 12 | 12 | 16 |
| gi\|20149594 (+3) | heat shock protein HSP 90-beta isoform a [Homo sapiens] | 16 | 4 | 7 | 9 |
| gi\|530392189 | PREDICTED: ATP-dependent 6-phosphofructokinase, platelet type isoform X1 [Homo sapiens] | 16 | 7 | 12 | 10 |
| gi\|57634534 | nuclear pore complex protein Nup205 [Homo sapiens] | 18 | 0 | 0 | 0 |
| gi\|24638454 | sarcoplasmic/endoplasmic reticulum calcium ATPase 2 isoform b [Homo sapiens] | 20 | 0 | 4 | 5 |
| gi\|24415404 | midasin [Homo sapiens] | 20 | 0 | 0 | 1 |
| gi\|578815313 (+1) | PREDICTED: pericentriolar material 1 protein isoform X4 [Homo sapiens] | 20 | 1 | 3 | 5 |
| gi\|33350932 | cytoplasmic dynein 1 heavy chain 1 [Homo sapiens] | 21 | 0 | 2 | 2 |
| gi\|18105007 | CAD protein [Homo sapiens] | 22 | 3 | 5 | 8 |
| gi\|50592996 | tubulin beta-3 chain isoform 1 [Homo sapiens] | 23 | 9 | 0 | 11 |
| gi\|62460637 | importin-4 [Homo sapiens] | 25 | 0 | 0 | 0 |
| gi\|54607053 | translational activator GCN1 [Homo sapiens] | 29 | 0 | 0 | 0 |
| gi\|5729877 (+1) | heat shock cognate 71 kDa protein isoform 1 [Homo sapiens] | 30 | 18 | 21 | 14 |
| gi\|767903424 (+1) | PREDICTED: E3 ubiquitin-protein ligase UBR4 isoform X10 [Homo sapiens] | 32 | 7 | 1 | 0 |
| gi\|5174735 | tubulin beta-4B chain [Homo sapiens] | 36 | 15 | 19 | 24 |
| gi\|17921989 (+2) | tubulin alpha-4A chain isoform 1 [Homo sapiens] | 38 | 14 | 18 | 24 |
| gi\|29788785 | tubulin beta chain isoform b [Homo sapiens] | 39 | 17 | 24 | 26 |
| gi\|17986283 (+2) | tubulin alpha-1A chain isoform 1 [Homo sapiens] | 41 | 12 | 16 | 0 |
| gi\|57013276 | tubulin alpha-1B chain [Homo sapiens] | 45 | 15 | 18 | 27 |
| gi\|13654237 (+1) | DNA-dependent protein kinase catalytic subunit isoform 1 [Homo sapiens] | 66 | 5 | 18 | 22 |
